# Concomitant Duplications of Opioid Peptide and Receptor Genes before the Origin of Jawed Vertebrates

**DOI:** 10.1371/journal.pone.0010512

**Published:** 2010-05-06

**Authors:** Görel Sundström, Susanne Dreborg, Dan Larhammar

**Affiliations:** Department of Neuroscience, Uppsala University, Uppsala, Sweden; American Museum of Natural History, United States of America

## Abstract

**Background:**

The opioid system is involved in reward and pain mechanisms and consists in mammals of four receptors and several peptides. The peptides are derived from four prepropeptide genes, *PENK, PDYN, PNOC* and *POMC*, encoding enkephalins, dynorphins, orphanin/nociceptin and beta-endorphin, respectively. Previously we have described how two rounds of genome doubling (2R) before the origin of jawed vertebrates formed the receptor family.

**Methodology/Principal Findings:**

Opioid peptide gene family members were investigated using a combination of sequence-based phylogeny and chromosomal locations of the peptide genes in various vertebrates. Several adjacent gene families were investigated similarly. The results show that the ancestral peptide gene gave rise to two additional copies in the genome doublings. The fourth member was generated by a local gene duplication, as the genes encoding *POMC* and *PNOC* are located on the same chromosome in the chicken genome and all three teleost genomes that we have studied. A translocation has disrupted this synteny in mammals. The *PDYN* gene seems to have been lost in chicken, but not in zebra finch. Duplicates of some peptide genes have arisen in the teleost fishes. Within the prepropeptide precursors, peptides have been lost or gained in different lineages.

**Conclusions/Significance:**

The ancestral peptide and receptor genes were located on the same chromosome and were thus duplicated concomitantly. However, subsequently genetic linkage has been lost. In conclusion, the system of opioid peptides and receptors was largely formed by the genome doublings that took place early in vertebrate evolution.

## Introduction

The opioid peptides are derived from four different precursors encoded by four different genes: proenkephalin (*PENK*), prodynorphin (*PDYN*), proopioimelanocortin (*POMC*) and proorphanin (*PNOC*). These genes all share the same overall structure with a single intron in the coding region. The propeptides have conserved cysteines in the N-terminal region and contain one or more opioid “core” sequences consisting of the peptide motif Y/FGGF.

The opioid peptide genes and their products, Met- and Leu-enkephalin from *PENK*, α-neoendorphin, dynorphin A and dynorphin B from *PDYN*, β-endorphin from *POMC* and orphanin/nociceptin and nociceptin-like peptide from *PNOC*, mediate a variety of functions and are involved in both pain and reward pathways. The physiological effects of this peptide-receptor system have been studied extensively, particularly in mammals [1], [2], [3]. Opioid peptides have been identified in all present vertebrate lineages. However, the reports of opioid peptide material in invertebrates [4], [5], [6] have not been confirmed by gene cloning of opioid peptides nor receptors.

We have previously reported that the genes for the four opioid receptors, mu, delta, kappa and orphanin arose from an ancestral receptor gene as a result of two whole-genome duplications early in vertebrate evolution [7]. The family of opioid peptides likewise has four members in mammals which suggest that they too arose in the genome doubling events. Previous analyses have suggested that the opioid family expanded by serial duplications [8] but no comprehensive investigations that takes also chromosomal location into consideration have been reported.

The theory of two rounds of whole genome duplication (2R) early in vertebrate evolution was first proposed in the 1960s and in-depth studies of specific gene families together with sequencing of whole genomes have subsequently strengthened the hypothesis [9]. The sequencing of the whole genome of the basal chordate amphioxus finally confirmed the hypothesis of a quadrupled genome in vertebrates [10]. The genome of the ancestor of teleost fishes doubled once more in an event called 3R [11]. A set of related chromosome regions is called a paralagon. We have previously reported that other receptor-peptide systems expanded in 2R and 3R such as the neuropeptide Y family and its receptors [12], [13], [14] and the tachykinin prepropeptides and receptors [15]. Other receptor-ligand pairs that appear to have expanded in these time periods are the secretin system [16], [17] and the endothelin system [18], [19]. Here we present our analyses of the opioid peptide genes and conclude that they have duplicated concomitantly with the receptor genes in the 2R/3R genome doubling events.

## Methods

### Identification of opioid peptide genes

The opioid prepropeptide genes *(PENK*, *PDYN*, *PNOC* and *POMC*) were identified in the Ensembl database release 46 (www.ensembl.org) and protein sequences from the following species were retrieved: human (*Homo sapiens*), mouse (*Mus musculus*), dog (*Canis familiaris*), grey short-tailed opossum (*Monodelphis domestica*), chicken (*Gallus gallus*), western clawed frog (*Silurana (Xenopus) tropicalis*), zebrafish (*Danio rerio*), medaka (*Oryzias latipes*) and stickleback (*Gasterosteus aculeatus*). BLAST searches [20] were performed to identify non-annotated genes in the database for the species included in the study and in the genomes of zebra finch (*Taeniopygia guttata*) and sea lamprey (*Petromyzon marinus*). In some cases we were compelled to manually curate the sequences. Accession numbers for all sequences are available in Table S1.

### Phylogenetic analysis of opioid peptides

The protein sequences for PENK, PDYN, PNOC and POMC in all species were aligned separately using the Windows version of Clustal X 1.81 [21], [22]. The alignments were edited manually. The curated separate peptide alignments were added one by one to each other using the profile alignment tool in Clustal X 1.81. The final alignment (see Fig. S1) was edited manually and used for the phylogenetic analysis. Two types of trees were constructed: a neighbor-joining tree with 1000 bootstrap replicas using standard settings (Gonnet weight matrix, gap opening penalty 10.0 and gap extension penalty 0.20) in Clustal X 1.81 and a quartet-puzzling maximum likelihood tree using the Windows version of Treepuzzle 5.2 [23]. The analysis was made using the JTT model of amino acid substitution, with the amino acid frequencies estimated from the data set. The model of rate heterogeneity was set to gamma distributed rates with eight gamma rate categories and the alpha parameter estimated from the data set. Parameters were estimated using the “exact” and “quartet sampling + NJ tree” options and the number of puzzling steps were automatically decided by Treepuzzle and varied between 1000 and 25000 depending on the dataset.

### Selection of genomic regions

In our previous study of the opioid receptors we discovered that the mammalian chromosomes have been extensively rearranged compared to the chicken chromosomes [7], which is why the latter constitutes a better starting point for tracing the ancestral chromosome arrangement. In chicken, *PENK* and the opioid kappa receptor (*OPRK*) are located on chromosome 2 and both *PNOC* and *POMC* along with the opioid mu receptor (*OPRM*) are located on chromosome 3. A list with all genes on chicken chromosome 3 in the region 41,94 to 113,66 Mb (corresponding to the genomic region 10 Mb before the *OPRM* gene until 10 Mb after *PNOC* and *POMC*) was downloaded from Ensembl release 46 as were all genes on chromosome 2 in the region 103,64 to 125,07 Mb (10 Mb before the *OPRK* gene until 10 Mb after *PENK*). The opioid receptors in chicken are located in a paralagon that consists of the chromosomes 2, 3, 20 and 23 [7]. With the hypothesis that the opioid receptors and peptides are located in the same paralagon, lists with all genes from chicken chromosomes 20 and 23 were collected from Ensembl release 46. We investigated these lists and continued with phylogenetic analysis of the gene families that fulfilled our selection criteria, i.e. they belong to Ensembl protein families with members in the selected areas on at least three of the chromosomes. Not counting the opioid peptides, 32 families fulfilled the selection criteria.

### Phylogenetic analysis of neighboring families

Protein sequences for all members in the selected protein families were downloaded from the following genomes: human, dog, grey short-tailed opossum, chicken, zebrafish, stickleback and medaka in the Ensembl database release 46. In order to relatively date the trees we included sequences from *Ciona intestinalis* and *Drosophila melanogaster*. BLAST-searches (blastp) against the genome database for the cephalochordate *Branchiostoma floridae* (*Branchiostoma floridae v1.0*) were also preformed with the human or chicken sequences as queries. The sequences were aligned for each family using the Windows version of Clustal X 1.81 [21], [22] and manually inspected to improve incorrectly annotated sequences and remove incomplete sequences. Additional searches using tblastn in both Ensembl and NCBI databases were preformed in order to complete the families (for sequence accession numbers, see Table S1). Phylogenetic trees were constructed as described for the opioid peptide sequences.

### Conserved synteny analysis

Information about the chromosomal location of the genes in each family was collected from the Ensembl database and this information, together with the topology of the phylogenetic trees, were used to construct tables showing conserved synteny, see Table S2. Both the tables and the figure are color-coded according to the chromosomal location of the genes in chicken. The genes in the tables are named following the HGNC name for their human ortholog in order to facilitate comparison between species.

## Results

### Evolution of opioid peptide genes

**Figure 1:**
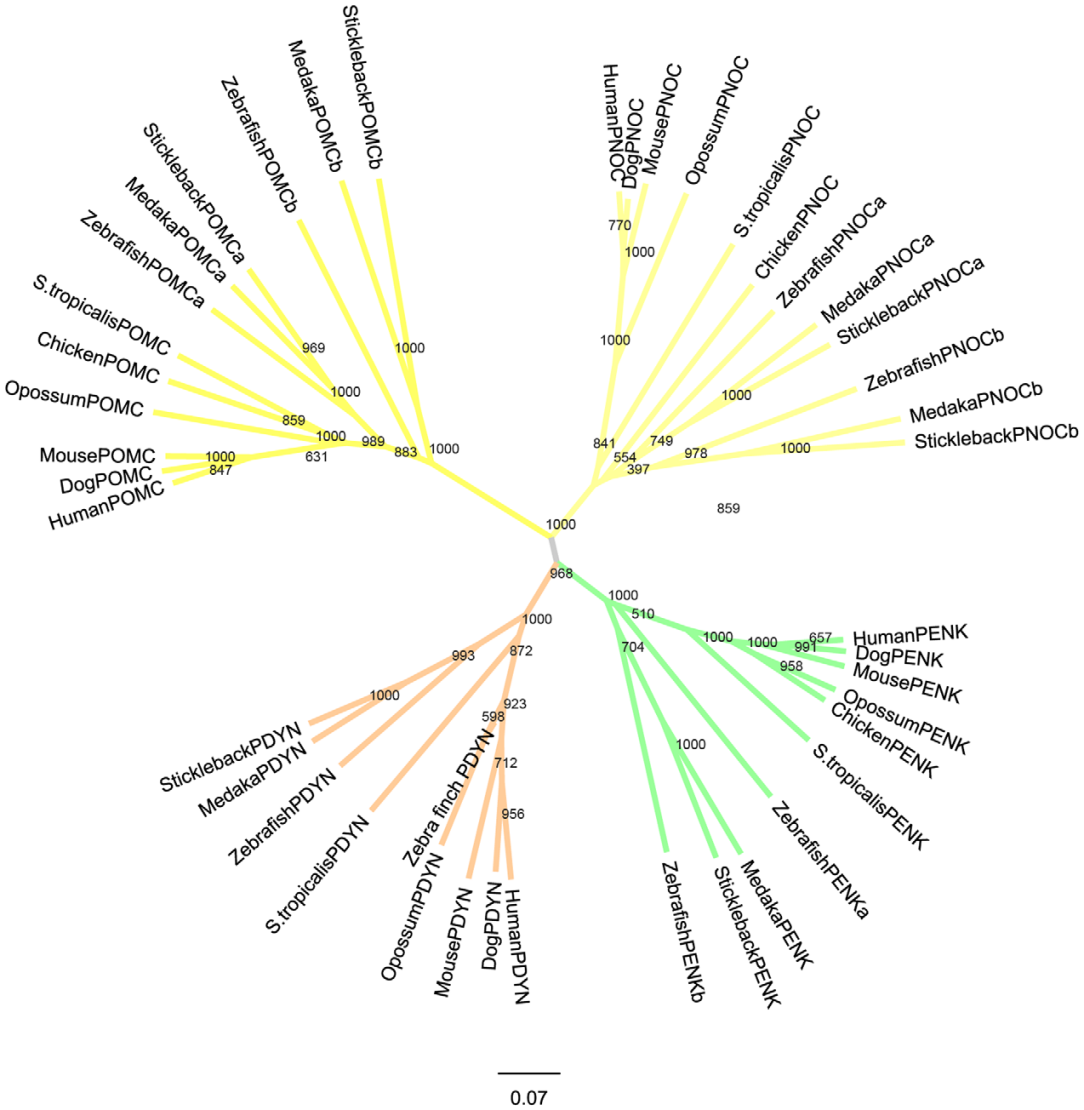
Neighbor-Joining tree for the opioid prepropeptide family. Bootstrap values are shown at the nodes. Abbreviations: PENK preproenkephalin, PDYN preprodynorphin, PNOC preproorphanin and POMC proopioimelanocortin.

Opioid peptide precursor genes were identified in the genome databases for human, mouse, dog, grey short-tailed opossum, chicken, western clawed frog, zebrafish, medaka and stickleback, accession numbers for all sequences are available in Table S1. It was not possible to identify peptide genes in any of the basal chordate genomes we studied, *Ciona intestinalis* and *Branchiostoma floridae* and therefore the phylogenetic tree is displayed unrooted, see Fig. 1. The *POMC* gene in lampreys has been duplicated early in their evolution generating the precursor genes named *POC* and *POM* [24]. We have not included POC and POM protein sequences in our study since their evolution has been thoroughly studied by others and no other opioid precursor sequences have been reported for lampreys and thus they will not shed light on the origin of the four members in gnathostomes. For some vertebrates it was possible to identify the opioid peptide sequences using BLAST searches in their genomes. However, in a few cases only incomplete sequences were found, but also these sequences clustered in the phylogenetic tree together with their orthologues in other species. One such example is PDYN from zebra finch. The stem of the POMC cluster is longer than for the other three genes as a result of the insertion of the melanocortin sequences into this precursor, see Fig. 1.

For several of the teleost opioid peptide genes extra introns had been predicted in the genome database. However the predicted splice sites were usually suboptimal and the intron sequences were in frame in all cases, thereby allowing continuous translation. The first exon in the *PENK* gene in dog was not identified in the database. But we were able to detect the exon almost 4 kb upstream. According to the genome database this exon includes a stop codon instead of the third conserved cystein residue. Due to the high conservation of the rest of the sequence we believe this may be due to a sequencing error. Two extra introns had been predicted for the dog *POMC* gene, but none of the splice sites were optimal and the first intron maintained the reading frame. The second intron was not in frame but with information from EST data we concluded that a nucleotide was missing in the genome sequence.

#### PENK

**Figure 2:**
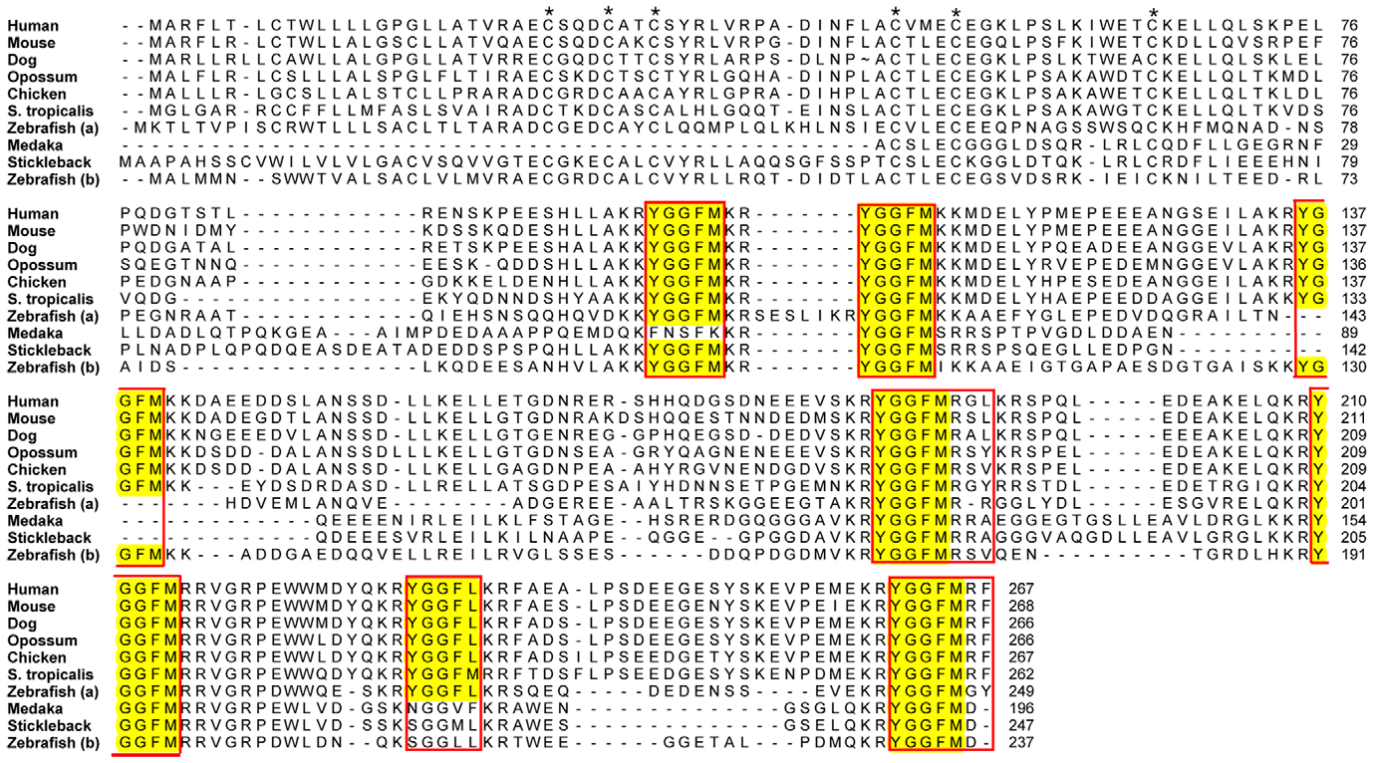
Alignment of the preproenkephalin protein sequences. Conserved cysteines in the N-terminal region are marked with an asterisk and regions corresponding to known mature peptides in either of the sequences are boxed. Enkephalin motifs (YGGFM/L) are shaded.

The PENK precursor (Fig. 2) contains seven enkephalin motifs in tetrapods. Medaka and stickleback have one *PENK* gene and this lacks the third core sequence and has a degenerate sixth core sequence. *PENK* was missing in the medaka genome according to the predictions in the Ensembl genome database, but it was possible to identify the gene through BLAST searches. However, the sequence that corresponds to the first exon and includes the first three cysteines is still missing in the genome database. One interesting feature of the medaka sequence is that it appears as if the first opioid core sequence is degenerated while it is in intact in tetrapods and the other teleosts (Fig. 2). The zebrafish genome is the only sequenced teleost genome with a duplicate of this gene. The gene located on zebrafish chromosome 7 (*PENKa*) has an insertion between the two first core sequences and is missing the third core sequence, while the gene on chromosome 2 (*PENKb*) does not have this insertion and has retained the third core sequence, but has a degenerate sixth core sequence, as described earlier [25], [26]. The zebrafish duplicates presumably resulted from of the teleost-specific whole genome duplication, hence we have named them *PENKa* and *PENKb*.

#### PDYN

**Figure 3:**
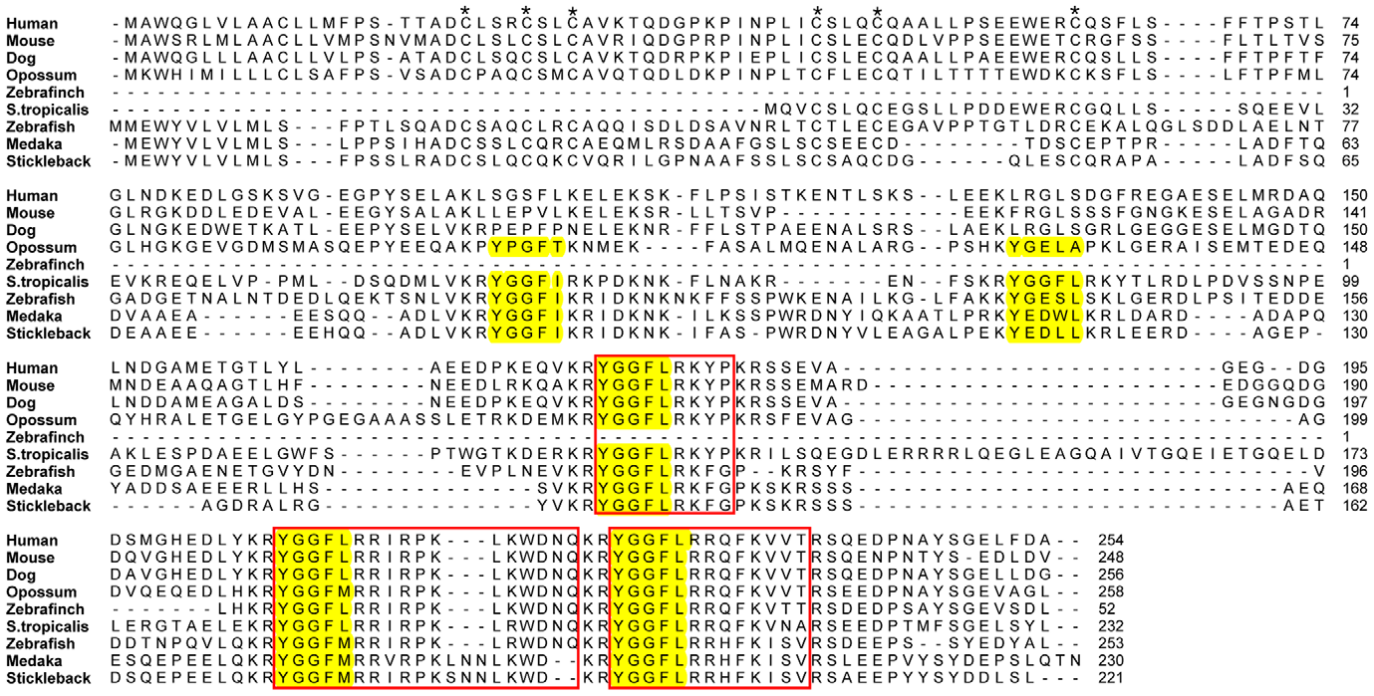
Alignment of the preprodynorphin protein sequences. Conserved cysteines in the N-terminal region are marked with an asterisk and regions corresponding to known mature peptides in either of the sequences are boxed. Dynorphin-like motifs (YGGF…) are shaded.

The *PDYN* gene (Fig. 3) encodes three opioid core motifs in placental mammals and two more in opossum and amphibians. The *PDYN* gene is missing in the chicken genome, but a portion of *PDYN* was detected in the zebra finch genome by using BLAST searches. This fragment includes the sequences for dynorphin A and dynorphin B and is located on chromosome 20, a chromosome that displays conserved synteny to chicken chromosome 20 (data not shown), indicating that the *PDYN* gene once was located there in chicken, on the same chromosome as the gene for the orphanin receptor. Due to the poor assembly of the western clawed frog genome it was only possible to detect a partial dynorphin sequence, but full length sequences have previously been found in other amphibians [27], [28]. The first and second opioid core sequences in *PDYN* have previously been defined as a relic sequences in mammals [25] but they are less degenerate in opossum and intact in amphibians and teleost fishes (Fig. 3). The zebra finch sequence does not cover this part of the alignment, but the core sequence is intact in the green anole lizard (*Anolis carolinensis*) (data not shown). Stickleback and medaka have a deviating second core sequence as described earlier for eel, tilapia and zebrafish [25], [29]. This core sequence is intact in amphibians and less degenerate in opossum than in placental mammals (Fig. 3).

#### PNOC

**Figure 4:**
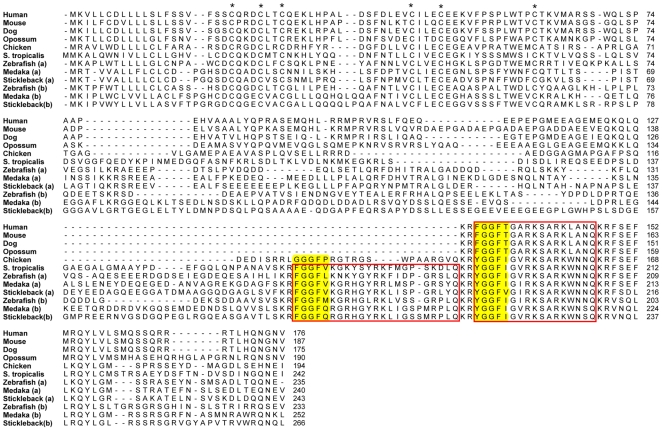
Alignment of the preproorphanin protein sequences. Conserved cysteines in the N-terminal region are marked with an asterisk and regions corresponding to known mature peptides in either of the sequences are boxed. Orphanin/nociceptin-like motifs (XGGF…) are shaded.

The orphanin precursor has a single opioid-like core sequence (FGGFI, as part of a longer peptide called nociceptin) in mammals and one more opioid-like motif (FGGFX) in the non-mammalian species in Fig. 4. We were able to identify the *PNOC* gene in the genome of the western clawed frog. This is the first time, to our knowledge, that an amphibian *PNOC* sequence is described. One interesting feature of frog *PNOC* is the presence of a second nociceptin-like sequence that previously has only been described in ray-finned fishes as sturgeon F peptide or zebrafish nociceptin-like peptide [30], [31]. It is also possible to see remnants of a core sequence at that position in chicken (Fig. 4). All three teleost genomes in this study include duplicates of the *PNOC* gene and this duplication is likely a result of 3R, as supported by the chromosomal locations and phylogenies of neighboring gene families (see Table S2), why we have chosen to designate the two copies *PNOCa* and *PNOCb*, see Fig. 4. There have been reports that the PNOC gene also encodes a nocistatin peptide that is present in mammals but not in zebrafish [30], [32] this peptide contains no opioid core motif and differs greatly among the mammals. Our analysis shows that it is not present in any non-mammal species. Opossum has only four of the six conserved nocistatin amino acids that indicate that the function, if any, has appeared in placental mammals.

#### POMC

**Figure 5:**
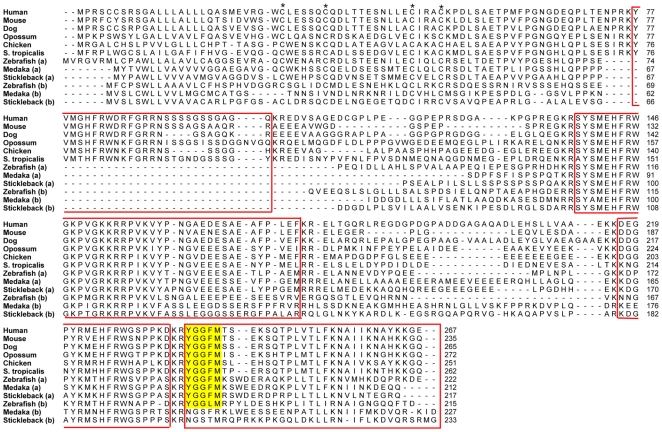
Alignment of the proopioimelanocortin protein sequences. Conserved cysteines in the N-terminal region are marked with an asterisk and regions corresponding to known mature peptides in either of the sequences are boxed. Enkephalin motifs (YGGFM) are shaded.

The POMC precursor contains a single opioid core sequence as part of endorphin and, in addition, 2–4 melanocortin motifs (HFRW). The number of melanocortin peptides varies depending on species. The gene is located next to the *PNOC* gene in chicken and in the teleost fishes, suggesting that these two genes are a result of a local duplication. Duplicates of the zebrafish *POMC* gene have previously been cloned and 3R has been suggested as the origin of the duplicates [33]. Our analysis confirms this and it was also possible to detect duplicates in medaka and stickleback. We have named the duplicates *POMCa* and *POMCb* in accordance with the *PNOC* genes (Fig. 5).

### Phylogenetic analysis of neighboring families

Excluding the opioid prepropeptide genes, 32 adjacent gene families fulfilled the selection criteria that at least three members in the family were located in the vicinity of the opioid peptide genes in chicken. We used *Ciona intestinalis* and *Branchiostoma floridae* to provide relative dating for our phylogenetic trees, because their positions as basal chordates make them suitable as outgroups for the study of the two early vertebrate tetraploidizations [10], [34]. Five of the selected families were excluded, either because they had expanded in a timeframe not interesting for this study or had a multitude of members and/or domains that made alignments unreliable. Some of the families studied here (MYT1, NKAIN, RGS, SRC-B, STMN, XKR and YTHDF) have previously been analyzed by Dreborg et al 2008. However, the present analysis includes sequences from several teleost species and opossum and gives a more complete picture of the evolutionary history of the families. Fifteen of the families in chicken consist of full quartets i. e. all copies generated in the two genome doublings have been retained. The situation in human is almost the same, with fourteen families containing four members. Interestingly, several of the quartets have functions in the nervous system for instance the opioid receptors, DLGAP, EPB41, potassium channels and stathmins. Protein families such as EYA, L3MBTL, MYT and RGS are located in the vicinity of the opioid peptide genes and also have functions in the nervous system. The rest of the families included in the study are: BMP, GATA, LAMA, LPIN, MATN, MYB, MYOM, NCOA, PHACTR, PTPR, RIMS, RSPO, SERINC, TFAP2. The NJ and QP trees for all 27 families are accessible as Fig. S2 and accession numbers for all sequences are available in Table S1.

### Conserved synteny analysis

**Figure 6:**
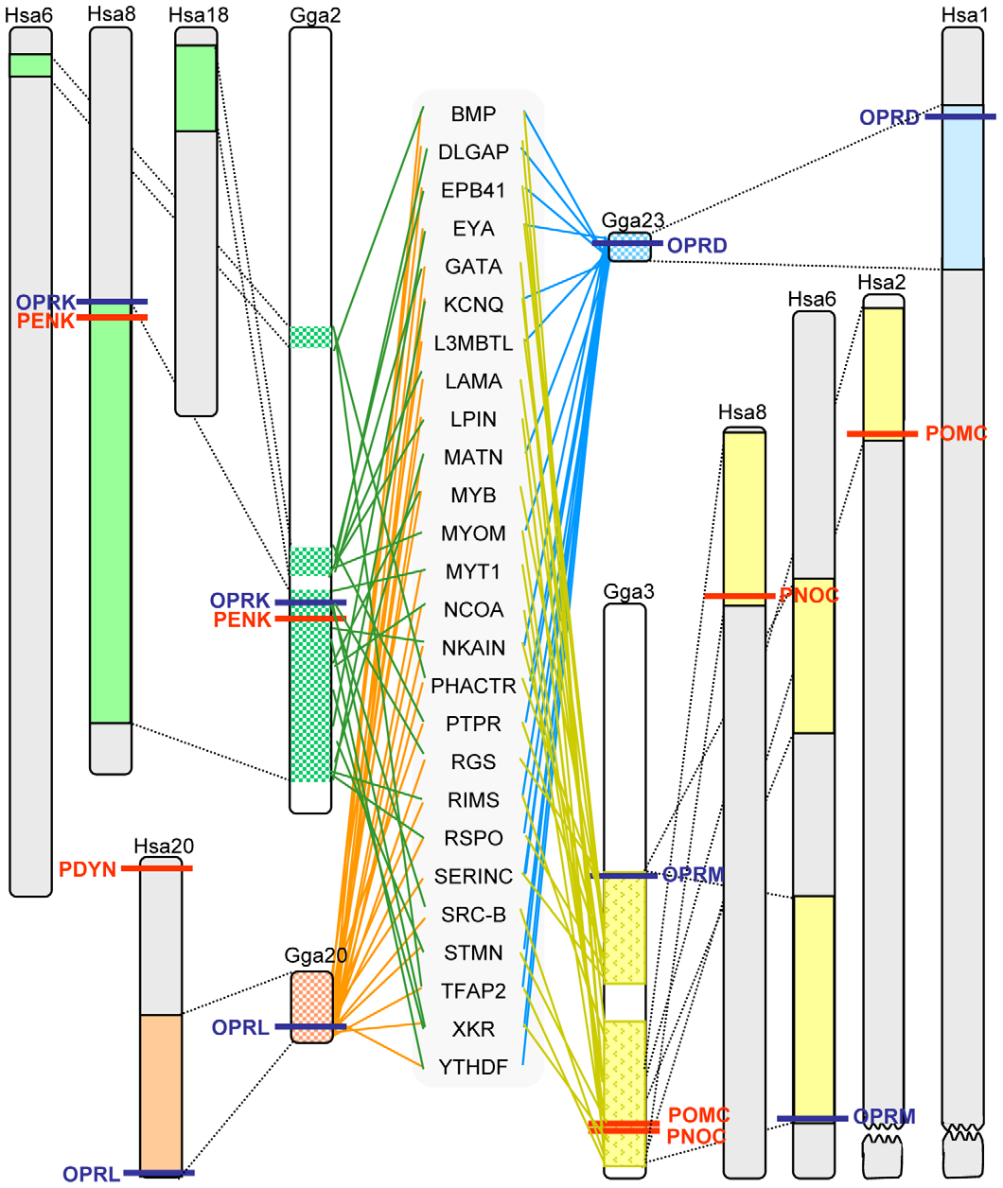
Paralogus regions in chicken and human harbouring the opioid peptides and receptors. Paralogus regions in chicken and human harbouring the opioid peptides and receptors together with several other gene families. For full gene family names see Table S1. Note that several translocations have occurred in human as compared to chicken. Abbreviations: PENK preproenkephalin, PDYN preprodynorphin, PNOC preproorphanin, POMC proopioimelanocortin, OPRM opioid mu receptor, OPRD opioid delta receptor, OPRK opioid kappa receptor and OPRL orphanin receptor.

The four opioid peptide genes are located on three different chromosomes in the human genome, one each on chromosomes 20 (*PDYN*) and 2 (*POMC*) and two on chromosome 8 (*PENK* and *PNOC*), see Fig. 6. The chicken genome displays a four-fold paralogy (i.e., form a quartet of chromosomal regions with a similar setup of gene families) for the chromosomes harboring the opioid receptors, the chromosomes 2, 3, 20 and 23, but it has only three opioid peptides, one on chromosome 2 (*PENK*) and two on chromosome 3 (*POMC* and *PNOC*). It lacks the prodynorphin gene, which in the human genome is located on chromosome 20, a chromosome that shows conserved synteny with chicken chromosome 20 (see Table S2 and Fig. 6.). Although *POMC* and *PNOC* are situated close together on chicken chromosome 3, they are on separate chromosome in the human genome, 2 and 8 respective as a result of a translocation in the ancestor of placental mammals. The location of *POMC* and *PNOC* together seems to be the ancestral configuration strengthened by the fact that opossum has the two genes together like in chicken, as do the teleost fishes (see Table S2). Interestingly *PNOC* and *PENK* are located on the same chromosome in human (on 8p and 8q respectively) albeit far apart in regions that display conserved synteny with two different chicken chromosomes. The fusion leading to the present human chromosome 8 seems to have taken place in early primate evolution. Importantly, figure 6 shows that the opioid receptor genes are located in the same genomic regions as the peptide precursor genes. It seems like an intrachromosomal rearrangement has occurred in human chromosome 20 resulting in *PDYN* and the gene for nociceptin receptor (*OPRL*) being located in opposite ends of the chromosome. This is also the case in the genome of the chimpanzee (*Pan troglodytes*), but in the genomes of orangutan (*Pongo pygmaeus abelii*) and the rhesus monkey (*Macaca mulatta*) as well as in other mammals the distance between the genes is smaller (data not shown). This suggests that the distance increased in the Homininae subfamily of the hominids.

## Discussion

**Figure 7:**
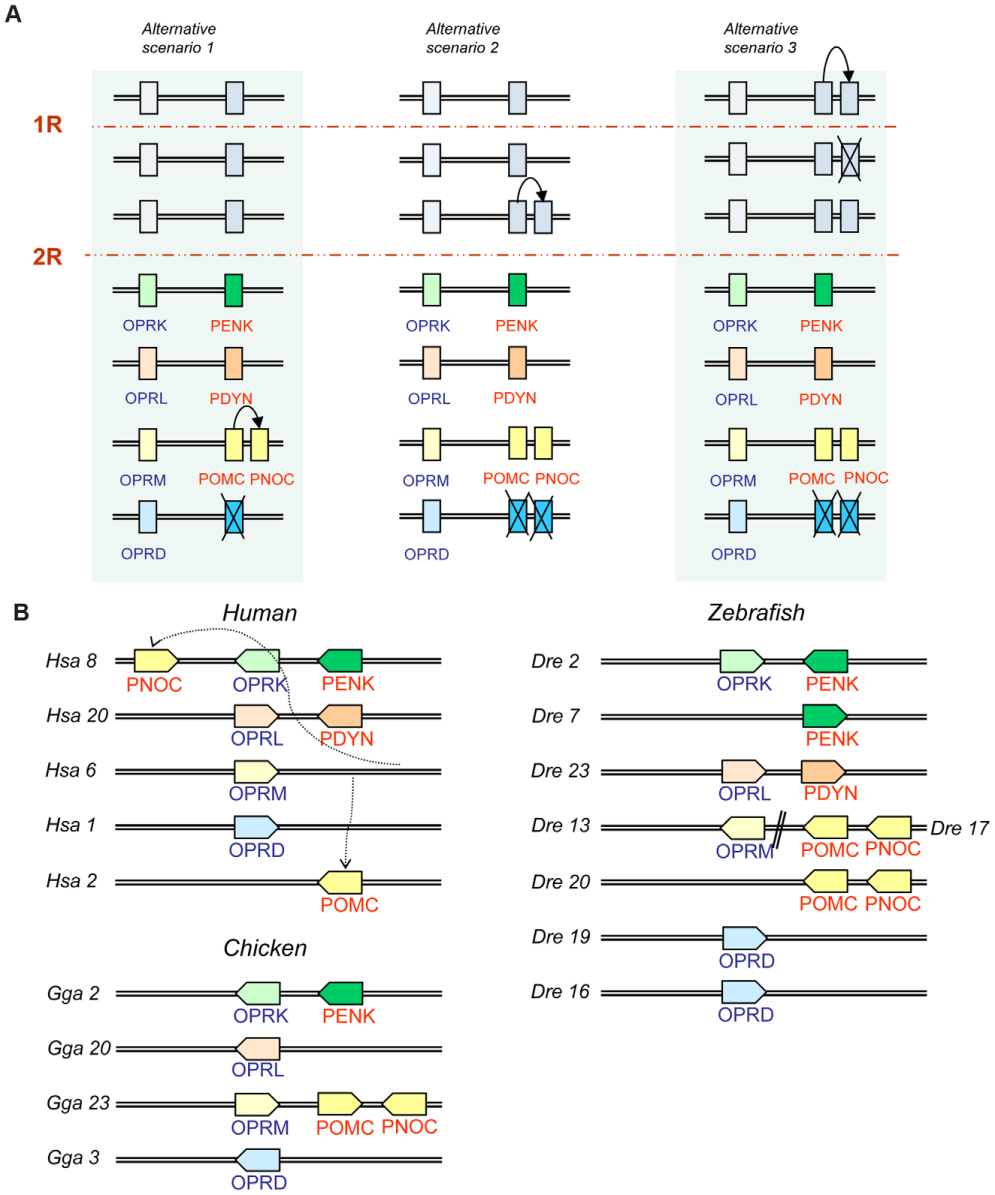
Proposed evolutionary history and the present locations for the opioid peptide and receptor genes. A: Proposed evolutionary history for the opioid peptides and receptors by genome and local duplications. The timing of the duplication that generated PNOC and POMC from their common ancestor is still unresolved and three different scenarios are presented. B: The present locations of the opioid peptide and receptor genes in human, chicken and zebrafish. Several gene families in these chromosomal regions have a similar evolutionary history, see Fig. 6 and Table S2. Abbreviations: PENK preproenkephalin, PDYN preprodynorphin, PNOC preproorphanin and POMC proopioimelanocortin, OPRM opioid mu receptor, OPRD opioid delta receptor, OPRK opioid kappa receptor and OPRL orphanin receptor.

By taking a comparative approach utilizing both sequence information and the chromosomal locations of the gene family members (relative to their neighboring genes) we propose that the expansion of the opioid peptide gene family is a result of the two whole genome duplications together with one local duplication, see Fig. 7A. Furthermore, we propose that two of the peptide genes were duplicated concomitantly with opioid receptor genes located adjacently in the same chromosomal regions. Twenty-seven gene families neighboring the opioid peptide genes present phylogenetic topologies and species distributions that are consistent with duplications around the time of 2R. Their positions in chromosomal regions sharing members of the same gene families support duplications of large blocks, most likely the chromosome duplications that took place in 2R.

Although four chromosomes are clearly involved as shown by the many adjacent gene families, the peptide genes are located on only three of these. The fourth peptide gene most likely arose by a local duplication resulting in *PNOC* and *POMC*. These genes are located close together in the chicken and opossum genomes as well as the teleost genomes that we have studied. The time point for this local duplication event is difficult to pinpoint and we outline three possible scenarios in Fig. 7A. As there is no remnant of melanocortin sequences in *PNOC*, it seems like the duplication took place before the incorporation of melanocortin sequences in *POMC*. Lampreys have two POMC sequences, called *POC* and *POM*, the two lamprey genes seem to be the result of a lineage-specific duplication that resulted in highly divergent sequences compared to other species [24]. We have not included these sequences in our analysis but the existence of *POC* and *POM* in cyclostomes provides relative dating of the duplication that generated PNOC and POMC to a time point before the split of cyclostomes from other chordates. This implies that scenario 1 in figure 7A is unlikely, assuming that lampreys diverged before the second round of duplication. However, it is still unclear if the cyclostomes experienced one or two tetraploidizations [35], [36] so although we can say that *POMC* arose before the lamprey-gnathostome split, it is still not possible to say definitively if this happened before the second genome doubling. It is interesting that *PNOC* and *POMC* are the result of a local duplication because they are the most divergent sequences in the quartet of opioid peptide precursors. If this local duplication is the most recent duplication event, this implies that both *PNOC* and *POMC* have had a higher rate of sequence divergence and rearrangement causing their appearance. Of the two other scenarios in Fig. 7A, scenario 2 is more parsimonious as it involves fewer losses of gene duplicates, but as losses are quite common also scenario 3 is possible.

Figure 7B shows the present location of the opioid peptide and receptor genes in the genomes of human, chicken and zebrafish. Among these three species, chicken seems to have greatest resemblance to the deduced ancestral configuration of the genes. In human, *PNOC* is translocated from chromosome 6 to chromosome 8, a chromosome that already harbored the genes for *PENK* and opioid kappa receptor, thereby initially giving an impression of local duplication on this chromosome. Three of the prepropeptide genes have duplicates in zebrafish, most likely generated in 3R. In contrast, only one receptor (delta, *OPRD*) is duplicated in zebrafish. Additional receptor duplicates have survived in other teleost fish species [7].

After the peptide gene family expanded in 2R, each prepropeptide has had its own evolutionary history and presently there are several differences between the precursors in the different classes of vertebrates. By studying many species it is possible to relatively date events such as degeneration or losses of individual peptides within the precursors or even entire genes. Our identification of the *PNOC* gene in an amphibian gives a relative date for another interesting event, because the presence of a second nociceptin-like peptide in the frog precursor strengthens the hypothesis that this is an ancestral feature that has been lost in amniotes. It seems like chicken still has a relic of this sequence (Fig. 4). The presence in chicken indicates a gradual degeneration of the second nociceptin peptide before loss in mammals rather than deletion by unequal crossing-over as has previously been suggested [31].

The *PDYN* gene in opossum contains two degenerate opioid core sequences where it is not possible to see any remnant of the enkephalin motif in placental mammals (Fig. 3). The situation is the same for the *PDYN* gene in wallaby (data not shown). The loss of the *PDYN* gene in chicken is unexpected since it is present in mammals, fishes and in the lizard *Anolis carolinensis*. The *PDYN* gene is present as a fragment in the zebra finch genome but due to poor quality of the sequence in that region it is not possible to say if the gene is functional or in a state of degeneration. The newly sequenced turkey genome (*Meleagris gallopavo*) revealed a full-length *PDYN* gene. A comparison of the local genomic environment in chicken and turkey (data not shown) strongly indicates that the gene is deleted in chicken.

All teleost fishes have had one additional genome duplication (3R) and for some of the opioid peptide genes the duplicates have survived. The zebrafish has two genes for *PENK* which have retained different elements. All three teleost species we have studied show duplicates of *PNOC* and *POMC* while neither of the species has retained more than one copy of *PDYN*. The 3R duplicates have diverged from each other and for some species a few of the opioid peptides show degeneration. There also exist duplicates of the peptide genes in species that we have not included in our analysis because their genomes have not been sequenced and/or assembled. Duplicates and in some cases triplicates of the *POMC* gene exist in teleost species that have undergone independent tetraploidizations, namely salmonids, carps and sturgeons [37], as well as the tetraploid frog Xenopus laevis [38], while others may be the result of other duplication mechanisms such as the triplicates in barfin flounder [37].

Our observation that the peptide and receptor genes were once on the same chromosomes (and still are in some species, although quite far apart) suggests that there may once have been a functional reason for the genetic linkage, such as coexpression and/or coevolution. The phenomenon of receptors and their ligands being located on the same chromosomes have been observed earlier in the cases of *FGF/FGFR*, *TNFSF/TNFRSF*, interleukin receptors with antagonists and *MST1/MST1R* [39], [40], [41], [42]. However, a systematic study using several gene families in human concluded that it is not possible to see evidence for selection of receptor-ligand clusters [43]. Nevertheless, it was possible to see that in the human genome (albeit not in the highly rearranged mouse genome) receptors and their ligands are located on the same chromosomes more often than expected by chance [43].

After peptides and receptors were duplicated, resulting in 3–4 peptide precursors and 4 receptors, new binding preferences may have started to emerge that did not correlate with chromosomal location. One example is dynorphin in human that has highest affinity for the kappa receptor [8] but is located on the same chromosome as the orphanin receptor. Different evolutionary lineages may have evolved different preferred receptor-ligand functional partnership. The orphanin peptide in humans shows no relevant binding to any other receptor than the orphanin receptor, but the sturgeon orphanin peptide (also called sturgeon Y peptide, Tyr^161^–Pro^177^) can bind to all rat opioid receptors [31]. In this context it is interesting that the teleost, chicken and western clawed frog orphanin peptide sequences show high identity to the heptadecapeptide sturgeon Y peptide with 14–16 identical amino acids, as compared to the mammalian sequences where only 11 of 17 amino acids are identical. Strikingly, non-mammalian orphanin starts with a Y and position 14 is a tryptophan, and these positions are identical to the corresponding residues in dynorphin A. The orphanin peptide from non-mammalian species is more similar to the classical opioid peptides than the mammalian orphanin. This shows that setting the mammalian peptides as standard can complicate the identification of peptides.

As a by-product, the present analyses have also resolved the evolution in vertebrates of many neighbouring gene families that, consequently, also expanded in early vertebrate evolution. Some of these are of great neurobiological significance, for instance DLGAP1, potassium channels and MYT1. Thus, both these and the opioid receptors and peptides may have made important contributions to the elaboration and diversification of the nervous system in vertebrates.

## Supporting Information

Table S1 List of accession numbers for all the sequences used in the phylogenetic analyzis.
(1.53 MB XLS)

Table S2 The table shows the analyzed gene families and the chromosomal location of the genes. All human gene names in the figure are approved HGNC symbols and the genes in the other species have been given the names of their human orthologs. The tables are color-coded based on the chicken chromosomes (chromosome 2, green; chromosome 20, orange; chromosome 23, blue; and chromosome 3, yellow). An asterisk after the family name indicates that the NJ and QP trees display different topologies (see Fig. S1).
(0.15 MB XLS)

Figure S1 Final alignment for the opioid peptides.
(0.08 MB PDF)

Figure S2 Neighbor-joining and quartet-puzzling maximum likelihood trees for all families included in the study.
(0.96 MB PDF)
