# Supplementary material for: Concomitant Duplications of Opioid Peptide and Receptor Genes before the Origin of Jawed Vertebrates
Source: PLoS One. 2010 May 6;5(5):e10512. doi: 10.1371/journal.pone.0010512 (PMC2865548; doi:10.1371/journal.pone.0010512)
Supplement: Figure S1 — Final alignment for the opioid peptides. (0.08 MB PDF) [file pone.0010512.s003.pdf]

```
                                  10        20        30        40
                         ....|....|....|....|....|....|....|....|
HumanPENK                --MARFLT-LCTWLLLGPGLLATVRAECSQDCATCSYRL
MousePENK                --MARFLR-LCTWLLALGSCLLATVQAECSQDCAKCSYRL
DogPENK                  --MARLLRLLCAWLLALGPGLLATVRRECGQDCTTCSYRL
OpossumPENK              --MALFLR-LCSLLLALSPGLFLTIRAECSKDCTSCTYRL
ChickenPENK              --MALLLR-LGCSLLALSTCLLPRARADCGRDCAACAYRL
S.tropicalisPENK         --MGLGAR-RCCFFLLMFASLSVAIRADCTKDCASCALHL
ZebrafishPENKa           -MKTLTVPISCRWTLLLSACLTLTARADCGEDCAYCLQQM
MedakaPENK               ----------------------------------------
SticklebackPENK          MAAPAHSSCVWILVLVLGACVSQVVGTECGKECALCVYRL
ZebrafishPENKb           --MALMMN--SWWTVALSACLVLMVRAECGRDCALCVYRL
HumanPDYN                ------MAWQGLVLAACLLMFPS-TTADCLSRCSLCAVKT
MousePDYN                ------MAWSRLMLAACLLVMPSNVMADCLSLCSLCAVRI
DogPDYN                  ------MAWQGLLLAACLLVLPS-ATADCLSQCSLCAVKT
OpossumPDYN              ------MKWHIMILLLCLSAFPS-VSADCPAQCSMCAVQT
Zebra finchPDYN          ----------------------------------------
S.tropicalisPDYN         ----------------------------------------
ZebrafishPDYN            -----MMEWYVLVLMLS---FPTLSQADCSAQCLRCAQQI
MedakaPDYN               ------MEWYVLVLMLS---LPPSIHADCSSLCQRCAEQM
SticklebackPDYN          ------MEWYVLVLMLS---FPSSLRADCSLQCQKCVQRI
HumanPNOC                ------MKVLLCDLLLLSLFSSV--FSSCQRDCLTCQEKL
MousePNOC                ------MKILFCDVLLLSLLSSV--FSSCPRDCLTCQEKL
DogPNOC                  ------MKILLCDLLLLSLFSSV--SGSCQKDCLTCREKL
OpossumPNOC              ------MKTLLCDLLLLGLFSNV--FSDCQKDCLTCREKL
ChickenPNOC              ------MRAVLWDLLLLCLFARA--RSDCRGDCLRCDRHF
S.tropicalisPNOC         -----MMKALQWNIVLLCLLGHV--LCDCQKDCMTCNKHL
ZebrafishPNOCa           ------MKAPLWTLLLLGLCNPA--WCDCQKDCLFCSQKL
MedakaPNOCa              ------MRT-VVALLFLCLCD--PGHSDCQADCLSCNNIL
SticklebackPNOCa         ------MKT-VVALLLLCLCD--PGQSDCQADCVSCSNML
ZebrafishPNOCb           ------MKTPFWTLLLLCLCASS--HSDCQGDCLTCGLIL
MedakaPNOCb              ------MKIPLWCLVVLLACLFSPGHCDCQGECVACGLLL
SticklebackPNOCb         ------MKIPVWYLLVLLASVFTPGRGDCQGECVACGALL
HumanPOMC                ----MPRSCCSRSGALLLALLLQASMEVRG-WCLESSQCQ
MousePOMC                PRFCYSRSGALLLALLLQTSIDVWS-WCLESSQCQDLTT-
DogPOMC                  ----MPRSCCSRPGALLLALLLQASVEVSG-WCLESSQCQ
OpossumPOMC              ----MPKPSWSYLGALLVAVLFQASVEVHG-WCLQASNCR
ChickenPOMC              ----MRGALCHSLPVVLGLLLCHPT-TASG-PCWENSKCQ
S.tropicalisPOMC         ----MFRPLWGCSLAILGAFIFHVG-EVQG-QCWESSRCA
ZebrafishPOMCa           --MVRGVRMLCPAWLLALAVLCAGGSEVRA-QCWENARCR
MedakaPOMCa              ---------MYTVWLLVAVVVVGGAEGAVG-QCWKHSSCQ
SticklebackPOMCa         ---------MYPAWLLVVVAVMGVAGGDVS-LCWEHPSCQ
ZebrafishPOMCb           --------MFCPSWLLAAAVLCFHSPHVDGGRCSGLIDCM
MedakaPOMCb              --------MVSLCWLLVVLMGCMCATGS-----TNNSIVN
SticklebackPOMCb         --------MVSLSWLLVVAVACARLPGFGS-ACLDRSQCD
```

```
                                      50        60        70        80
                    ....|....|....|....|....|....|....|....|
HumanPENK           VRPA---DINFLACVMECEGKLPSLKIWETCKELLQLSKP
MousePENK           VRPG---DINFLACTLECEGQLPSFKIWETCKDLLQVSRP
DogPENK             ARPS---DLNP-ACTLECEGKLPSLKTWEACKELLQLSKL
OpossumPENK         GQHA---DINPLACTLECEGKLPSAKAWDTCKELLQLTKM
ChickenPENK         GPRA---DIHPLACTLECEGKLPSAKAWETCKELLQLTKL
S.tropicalisPENK    GQQT---EINSLACTLECEGKLPSAKAWGTCKELLQLTKV
ZebrafishPENKa      PLQLK--HLNSIECVLECEEQPNAGSSWSQCKHFMQNAD-
MedakaPENK          -------------ACSLECGGGLDSQR-LRLCQDFLLGEGR
SticklebackPENK     LAQQS--GFSSPTCSLECKGGLDTQK-LRLCRDFLIEEEH
ZebrafishPENKb      LRQT---DIDTLACTLECEGSVDSRK-IEICKNILTEED-
HumanPDYN           QDGPK--PINPLICSLQCQAALLPSEEWERCQSFLS----
MousePDYN           QDGPR--PINPLICSLECQDLVPPSEEWETCRGFSS----
DogPDYN             QDRPK--PIEPLICSLECQAALLPAEEWERCQSLLS----
OpossumPDYN         QDLDK--PINPLTCFLECQTILTTTTEWDKCKSFLS----
Zebra finchPDYN     ----------------------------------------
S.tropicalisPDYN    ----------MQVCSLQCEGSLLPDDEWERCGQLLS----
ZebrafishPDYN       SDLDS--AVNRLTCTLECEGAVPPTGTLDRCEKALQGLSD
MedakaPDYN          LRSDA--AFGSLSCSEECD--------TDSCEPTPR----
SticklebackPDYN     LGPNA--AFSSLSCSAQCDG------QLESCQRAPA----
HumanPNOC           HPAL--DSFDLEVCILECEEKVFPSPLWTPCTKVMARSS-
MousePNOC           HPAP--DSFNLKTCILQCEEKVFPRPLWTVCTKVMASGS-
DogPNOC             RPAL--DSFNLEVCILECEGKVFSSPLWTPCTKVMARSS-
OpossumPNOC         HPFL--DNFSVDECITDCEGQVFLSPFWAPCTKAMNKGP-
ChickenPNOC         YR----DGFDLLVCILECEGEAVPRATWEMCATSIRS--A
S.tropicalisPNOC    YQQ---DNFNTLLCIVECEGKIYSSSMWSICKTVLVQSS-
ZebrafishPNOCa      PNE---YAFNNLVCLVECHGKLSPGDTWEMCRRTIVEQKP
MedakaPNOCa         SKH---LSFDPTVCLIECEGNLSPSFYWNFCQTTLTS---
SticklebackPNOCa    PRQ---LSFNPMVCLTECEADVSPNFFWDFCGKVLSS---
ZebrafishPNOCb      PEH---QAFNTLVCILECEAQASPALTWDLCYQAAGLKH-
MedakaPNOCb         QQHQLQQAFNTIVCLLECEGHASSSLTWEVCKRALKH---
SticklebackPNOCb    QQQQLPQAFNALVCFLECEGGVSSSFTWEVCQRAMKLSR-
HumanPOMC           DLTT---ESNLLECIRACKPDLSAETPMFPGNGDEQPLTE
MousePOMC           --ESNLLACIRACKLDLSLETPVFPGNGDEQPLTENPRKY
DogPOMC             DLTT---ESNLLACIRACKPDLSAETPVLPGNGDEQPLAE
OpossumPOMC         DSKA---EDGLVECIKSCKMDLSAESPVFPGNGQYEPLSE
ChickenPOMC         DLAT---EAGVLACAKACHAELSAEAPVYPGNGHLQPLSE
S.tropicalisPOMC    DLSS---EDGVLECIKACKMDLSAESPVFPGNGHLQPLSE
ZebrafishPOMCa      DLST---EENILECIQLCRSELTDETPVYPGESHLQPPSE
MedakaPOMCa         ELDS---ESSMTECLQLCRSDLTAETPLIPGSSHLQPPPP
SticklebackPOMCa    DVNS---ETSMMECVELCRSDLTAEAPVVPGAAHLQPPPP
ZebrafishPOMCb      DLES---NEHKLQCLRKCRSDQESSRNIRVSSSEHQSSEE
MedakaPOMCb         DLNR---KNRILDCVHLCMSGIKPESPDLG--GSVLPPKN
SticklebackPOMCb    ELNG---EGETQDCIRRCVSAVQPEAPRLG--ALALEVSG
```

```
                                  90        100       110       120
                        ....|....|....|....|....|....|....|....|
HumanPENK               ELPQDGTSTL--------------RENSKPEESHLLAKRY
MousePENK               EFPWDNIDMY--------------KDSSKQDESHLLAKKY
DogPENK                 ELPQDGATAL--------------RETSKPEESHALAKKY
OpossumPENK             DLSQEGTNNQ--------------EESK-QDDSHLLAKKY
ChickenPENK             DLPEDGNAAP--------------GDKKELDENHLLAKKY
S.tropicalisPENK        DSVQDG------------------EKYQDNNDSHYAAKKY
ZebrafishPENKa          NSPEGNRAAT--------------QIEHSNSQQHQVDKKY
MedakaPENK              NFLLDADLQTPQKGEA-----AIMPDEDAAAPPQEMDQKF
SticklebackPENK         NIPLNADPLQPQDQEASDE--ATADEDDSPSPQHLLAKKY
ZebrafishPENKb          RLAIDS------------------LKQDEESANHVLAKKY
HumanPDYN               FFTPSTLGLN--------------DKEDLGSKSVG-EGPY
MousePDYN               FLTLTVSGLR--------------GKDDLEDEVAL-EEGY
DogPDYN                 FFTPFTFGLN--------------GKEDWETKATL-EEPY
OpossumPDYN             LFTPFMLGLH--------------GKGEVGDMSMASQEPY
Zebra finchPDYN         ----------------------------------------
S.tropicalisPDYN        -SQEEVLEVK--------------REQELVP-PML--DSQ
ZebrafishPDYN           DLAELNTGAD--------------GETNALNTDEDLQEKT
MedakaPDYN              -LADFTQDVA--------------AEA------EESQQ--
SticklebackPDYN         -LADFSQDEA--------------AEE------EEHQQ--
HumanPNOC               WQLSPAAP----------------------------EHVA
MousePNOC               GQLSPADP----------------------------ELVS
DogPNOC                 WQLSPAAP----------------------------EHVA
OpossumPNOC             WQLSLASK----------------------------DEAM
ChickenPNOC             PRLGATGAG------------------------VLGAMEP
S.tropicalisPNOC        LQLSVDSVGG-------------FQEDYKPINMEDGQFAS
ZebrafishPNOCa          KALLSVEGSI-------------LKRAEEEP-----DTSL
MedakaPNOCa             -PISTINSSI-------------KKRSREEA-----EALF
SticklebackPNOCa        -PISTLAGTI-------------QKRSREEV-----EALF
ZebrafishPNOCb          LPLPLQDEET-------------SKRSD------------
MedakaPNOCb             QETLQEGGAF-------------LKRGGEQLKLTSEDLNS
SticklebackPNOCb        RPSLPGGGAV-------------LGRTGEGLELTYLDMNP
HumanPOMC               NPRKYVMGHFR----------WDRFGRRNSSSSGSSGAG-
MousePOMC               VMGHFR----------WDRFGPRNSSSAGSAAQR----RA
DogPOMC                 NPRKYVMGHFR----------WDRFGRRN----GSAGQK-
OpossumPOMC             NIRKYVMSHFR----------WNKFGRRNISSGSISSDGG
ChickenPOMC             SIRKYVMSHFR----------WNKFGRRNS-----SSGG-
S.tropicalisPOMC        SIRKYVMTHFR----------WNKFGRRNSTGNDGSSSG-
ZebrafishPOMCa          ----------------------------------------
MedakaPOMCa             ----------------------------------------
SticklebackPOMCa        ----------------------------------------
ZebrafishPOMCb          ----------------------------------------
MedakaPOMCb             ----------------------------------------
SticklebackPOMCb        ----------------------------------------
```

```
                                     130       140       150       160
                          ....|....|....|....|....|....|....|....|
HumanPENK                 GGFMKR-------YGGFMKKMDELY-----PMEPEEEANG
MousePENK                 GGFMKR-------YGGFMKKMDELY-----PMEPEEEANG
DogPENK                   GGFMKR-------YGGFMKKMDELYP-----QEADEEANG
OpossumPENK               GGFMKR-------YGGFMKKMDELY-----RVEPEDEMNG
ChickenPENK               GGFMKR-------YGGFMKKMDELY-----HPESEDEANG
S.tropicalisPENK          GGFMKR-------YGGFMKKMDELY-----HAEPEEDDAG
ZebrafishPENKa            GGFMKRSESLIKRYGGFMKKAAEFY-----GLEPEDVDQG
MedakaPENK                NSFKKR-------YGGFMSRRSPTP-----VGDLDDAEN-
SticklebackPENK           GGFMKR-------YGGFMSRRSPSQ-----EGLLEDPGN-
ZebrafishPENKb            GGFMKR-------YGGFMIKKAAEI-----GTGAPAESDG
HumanPDYN                 SELAKL-------SGSFLKELEKSK-FLPSISTKENTLSK
MousePDYN                 SALAKL-------LEPVLKELEKSR-LLTSVP--------
DogPDYN                   SELVKR-------PEPFPNELEKNR-FFLSTPAEENALSR
OpossumPDYN               EEQAKP-------YPGFTKNMEK----FASALMQENALAR
Zebra finchPDYN           ----------------------------------------
S.tropicalisPDYN          DMLVKR-------YGGFIRKPDKNK-FLNAKR-------E
ZebrafishPDYN             SNLVKR-------YGGFIKRIDKNKNKFFSSPWKENAILK
MedakaPDYN                ADLVKR-------YGGFIKRIDKNK-ILKSSPWRDNYIQK
SticklebackPDYN           ADLVKR-------YGGFIKRIDKNK-IFAS-PWRDNYVLE
HumanPNOC                 AALYQPR---ASEMQHL-RRMPRVR------SLFQEQ---
MousePNOC                 AALYQPK---ASEMQHL-KRMPRVR------SLVQVRDAE
DogPNOC                   TVLHQPS---TSEIQ-L-KRMPRIR------SLIQAQ---
OpossumPNOC               ASVYQPQ---VMEVQGLSQMEPKNR------VSRVRSLYQ
ChickenPNOC               AEAVASP---LQVSELLRRRD-------------------
S.tropicalisPNOC          NFKRLSD---LTKLVDLNKMKEGKR------LS-------
ZebrafishPNOCa            PVDQDD----------EQLSETLQR------FDHITRALG
MedakaPNOCa               PKEDEQ------MEEDLLLPLALQR------FDHVTRALG
SticklebackPNOCa          SEEEEEE---EPLKEGLLLPFAPQR------YNPMTRALG
ZebrafishPNOCb            DEAEPVA---TVSIENDNGVEYTEA------LERFRHALQ
MedakaPNOCb               DSKLLQP---ADRFQDQDLDDADLR------SVQYDSSLQ
SticklebackPNOCb          DSQLPQS---AAAAE-QDGAPFEQR------SAPYDSSLL
HumanPOMC                 ---QKREDVSAGEDCGPLPE---GGPEPRSDGA----KPG
MousePOMC                 EEEAVWGD----------GSPEP-----------SPREGK
DogPOMC                   ---REEEEVAAGGGRAPLPA---GGPGPRGDGG----ELG
OpossumPOMC               NVGQKRQELMQGDFLDLPPPGVWGEDEEMQEGLPLIRKAR
ChickenPOMC               ---HKREEVAG------LALPAASPHHPAGEEEDG-EGLE
S.tropicalisPOMC          ---YKREDISNYPVFNLFPVSDNMEQNAQGDNMEG-EPLD
ZebrafishPOMCa            -------------PEQIDLLAHLSPVALAAPEQIEPESGP
MedakaPOMCa               -------------------------------SDPFSFISP
SticklebackPOMCa          ----------------------PSEALPILSLLSSPSSPS
ZebrafishPOMCb            ---------QVEEQSLSLGLLLSALSPDSIELQNPTAEAP
MedakaPOMCb               -----------------IDDGDLLLSIFLATLAAQDKASE
SticklebackPOMCb          -------------DDGDLPLSVILAALVSENKIPESDLRG
```

```
                             170       180       190       200
                    ....|....|....|....|....|....|....|....|
HumanPENK           SEILAKRYGGFMKKDAEEDDSLANSSD-LLKELLETGDNR
MousePENK           GEILAKRYGGFMKKDADEGDTLANSSD-LLKELLGTGDNR
DogPENK             GEVLAKRYGGFMKKNGEEEDVLANSSD-LLKELLGTGENR
OpossumPENK         GEVLAKRYGGFMKKDSDD-DALANSSDLLLKELLGTGDNS
ChickenPENK         GEILAKRYGGFMKKDSDD-DALANSSD-LLKELLGAGDNP
S.tropicalisPENK    GEILAKKYGGFMKK---EYDSDRDASD-LLRELLATSGDP
ZebrafishPENKa      RAILTN----------HDVEMLANQVE--------ADGER
MedakaPENK          ----------------------QEEEENIRLEILKLFSTA
SticklebackPENK     ----------------------QDEEESVRLEILKILNAA
ZebrafishPENKb      TGAISKKYGGFMKK---ADDGAEDQQVELLREILRVGLSS
HumanPDYN           S--LEEKLRGLSDGFREGAESELMRDAQLNDGAMETGTLY
MousePDYN           ----EEKFRGLSSSFGNGKESELAGADRMNDEAAQAGTLH
DogPDYN             S--LAEKLRGLSGRLGEGGESELMGDTQLNDDAMEAGALD
OpossumPDYN         G--PSHKYGELAPKLGERAISEMTEDEQQYHRALETGELG
Zebra finchPDYN     ----------------------------------------
S.tropicalisPDYN    N--FSKRYGGFLRKYTLRDLPDVSSNPEAKLESPDAEELG
ZebrafishPDYN       G-LFAKKYGESLSKLGERDLPSITEDDEGEDMGAENETGV
MedakaPDYN          AATLPRKYEDWLKRLDARD----ADAPQYADDSAEEERLL
SticklebackPDYN     AGALPEKYEDLLKRLEERD----AGEP-------AGDRAL
HumanPNOC           --------EEPEPGMEEAGEMEQKQLQ-------------
MousePNOC           PGADAEPGADAEPGADDAEEVEQKQLQ-------------
DogPNOC             --------EGTEPGMDEAGEIEQKQLQ-------------
OpossumPNOC         AQ-----EEEAAEGLGEAGEEMQKKLQ-------------
ChickenPNOC         -----------AEDGGAGMAPGAFPSQ-------------
S.tropicalisPNOC    ----------DISDLIREQSEEDPSLDGAEGALGMAAYPD
ZebrafishPNOCa      ADDQD-----RQLSKKY------KFLQVQS-AQESEEERD
MedakaPNOCa         INEKDLGDESNQLNTAY------KSLNALSLENEYDEQEG
SticklebackPNOCa    LDER------HQLNTAH-NAPNAPSLEDEYEEDAGQEEGG
ZebrafishPNOCb      PSEELEKLTAS-----YDPDLDPRTQEDQDDLG-------
MedakaPNOCb         ESSE-EVQS-----------LDPRLEGKEETQRDDRDVKG
SticklebackPNOCb    ESSEGEEVEEEGEEGPLGWHPSLSDGEGMPREERNVGSDG
HumanPOMC           PREGKRSYSMEHFRWGKPVGKKRRPVKVYP-NGAEDESAE
MousePOMC           RSYSMEHFRWGKPVGKKRRPVKVYP-NVAENESAE-----
DogPOMC             LQEGKRSYSMEHFRWGKPVGKKRRPVKVYP-NGAEDESAE
OpossumPOMC         ELQNKRSYSMEHFRWGKPVGKKRRPVKIYP-NGVEEESAE
ChickenPOMC         REEGKRSYSMEHFRWGKPVGRKRRPIKVYP-NGVDEESAE
S.tropicalisPOMC    RQENKRAYSMEHFRWGKPVGRKRRPIKVYP-NGVEEESAE
ZebrafishPOMCa      RHDHKRSYSMEHFRWGKPVGRKRRPIKVYT-NGVEEESAE
MedakaPOMCa         SPQTKRSYSMEHFRWGKPVGRKRRPVKVYTPNGVEEESSE
SticklebackPOMCa    SPQAKRSYSMEHFRWGKPVGRKRRPVKVYAPNGVEEESAE
ZebrafishPOMCb      HGDERRSYSMEHFRWGKPMGRKRRPVKVLSNGALEEEPEE
MedakaPOMCb         SDMNRRSYAMEHFRWGKPIGRKRRPIKVFASSLEEGDSSE
SticklebackPOMCb    LSDARRSYSMEHFRWGKPTGRKRRPVKVFASSLEGGGSSE
```

```
                             210       220       230       240
                    ....|....|....|....|....|....|....|....|
HumanPENK           ER--SHHQDGSDNEEEVSKRYGGFMRGLKRSPQL------
MousePENK           AKD-SHQQESTNNDEDMSKRYGGFMRSLKRSPQL------
DogPENK             EG-G-PHQEGSD-DEDVSKRYGGFMRALKRSPQL------
OpossumPENK         EA--GRYQAGNENEEEVSKRYGGFMRSYKRSPEL------
ChickenPENK         EA--AHYRGVNENDGDVSKRYGGFMRSVKRSPEL------
S.tropicalisPENK    ESA-IYHDNNSETPGEMNKRYGGFMRGYRRSTDL------
ZebrafishPENKa      EE--AALTRSKGGEEGTAKRYGGFMR-RGGLYDL------
MedakaPENK          GE---HSRERDGQGGGAVKRYGGFMRRAEGGEGTGSLL--
SticklebackPENK     PE---QGGE--GPGGDAVKRYGGFMRRAGGGVAQGDLL--
ZebrafishPENKb      ES-------DDQPDGDMVKRYGGFMRSVQEN---------
HumanPDYN           L--------AEEDPKEQVKRYGGFLRKYPKRSSEVA----
MousePDYN           F--------NEEDLRKQAKRYGGFLRKYPKRSSEMARD--
DogPDYN             S--------NEEDPKEQVKRYGGFLRKYPKRSSEVA----
OpossumPDYN         YPGEGAAASSLETRKDEMKRYGGFLRKYPKRSFEVAG---
Zebra finchPDYN     ----------------------------------------
S.tropicalisPDYN    WFS------PTWGTKDERKRYGGFLRKYPKRILSQEGDLE
ZebrafishPDYN       YDN--------EVPLNEVKRYGGFLRKFGP--KRSYF---
MedakaPDYN          HS--------------SVKRYGGFLRKFGPKSKRSSS---
SticklebackPDYN     RG--------------YVKRYGGFLRKFGPKSKRSSS---
HumanPNOC           ----------------------------------------
MousePNOC           ----------------------------------------
DogPNOC             ----------------------------------------
OpossumPNOC         ----------------------------------------
ChickenPNOC         ------------DEDISRRLGGGFPRGTRGS----WPA--
S.tropicalisPNOC    -----EFGQLQNPANAVSKRFGGFVKGKYSYRKFMGP---
ZebrafishPNOCa      GDS-EIEGDEQESAIHLIKRFGGFLKNKYGYRKFIDP---
MedakaPNOCa         ED--ANVAGREKGDAGFSKRFGGFVKGRHGYRRLMSP---
SticklebackPNOCa    ATD-MAAGGQDGAGLSVFKRFGGFVKGRHGYRKLISP---
ZebrafishPNOCb      ------DEKSDDAAVSVSKRFGGFMKGRHGLRKLVSS---
MedakaPNOCb         QSE-MDEDNDSLQVVSLSKRFGGFQRGRHGYRKLIGSP--
SticklebackPNOCb    QPE-GLRGQEASGAVTLSKRFGGFQRGRHGYRKLIGSS--
HumanPOMC           -----------AFP-LEFKR-ELTGQRLREGDGPDGPADD
MousePOMC           ------AFP-LEFKR-ELEGER------PLG---------
DogPOMC             -----------AFP-VEFKK-ELARQRLEPALGPEGPAAG
OpossumPOMC         -----------SYP-VEIRR-DLPMKINFPEYPELAIDEE
ChickenPOMC         -----------SYP-MEFRR-EMAPDGDPFGLSEEE----
S.tropicalisPOMC    -----------NYP-MELRR-ELSLELDYPDIDLDE----
ZebrafishPOMCa      -----------TLP-AEMRR-ELANNEVDYPQEE------
MedakaPOMCa         -----------VFP-GEMRRRELANELLAAAAEEEERAME
SticklebackPOMCa    -----------LYPPGEMRRRELMEDEKKAQQHQREEGEE
ZebrafishPOMCb      -----------SEESVRVERGQSGTLEVQHRNN-------
MedakaPOMCb         R----------SFPFRVRRHLSSDKNEAKGMHHEASHRNL
SticklebackPOMCb    R----------GFPALARRQLGLNKYKARDLK-EGSRGQA
```

Sequence	250 · 260 · 270 · 280
HumanPENK	--------------------------------EDEAKELQ
MousePENK	--------------------------------EDEAKELQ
DogPENK	--------------------------------EEEAKELQ
OpossumPENK	--------------------------------EDEAKELQ
ChickenPENK	--------------------------------EDEAKELQ
S.tropicalisPENK	--------------------------------EDETRGIQ
ZebrafishPENKa	--------------------------------ESGVRELQ
MedakaPENK	-------------------------------EAVLDRGLK
SticklebackPENK	-------------------------------EAVLGRGLK
ZebrafishPENKb	----------------------------------TGRDLH
HumanPDYN	------------------------GEG--DGDSMGHEDLY
MousePDYN	------------------------EDGGQDGDQVGHEDLY
DogPDYN	------------------------GEGNGDGDAVGHEDLY
OpossumPDYN	-----------------------------AGDVQEQEDLH
Zebra finchPDYN	--------------------------------------LH
S.tropicalisPDYN	RRRRLQEGLEAGQAIVTGQEI---ETGQELDLERGTAELE
ZebrafishPDYN	------------------------------VDDTNPQVLQ
MedakaPDYN	----------------------------AEQESQEPEELQ
SticklebackPDYN	----------------------------AETDSQEPEELQ
HumanPNOC	--
MousePNOC	--
DogPNOC	--
OpossumPNOC	--
ChickenPNOC	-----------------------------------ARGVQ
S.tropicalisPNOC	-----------------------------------SKDLQ
ZebrafishPNOCa	-----------------------------------GRSLQ
MedakaPNOCa	-----------------------------------GRSYQ
SticklebackPNOCa	-----------------------------------GRLYQ
ZebrafishPNOCb	-----------------------------------GRPLQ
MedakaPNOCb	-----------------------------------MRPLQ
SticklebackPNOCb	-----------------------------------MRPLQ
HumanPOMC	GAGAQADLEHSLLVAA---EKKDEGPYRMEHFRWGSPPKD
MousePOMC	--LEQVLESDA----EKDDGPYRVEHFRWSNPPKDKRYGG
DogPOMC	-VAALADLEYGLVAEAGAAEKKDDGPYKMEHFRWGSPPKD
OpossumPOMC	------EAAKEVYEEK---VKKDGGGYKMEHFRWGTPPKD
ChickenPOMC	------EEEEEEEGEE---EKKDGGSYRMRHFRWHAPLKD
S.tropicalisPOMC	------DIEDNEVESA---LTKKNGNYRMHHFRWGSPPKD
ZebrafishPOMCa	----------MPLNPL----GKKDPPYKMTHFRWSVPPAS
MedakaPOMCa	EVEEEEERQHLLAGLQ----EKKDGSYKMKHFRWSGPPAS
SticklebackPOMCa	EGEEE-----LPGDHH----EKKDGAYKMKHFRWSGPPAS
ZebrafishPOMCb	--------------------VKNNGKYRMTHFRWNAPP-D
MedakaPOMCb	GLLVSKFPPRKDVPLQ----DRKEETYRMNHFRWGSPRTS
SticklebackPOMCb	PQRVG-HKAQAPVSLP----ARKDGAYRMSHFRWGSPPPS

	290 300 310 320
HumanPENK	KRYGGFMRRVGRP--EWWMDYQKRYGGFLKRFAEA-LPSD
MousePENK	KRYGGFMRRVGRP--EWWMDYQKRYGGFLKRFAES-LPSD
DogPENK	KRYGGFMRRVGRP--EWWMDYQKRYGGFLKRFADS-LPSD
OpossumPENK	KRYGGFMRRVGRP--EWWLDYQKRYGGFLKRFADS-LPSD
ChickenPENK	KRYGGFMRRVGRP--EWWLDYQKRYGGFLKRFADSILPSE
S.tropicalisPENK	KRYGGFMRRVGRP--EWWQDYQKRYGGFMRRFTDSFLPSE
ZebrafishPENKa	KRYGGFMRRVGRP--DWWQE-SKRYGGFLKRSQEQ-----
MedakaPENK	KRYGGFMRRVGRP--EWLVD-GSKNGGVFKRAWEN-----
SticklebackPENK	KRYGGFMRRVGRP--EWLVD-SSKSGGMLKRAWES-----
ZebrafishPENKb	KRYGGFMRRVGRP--DWLDN--QKSGGLLKRTWEE-----
HumanPDYN	KRYGGFLRRIRPK---LKWDNQKRYGGFLRRQFKVVTRSQ
MousePDYN	KRYGGFLRRIRPK---LKWDNQKRYGGFLRRQFKVVTRSQ
DogPDYN	KRYGGFLRRIRPK---LKWDNQKRYGGFLRRQFKVVTRSQ
OpossumPDYN	KRYGGFMRRIRPK---LKWDNQKRYGGFLRRQFKVVTRSE
Zebra finchPDYN	KRYGGFLRRIRPK---LKWDNQKRYGGFLRRQFKVTTRSD
S.tropicalisPDYN	KRYGGFLRRIRPK---LRWDNQKRYGGFLRRQFKVNARSE
ZebrafishPDYN	KRYGGFMRRIRPK---LRWDNQKRYGGFLRRHFKISVRSD
MedakaPDYN	KRYGGFMRRVRPKLNNLKWD--KRYGGFLRRHFKISVRSL
SticklebackPDYN	KRYGGFMRRIRPKSNNLKWD--KRYGGFLRRHFKISVRSA
HumanPNOC	KRFGGFTGARKSA---RKLANQKRFSEFMRQYLVLSMQSS
MousePNOC	KRFGGFTGARKSA---RKLANQKRFSEFMRQYLVLSMQSS
DogPNOC	KRFGGFTGARKSA---RKLANQKRFSEFMRQYLVLSMQSS
OpossumPNOC	KRFGGFTGARKSA---RKLANQKRFSEFMRQYLVMSMHAS
ChickenPNOC	KRYGGFIGVRKSA---RKWNNQKRFSEFLKQYLGM---SP
S.tropicalisPNOC	KRYGGFIGVRKSA---RKWNNQKRFSEFLRQYLCMSTRSA
ZebrafishPNOCa	KRYGGFIGVRKSA---RKWNNQKRFSEFLKQYLGM---SS
MedakaPNOCa	KRYGGFIGIRKSA---RKWNNQKRFSEFLKQYLGM---ST
SticklebackPNOCa	KRYGGFVGIRKSA---RKWNNQKRFSDLLKQYLGR---SA
ZebrafishPNOCb	KRYGGFIGIRKSA---RKWNNQKRVSQLLRQYLSLTGRSG
MedakaPNOCb	KRYGGFIGVRKSA---RKWNSQKRVNQLLRQYLGM--RSS
SticklebackPNOCb	KRYGGFIGVRKSA---RKWNSQKRVNQLLRQYLGL--RSS
HumanPOMC	KRYGGFMTS--EKSQTPLVTLFKNAIIKNAYKKGE-----
MousePOMC	FMTS--EKSQTPLVTLFKNAIIKNAHKKGQ--------
DogPOMC	KRYGGFMSS--ERSQTPLVTLFKNAIIKNAHKKGQ-----
OpossumPOMC	KRYGGFMIS--EKSHTPLMTLFKNAIIKNGHKKGQ-----
ChickenPOMC	KRYGGFMSL--EHSQTPLMTLFKNAIVKSAYKKGQ-----
S.tropicalisPOMC	KRYGGFMTP--ERSQTPLMTLFKNAIIKNTHKKGQ-----
ZebrafishPOMCa	KRYGGFMKSWDERAQKPLLTLFKNVMHKDQPRKDE-----
MedakaPOMCa	KRYGGFMKSWEEDRQKPLVTLFKNIINKDEQQ--------
SticklebackPOMCa	KRYGGFMRSWDERDPRPLLTLLKNVLNTEGRQ--------
ZebrafishPOMCb	KRYGGLMRPYLDESHKPLITLIRNAIGNGQQFTD------
MedakaPOMCb	KRNGSFRKLWEESSEENPATLLKNIIFMKDVQR-KID---
SticklebackPOMCb	KRNGSTMRQPRKKPKGQLDKLLRN-IFLKDVQRSRMG---

```
                                        330                 340
                           . . . . | . . . . | . . . . | . . . . | . . . . | . . .
HumanPENK                  E E G E S Y S K E V P E M E K R Y G G F M R F - -
MousePENK                  E E G E N Y S K E V P E I E K R Y G G F M R F - -
DogPENK                    E E G E S Y S K E V P E M E K R Y G G F M R F A - - A -
OpossumPENK                E E G E S Y S K E V P E M E K R Y G G F M R F - -
ChickenPENK                E D G E T Y S K E V P E M E K R Y G G F M R F - -
S.tropicalisPENK           E D G E S Y S K E N P D M E K R Y G G F M R F - -
ZebrafishPENKa             D E D E N S S - - - - E V E K R Y G G F M G Y - -
MedakaPENK                 - - - - - - - - - G S G L Q K R Y G G F M D - - -
SticklebackPENK            - - - - - - - - - G S E L Q K R Y G G F M D - - -
ZebrafishPENKb             - G G E T A L - - - P D M Q K R Y G G F M D - - -
HumanPDYN                  E D P N A Y S G E L F D A - - A - - - - - - - - - -
MousePDYN                  E N P N T Y S - E D L D V - - A - - - - - - - - - -
DogPDYN                    E D P N A Y S G E L L D G - - A - - - - - - - - - -
OpossumPDYN                E D P N A Y S G E V A G L - - A - - - - - - - - - -
Zebra finchPDYN            E D P S A Y S G E V S D L - - A - - - - - - - - - -
S.tropicalisPDYN           E D P T M F S G E L S Y L - - A - - - - - - - - - -
ZebrafishPDYN              E E P S - - S Y E D Y A L - - A - - - - - - - - - -
MedakaPDYN                 E E P V Y S Y D E P S L Q T N A - - - - - - - - - -
SticklebackPDYN            E E P Y Y S Y D D L S L - - - A - - - - - - - - - -
HumanPNOC                  Q R R - - - - - - - R T L H Q N G N V - - - - - -
MousePNOC                  Q R R - - - - - - - R T L H Q N G N V - - - - - -
DogPNOC                    Q R R - - - - - - - R T L H Q N G N V - - - - - -
OpossumPNOC                E H Q R H G L A P G R N L R Q N S N V - - - - - -
ChickenPNOC                R S S E Y D - - M A G D L S E H N E I - - - - - -
S.tropicalisPNOC           E Y D S F T N - D V S D I N G Q N E I - - - - - -
ZebrafishPNOCa             R A S E Y N - S M S A D L T Q Q N E - - - - - - -
MedakaPNOCa                R A T E F N - S L S E D L T E Q N E V - - - - - -
SticklebackPNOCa           K A T E L N - S V S K D L D Q Q N E V - - - - - -
ZebrafishPNOCb             R S G H I N - S L S T R I R R Q S E V - - - - - -
MedakaPNOCb                R S G R F N - A S M N R A W R Q N K L - - - - - -
SticklebackPNOCb           R S G R V G Y A P V T R V W R Q N Q L - - - - - -
HumanPOMC                  - - -
MousePOMC
DogPOMC                    - - -
OpossumPOMC                - - -
ChickenPOMC                - - -
S.tropicalisPOMC           - - -
ZebrafishPOMCa             - - -
MedakaPOMCa                - - -
SticklebackPOMCa           - - -
ZebrafishPOMCb             - - -
MedakaPOMCb                - - -
SticklebackPOMCb           - - -
```
