# Supplementary material for: Concomitant Duplications of Opioid Peptide and Receptor Genes before the Origin of Jawed Vertebrates
Source: PLoS One. 2010 May 6;5(5):e10512. doi: 10.1371/journal.pone.0010512 (PMC2865548; doi:10.1371/journal.pone.0010512)
Supplement: Figure S2 — Neighbor-joining and quartet-puzzling maximum likelihood trees for all families included in the study. (0.96 MB PDF) [file pone.0010512.s004.pdf]

BMP, NJ

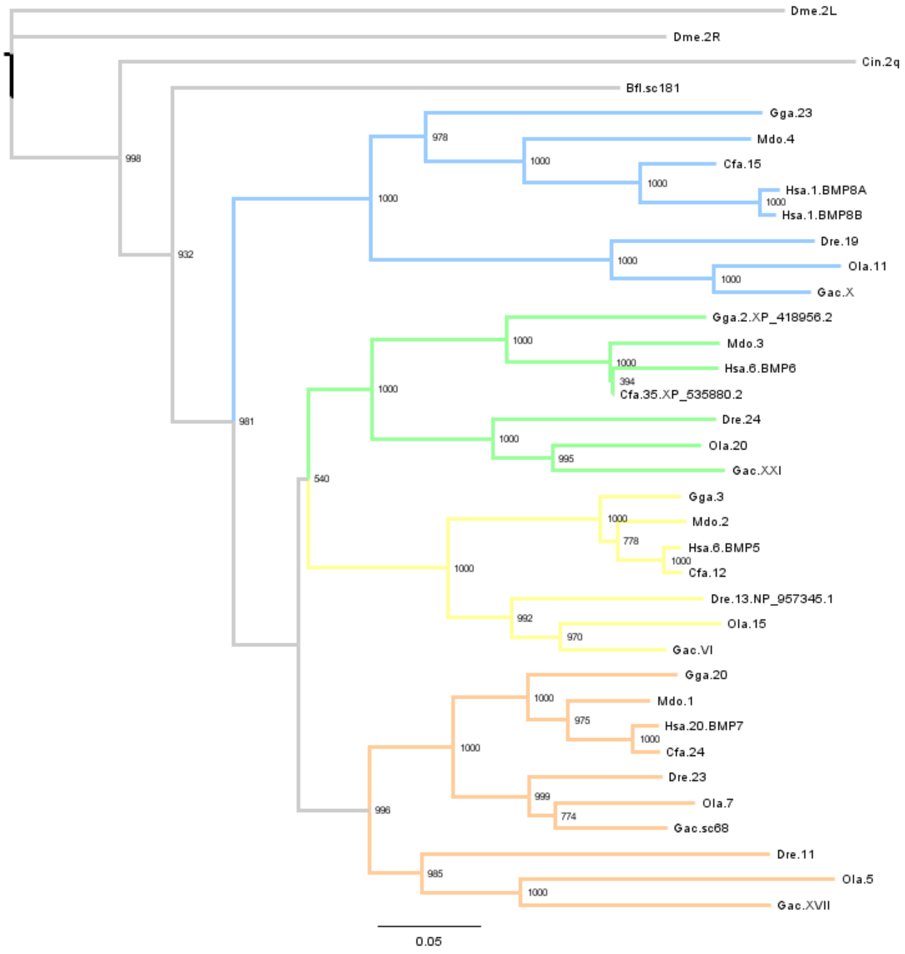

Dme.2L
Dme.2R
Cin.2q
Bfl.sc181
Gga.23
Mdo.4
Cfa.15
Hsa.1.BMP8A
Hsa.1.BMP8B
Dre.19
Ola.11
Gac.X
Gga.2.XP_418956.2
Mdo.3
Hsa.6.BMP6
Cfa.35.XP_535880.2
Dre.24
Ola.20
Gac.XXI
Gga.3
Mdo.2
Hsa.6.BMP5
Cfa.12
Dre.13.NP_957345.1
Ola.15
Gac.VI
Gga.20
Mdo.1
Hsa.20.BMP7
Cfa.24
Dre.23
Ola.7
Gac.sc68
Dre.11
Ola.5
Gac.XVII
0.05

BMP, QP

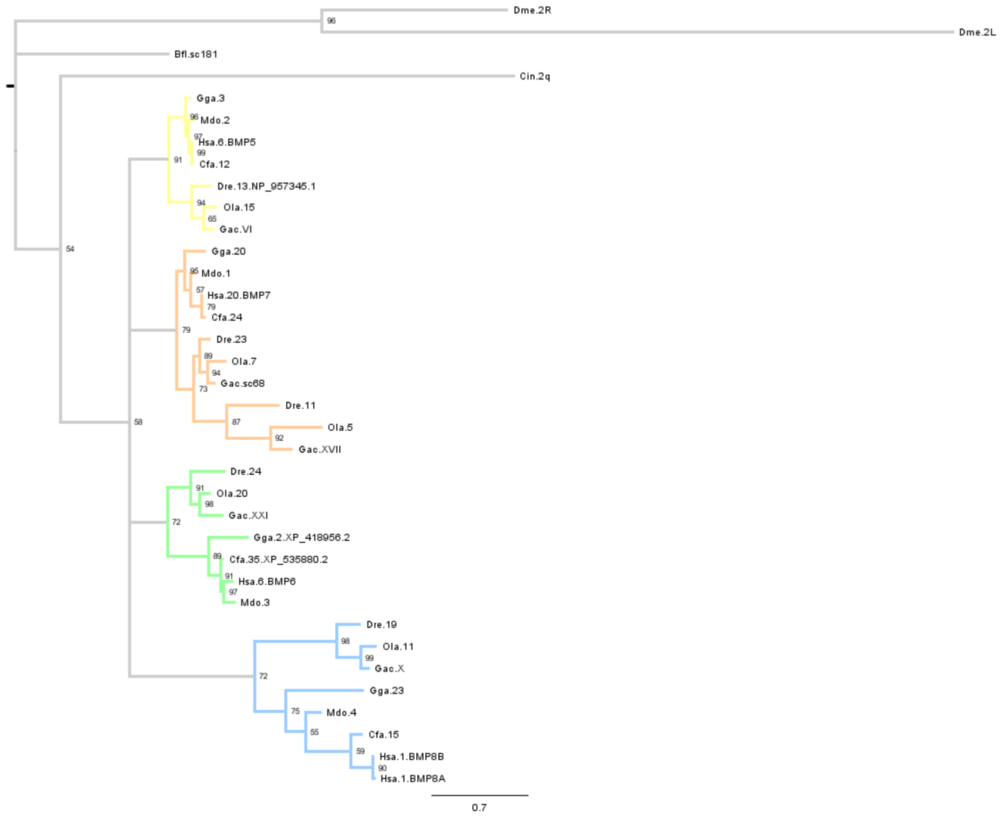

Dme.2R
Dme.2L
Bfl.sc181
Cin.2q
Gga.3
Mdo.2
Hsa.6.BMP5
Cfa.12
Dre.13.NP_957345.1
Ola.15
Gac.VI
Gga.20
Mdo.1
Hsa.20.BMP7
Cfa.24
Dre.23
Ola.7
Gac.sc68
Dre.11
Ola.5
Gac.XVII
Dre.24
Ola.20
Gac.XXI
Gga.2.XP_418956.2
Cfa.35.XP_535880.2
Hsa.6.BMP6
Mdo.3
Dre.19
Ola.11
Gac.X
Gga.23
Mdo.4
Cfa.15
Hsa.1.BMP8B
Hsa.1.BMP8A
0.7

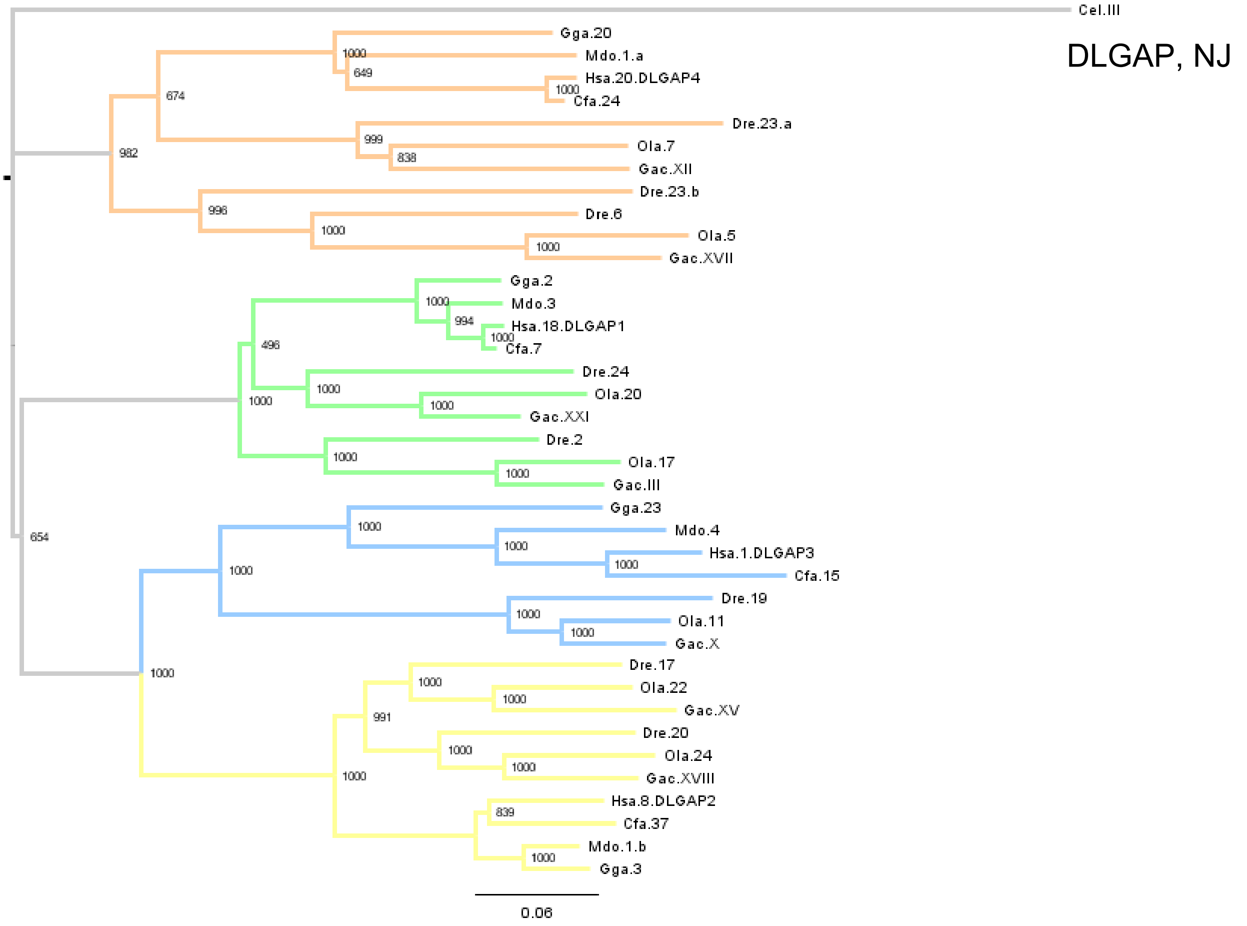
DLGAP, NJ
Cel.III
Gga.20
Mdo.1.a
Hsa.20.DLGAP4
Cfa.24
Dre.23.a
Ola.7
Gac.XII
Dre.23.b
Dre.6
Ola.5
Gac.XVII
Gga.2
Mdo.3
Hsa.18.DLGAP1
Cfa.7
Dre.24
Ola.20
Gac.XXI
Dre.2
Ola.17
Gac.III
Gga.23
Mdo.4
Hsa.1.DLGAP3
Cfa.15
Dre.19
Ola.11
Gac.X
Dre.17
Ola.22
Gac.XV
Dre.20
Ola.24
Gac.XVIII
Hsa.8.DLGAP2
Cfa.37
Mdo.1.b
Gga.3
0.06

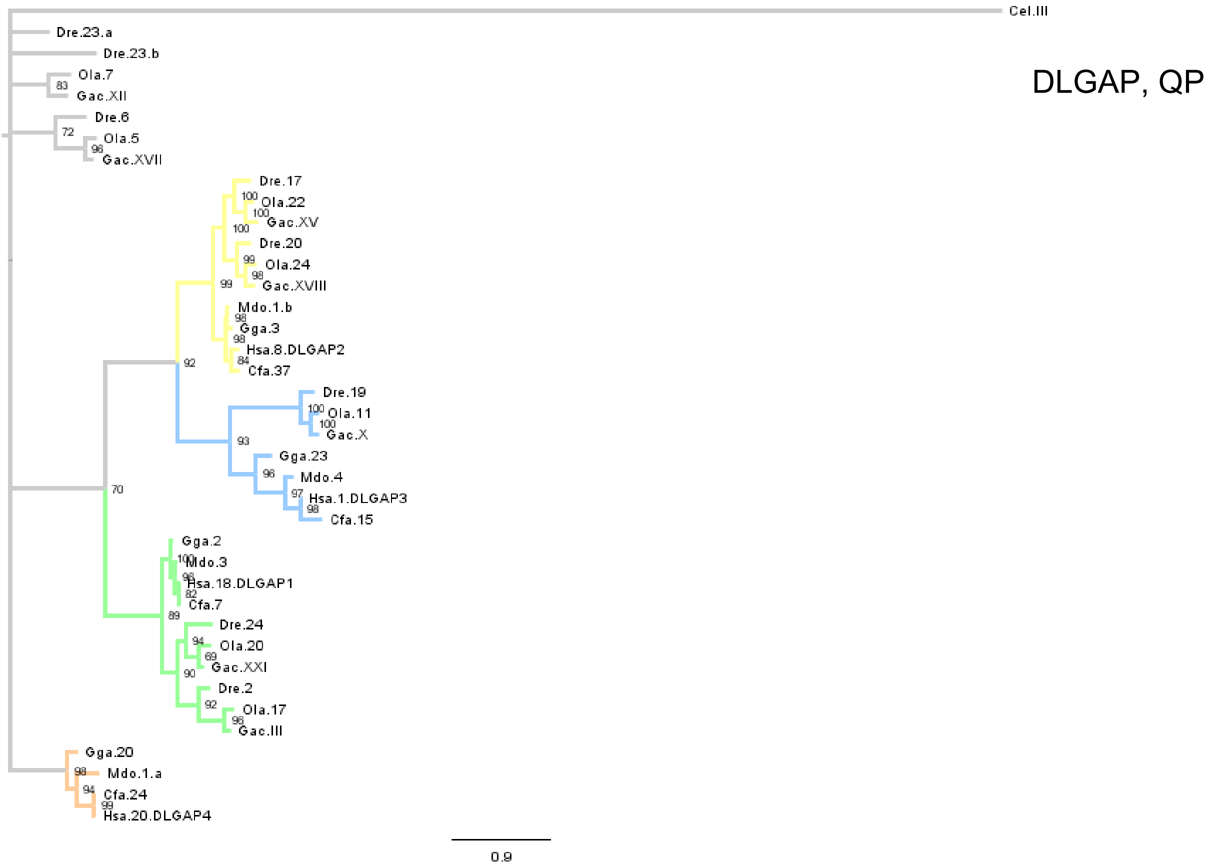
DLGAP, QP
Cel.III
Dre.23.a
Dre.23.b
Ola.7
Gac.XII
Dre.6
Ola.5
Gac.XVII
Dre.17
Ola.22
Gac.XV
Dre.20
Ola.24
Gac.XVIII
Mdo.1.b
Gga.3
Hsa.8.DLGAP2
Cfa.37
Dre.19
Ola.11
Gac.X
Gga.23
Mdo.4
Hsa.1.DLGAP3
Cfa.15
Gga.2
Mdo.3
Hsa.18.DLGAP1
Cfa.7
Dre.24
Ola.20
Gac.XXI
Dre.2
Ola.17
Gac.III
Gga.20
Mdo.1.a
Cfa.24
Hsa.20.DLGAP4
0.9

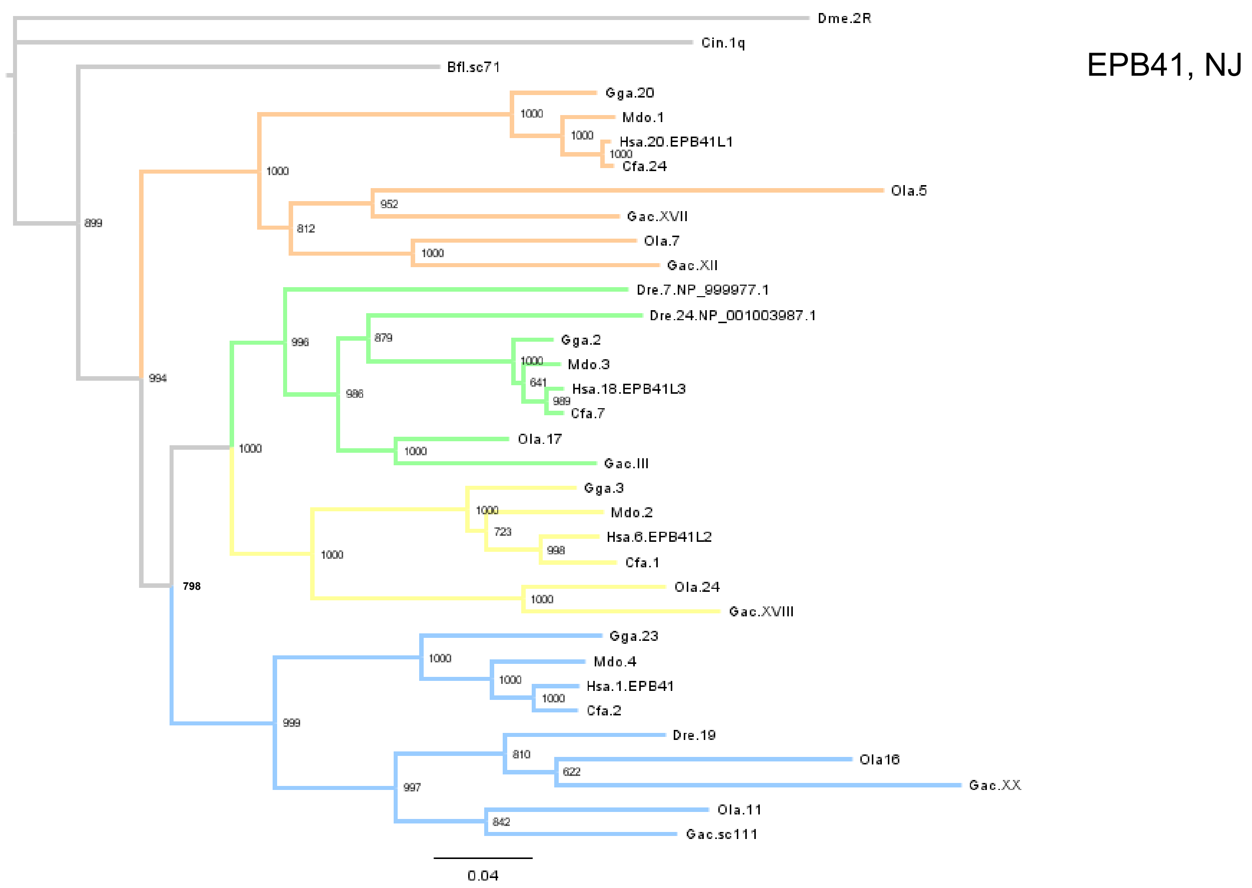
EPB41, NJ
Dme.2R
Cin.1q
Bfl.sc71
Gga.20
Mdo.1
Hsa.20.EPB41L1
Cfa.24
Ola.5
Gac.XVII
Ola.7
Gac.XII
Dre.7.NP_999977.1
Dre.24.NP_001003987.1
Gga.2
Mdo.3
Hsa.18.EPB41L3
Cfa.7
Ola.17
Gac.III
Gga.3
Mdo.2
Hsa.6.EPB41L2
Cfa.1
Ola.24
Gac.XVIII
Gga.23
Mdo.4
Hsa.1.EPB41
Cfa.2
Dre.19
Ola16
Gac.XX
Ola.11
Gac.sc111
0.04

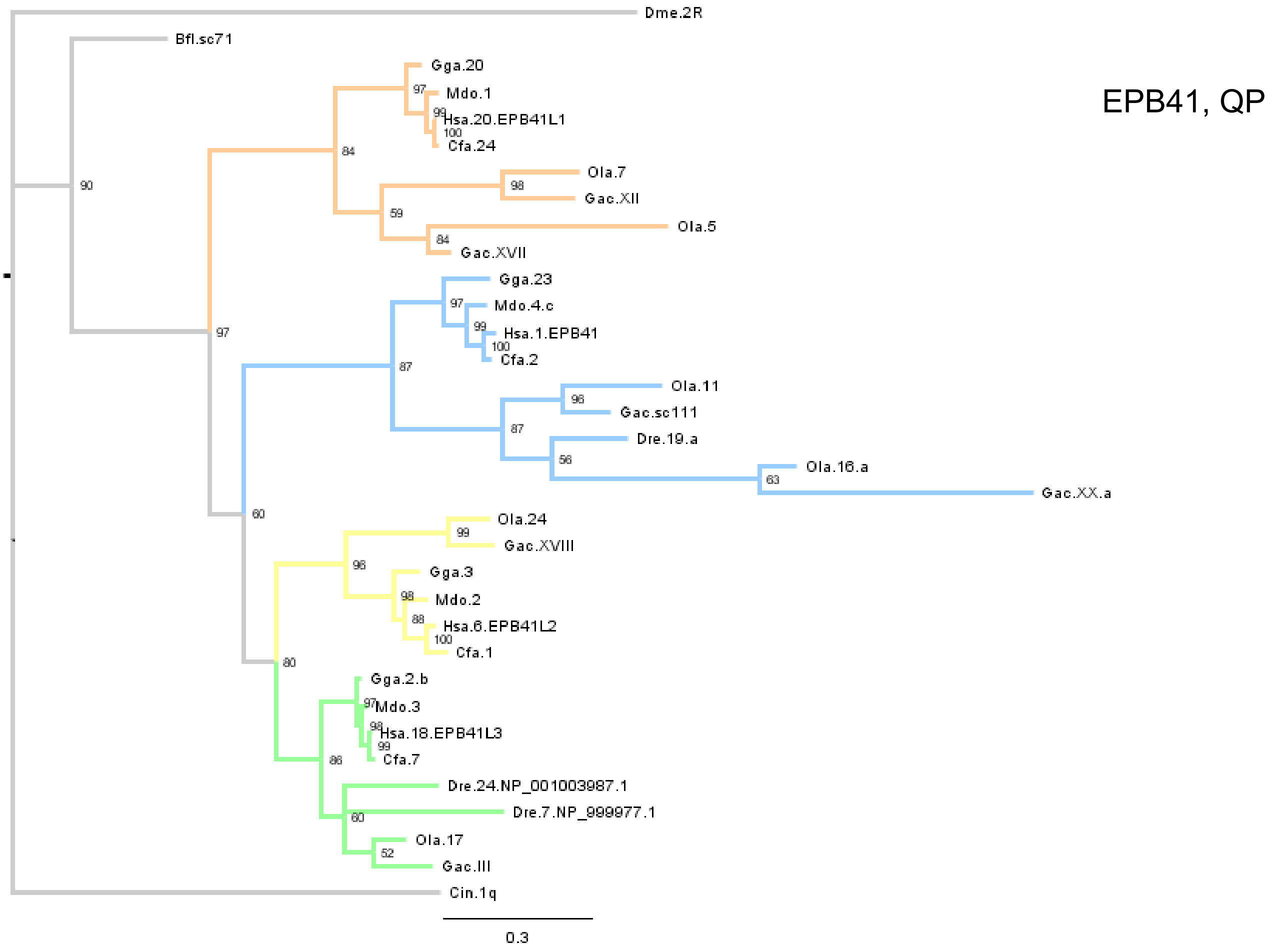
EPB41, QP
Dme.2R
Bfl.sc71
Gga.20
Mdo.1
Hsa.20.EPB41L1
Cfa.24
Ola.7
Gac.XII
Ola.5
Gac.XVII
Gga.23
Mdo.4.c
Hsa.1.EPB41
Cfa.2
Ola.11
Gac.sc111
Dre.19.a
Ola.16.a
Gac.XX.a
Ola.24
Gac.XVIII
Gga.3
Mdo.2
Hsa.6.EPB41L2
Cfa.1
Gga.2.b
Mdo.3
Hsa.18.EPB41L3
Cfa.7
Dre.24.NP_001003987.1
Dre.7.NP_999977.1
Ola.17
Gac.III
Cin.1q
0.3

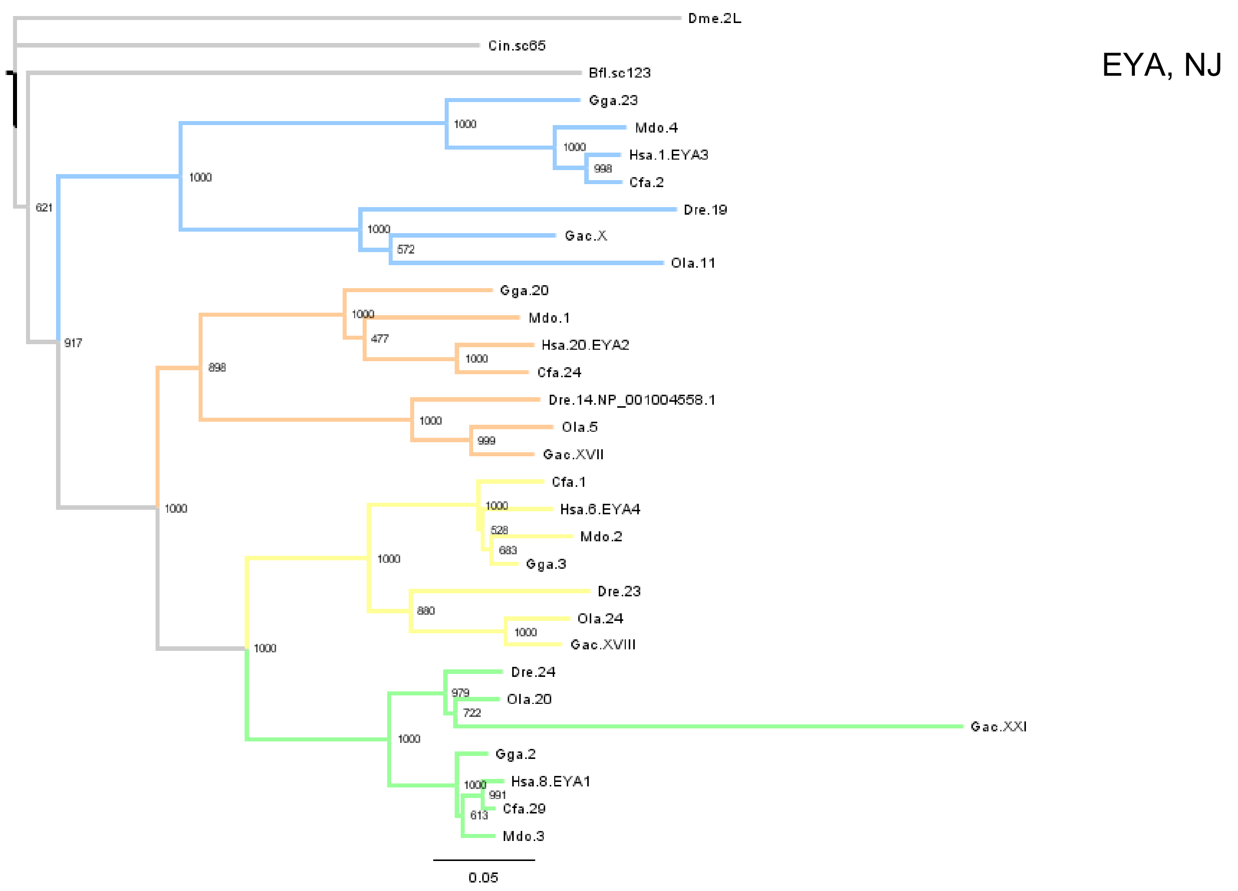
EYA, NJ
Dme.2L
Cin.sc65
Bfl.sc123
Gga.23
Mdo.4
Hsa.1.EYA3
Cfa.2
Dre.19
Gac.X
Ola.11
Gga.20
Mdo.1
Hsa.20.EYA2
Cfa.24
Dre.14.NP_001004558.1
Ola.5
Gac.XVII
Cfa.1
Hsa.6.EYA4
Mdo.2
Gga.3
Dre.23
Ola.24
Gac.XVIII
Dre.24
Ola.20
Gac.XXI
Gga.2
Hsa.8.EYA1
Cfa.29
Mdo.3
0.05

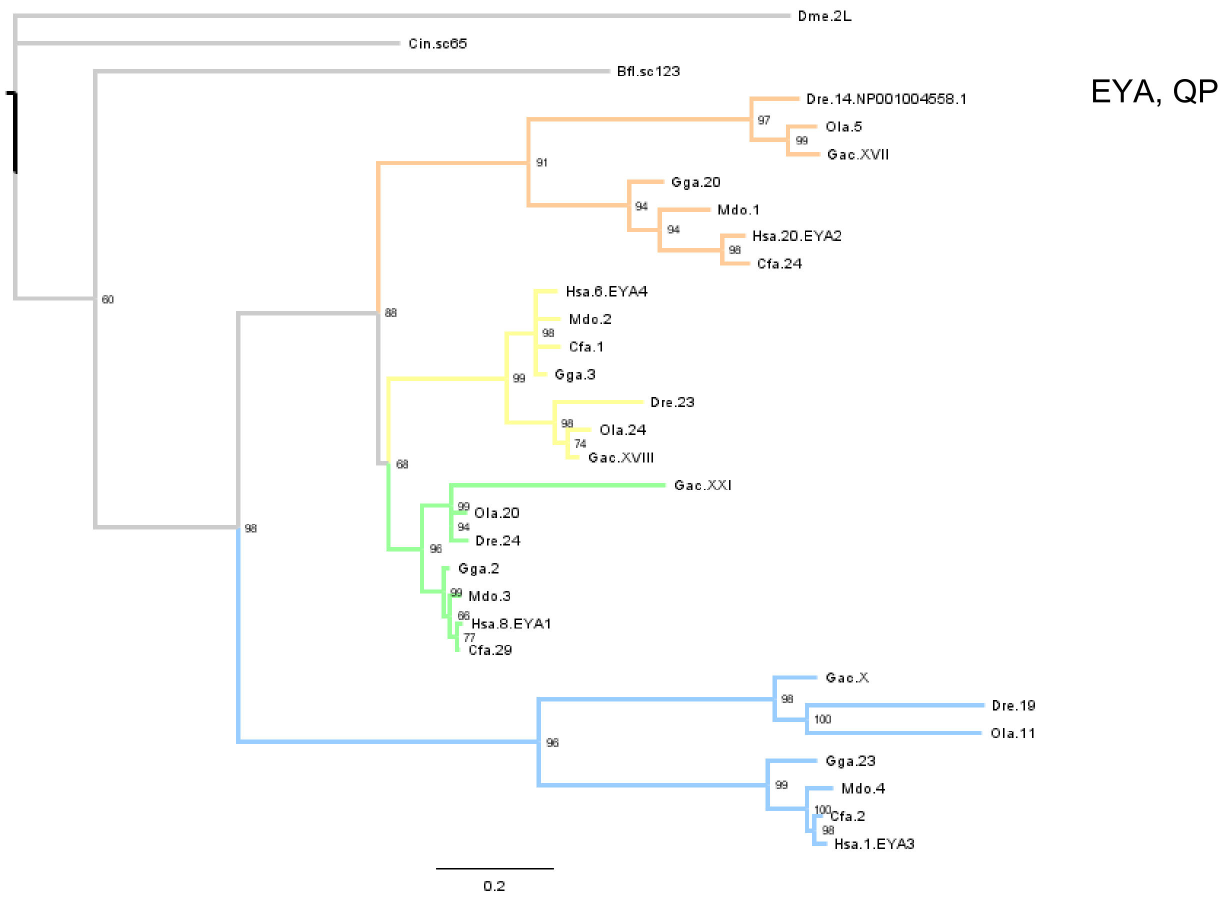
EYA, QP
Dme.2L
Cin.sc65
Bfl.sc123
Dre.14.NP001004558.1
Ola.5
Gac.XVII
Gga.20
Mdo.1
Hsa.20.EYA2
Cfa.24
Hsa.6.EYA4
Mdo.2
Cfa.1
Gga.3
Dre.23
Ola.24
Gac.XVIII
Gac.XXI
Ola.20
Dre.24
Gga.2
Mdo.3
Hsa.8.EYA1
Cfa.29
Gac.X
Dre.19
Ola.11
Gga.23
Mdo.4
Cfa.2
Hsa.1.EYA3
0.2

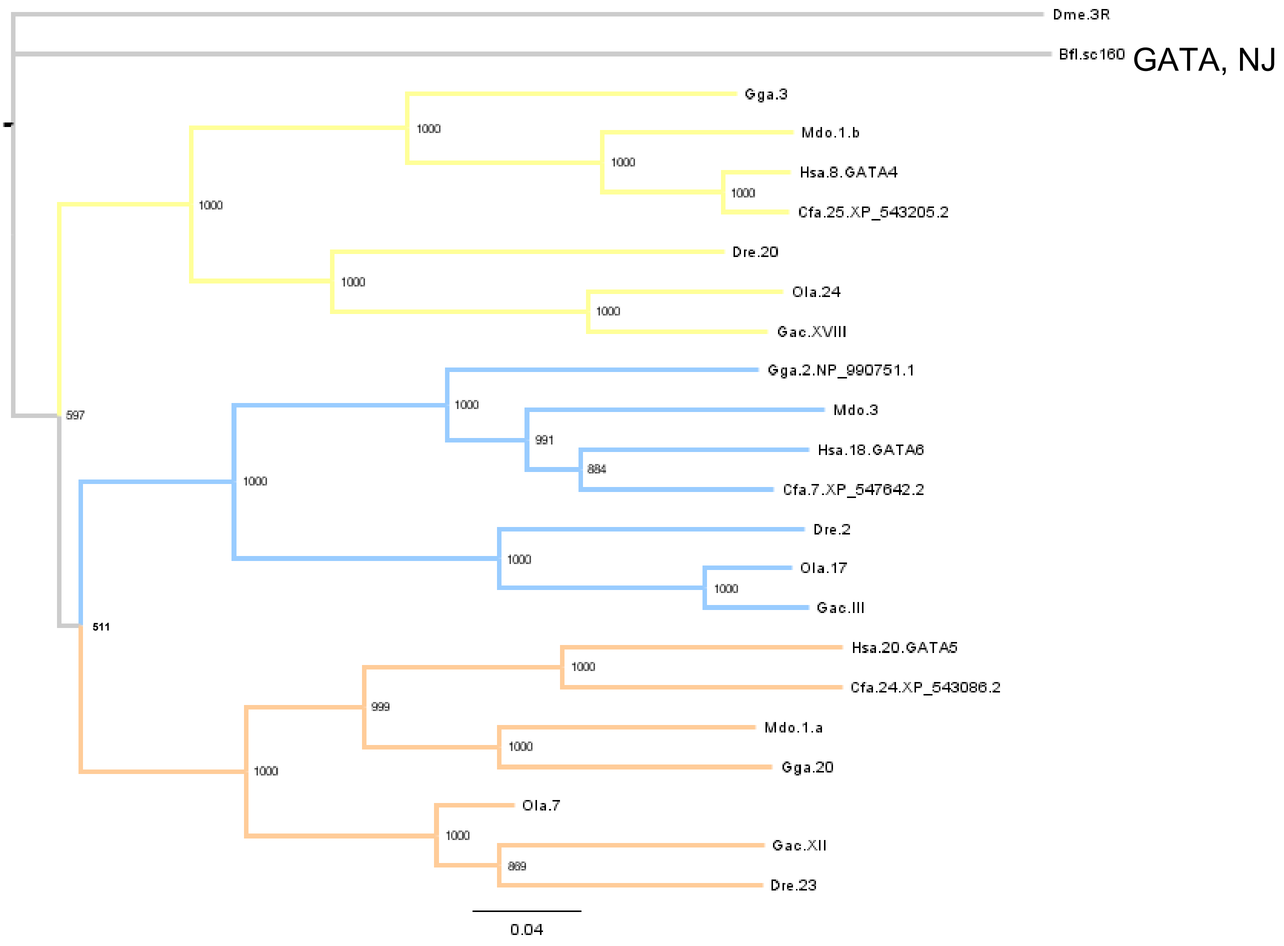
GATA, NJ
Dme.3R
Bfl.sc160
Gga.3
Mdo.1.b
Hsa.8.GATA4
Cfa.25.XP_543205.2
Dre.20
Ola.24
Gac.XVIII
Gga.2.NP_990751.1
Mdo.3
Hsa.18.GATA6
Cfa.7.XP_547642.2
Dre.2
Ola.17
Gac.III
Hsa.20.GATA5
Cfa.24.XP_543086.2
Mdo.1.a
Gga.20
Ola.7
Gac.XII
Dre.23
1000
597
511
991
884
999
869
0.04

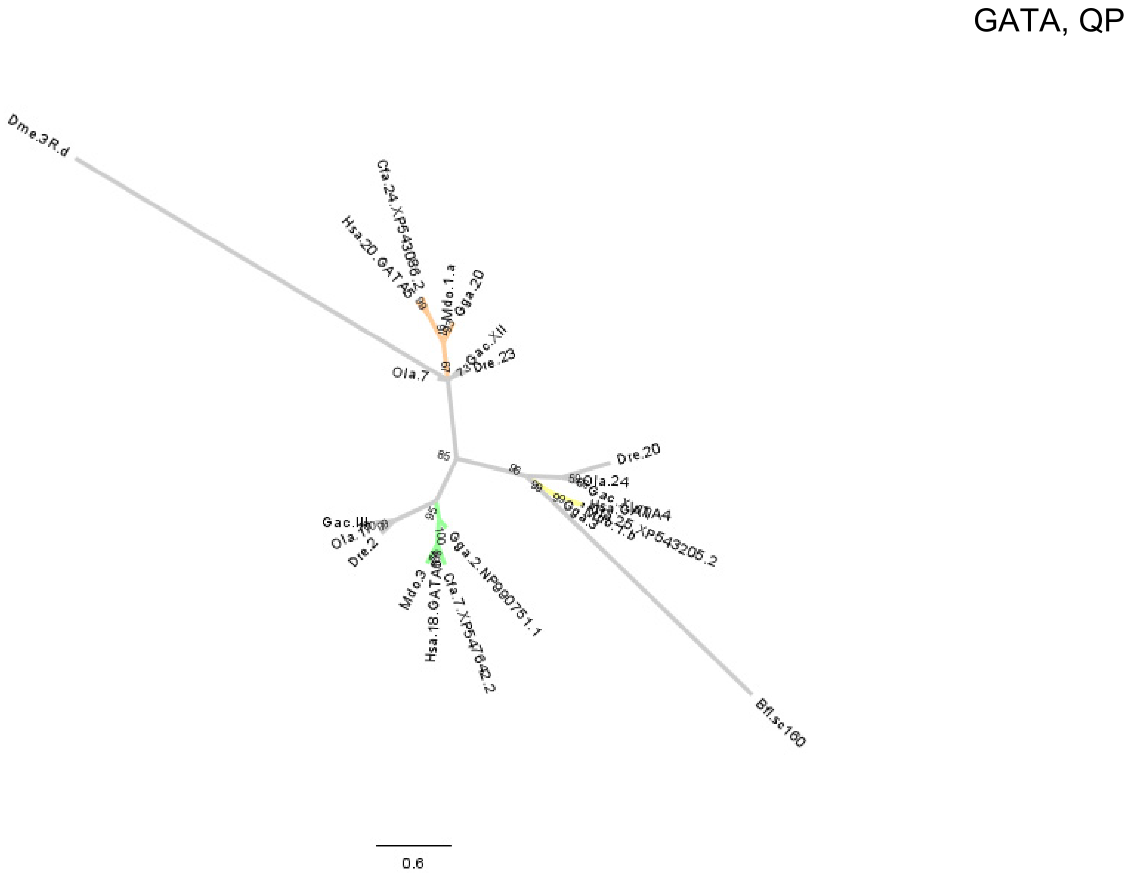
GATA, QP
Dme.3R.d
Cfa.24.XP543086.2
Hsa.20.GATA5
Mdo.1.a
Gga.20
Gac.XII
Dre.23
Ola.7
Dre.20
Ola.24
Gac.III
Ola.17
Dre.2
Mdo.3
Hsa.18.GATA6
Cfa.7.XP547642.2
Gga.2.NP990751.1
Bfl.sc160
0.6

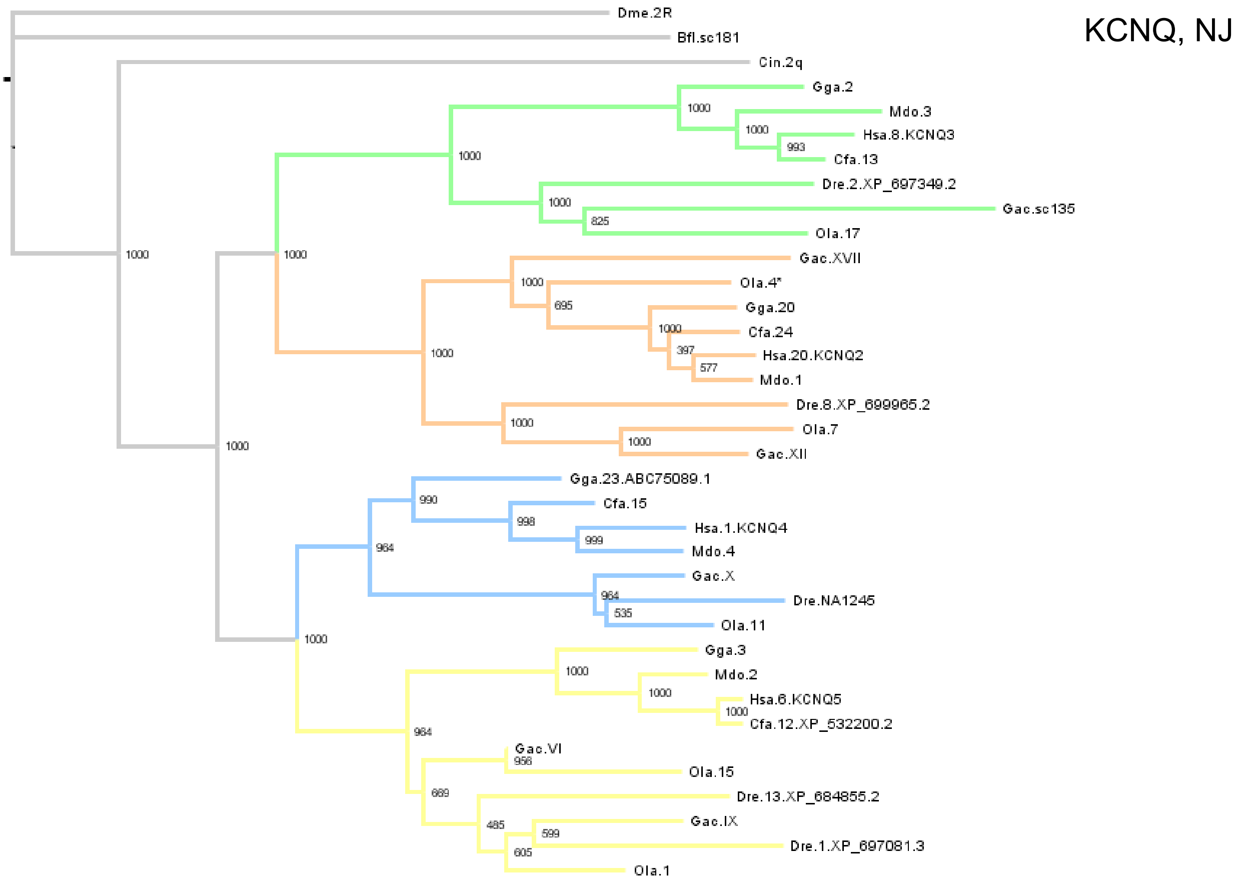
KCNQ, NJ
Dme.2R
Bfl.sc181
Cin.2q
Gga.2
Mdo.3
Hsa.8.KCNQ3
Cfa.13
Dre.2.XP_697349.2
Gac.sc135
Ola.17
Gac.XVII
Ola.4*
Gga.20
Cfa.24
Hsa.20.KCNQ2
Mdo.1
Dre.8.XP_699965.2
Ola.7
Gac.XII
Gga.23.ABC75089.1
Cfa.15
Hsa.1.KCNQ4
Mdo.4
Gac.X
Dre.NA1245
Ola.11
Gga.3
Mdo.2
Hsa.6.KCNQ5
Cfa.12.XP_532200.2
Gac.VI
Ola.15
Dre.13.XP_684855.2
Gac.IX
Dre.1.XP_697081.3
Ola.1

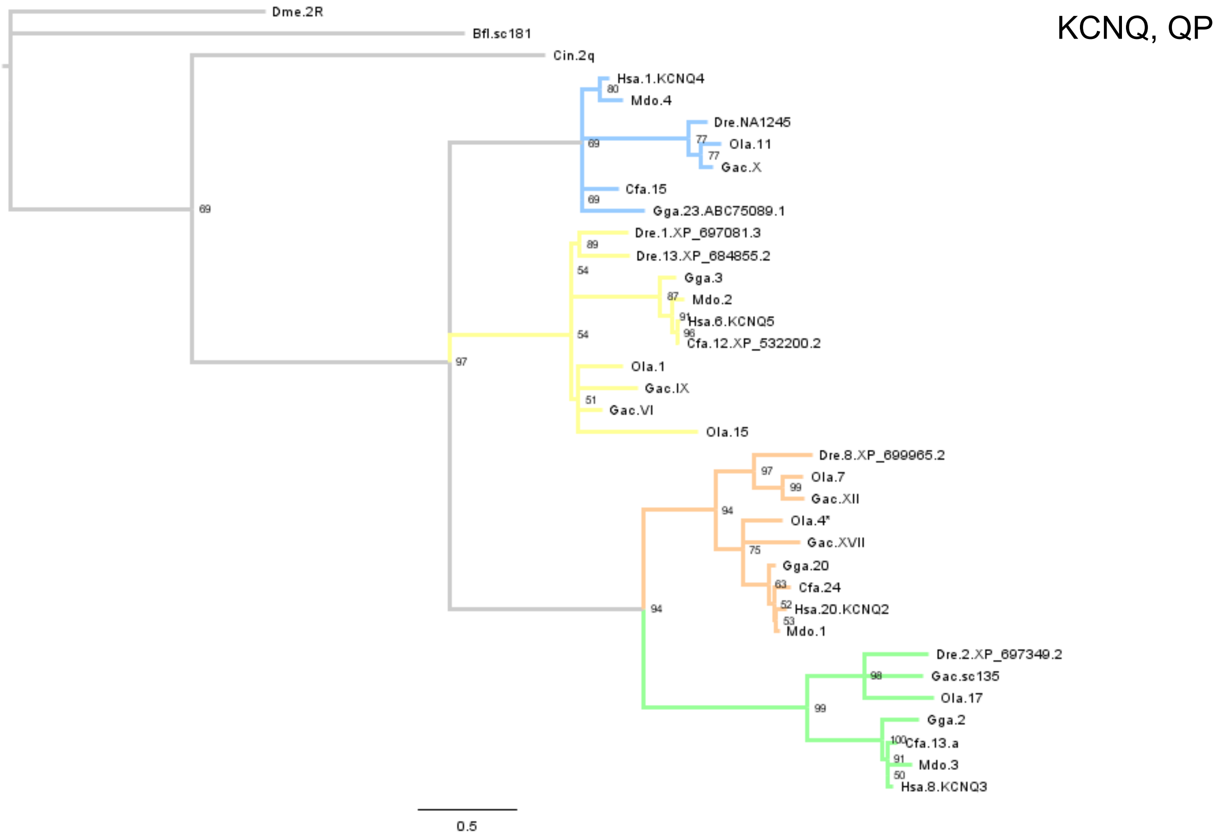
KCNQ, QP
Dme.2R
Bfl.sc181
Cin.2q
Hsa.1.KCNQ4
Mdo.4
Dre.NA1245
Ola.11
Gac.X
Cfa.15
Gga.23.ABC75089.1
Dre.1.XP_697081.3
Dre.13.XP_684855.2
Gga.3
Mdo.2
Hsa.6.KCNQ5
Cfa.12.XP_532200.2
Ola.1
Gac.IX
Gac.VI
Ola.15
Dre.8.XP_699965.2
Ola.7
Gac.XII
Ola.4*
Gac.XVII
Gga.20
Cfa.24
Hsa.20.KCNQ2
Mdo.1
Dre.2.XP_697349.2
Gac.sc135
Ola.17
Gga.2
Cfa.13.a
Mdo.3
Hsa.8.KCNQ3
0.5

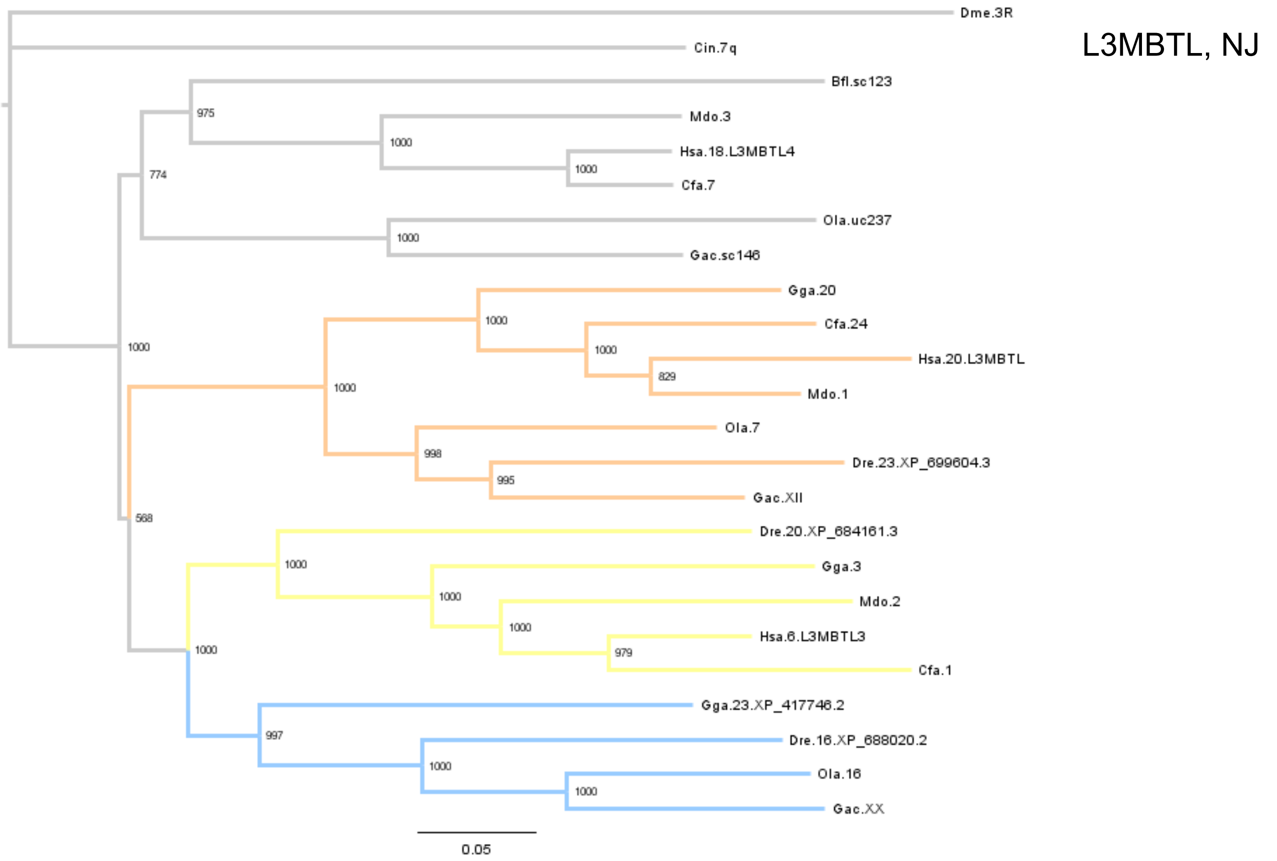
L3MBTL, NJ
Dme.3R
Cin.7q
Bfl.sc123
Mdo.3
Hsa.18.L3MBTL4
Cfa.7
Ola.uc237
Gac.sc146
Gga.20
Cfa.24
Hsa.20.L3MBTL
Mdo.1
Ola.7
Dre.23.XP_699604.3
Gac.XII
Dre.20.XP_684161.3
Gga.3
Mdo.2
Hsa.6.L3MBTL3
Cfa.1
Gga.23.XP_417746.2
Dre.16.XP_688020.2
Ola.16
Gac.XX
0.05

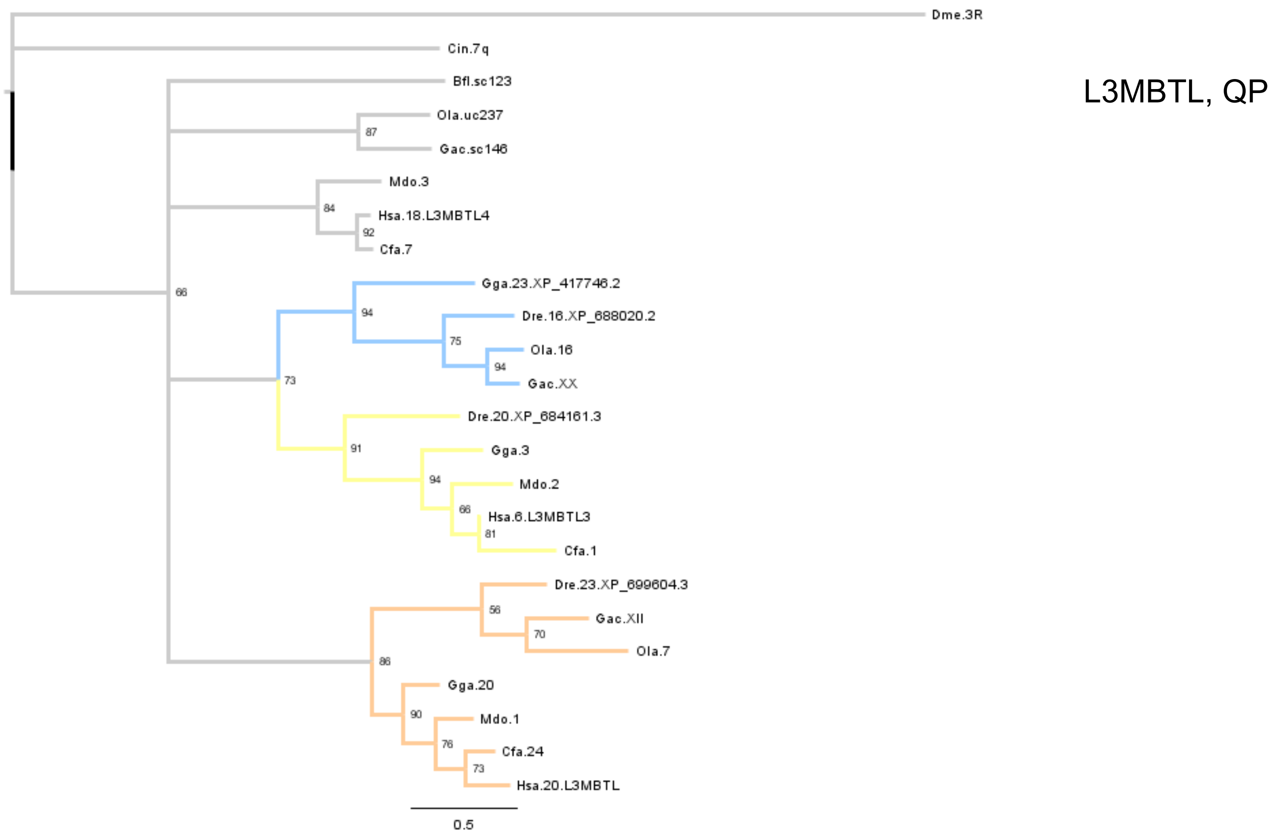
L3MBTL, QP
Dme.3R
Cin.7q
Bfl.sc123
Ola.uc237
Gac.sc146
Mdo.3
Hsa.18.L3MBTL4
Cfa.7
Gga.23.XP_417746.2
Dre.16.XP_688020.2
Ola.16
Gac.XX
Dre.20.XP_684161.3
Gga.3
Mdo.2
Hsa.6.L3MBTL3
Cfa.1
Dre.23.XP_699604.3
Gac.XII
Ola.7
Gga.20
Mdo.1
Cfa.24
Hsa.20.L3MBTL
0.5

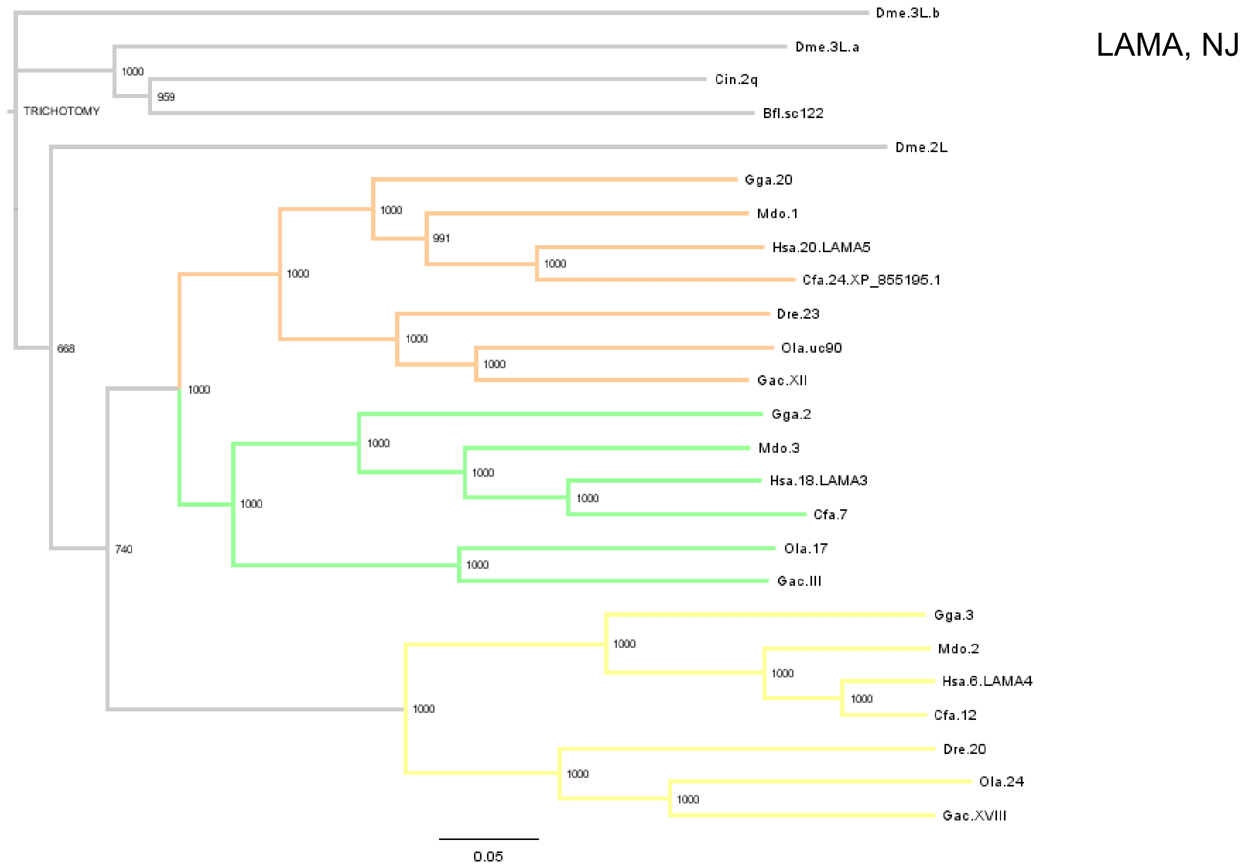
LAMA, NJ
Dme.3L.b
Dme.3L.a
Cin.2q
Bfl.sc122
TRICHOTOMY
Dme.2L
Gga.20
Mdo.1
Hsa.20.LAMA5
Cfa.24.XP_855195.1
Dre.23
Ola.uc90
Gac.XII
Gga.2
Mdo.3
Hsa.18.LAMA3
Cfa.7
Ola.17
Gac.III
Gga.3
Mdo.2
Hsa.6.LAMA4
Cfa.12
Dre.20
Ola.24
Gac.XVIII
1000
959
668
991
740
0.05

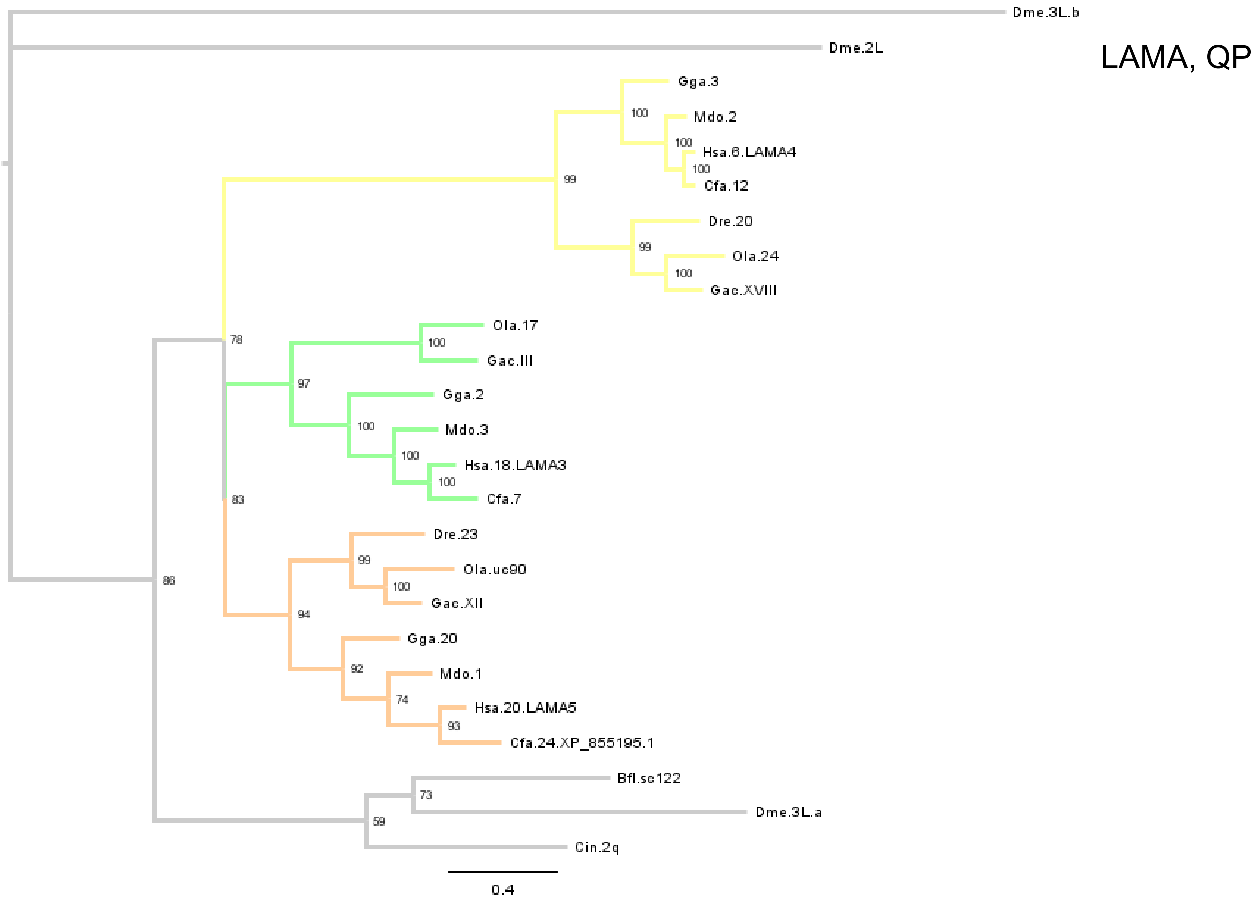
LAMA, QP
Dme.3L.b
Dme.2L
Gga.3
Mdo.2
Hsa.6.LAMA4
Cfa.12
Dre.20
Ola.24
Gac.XVIII
Ola.17
Gac.III
Gga.2
Mdo.3
Hsa.18.LAMA3
Cfa.7
Dre.23
Ola.uc90
Gac.XII
Gga.20
Mdo.1
Hsa.20.LAMA5
Cfa.24.XP_855195.1
Bfl.sc122
Dme.3L.a
Cin.2q
100
99
78
97
83
94
92
74
93
86
73
59
0.4

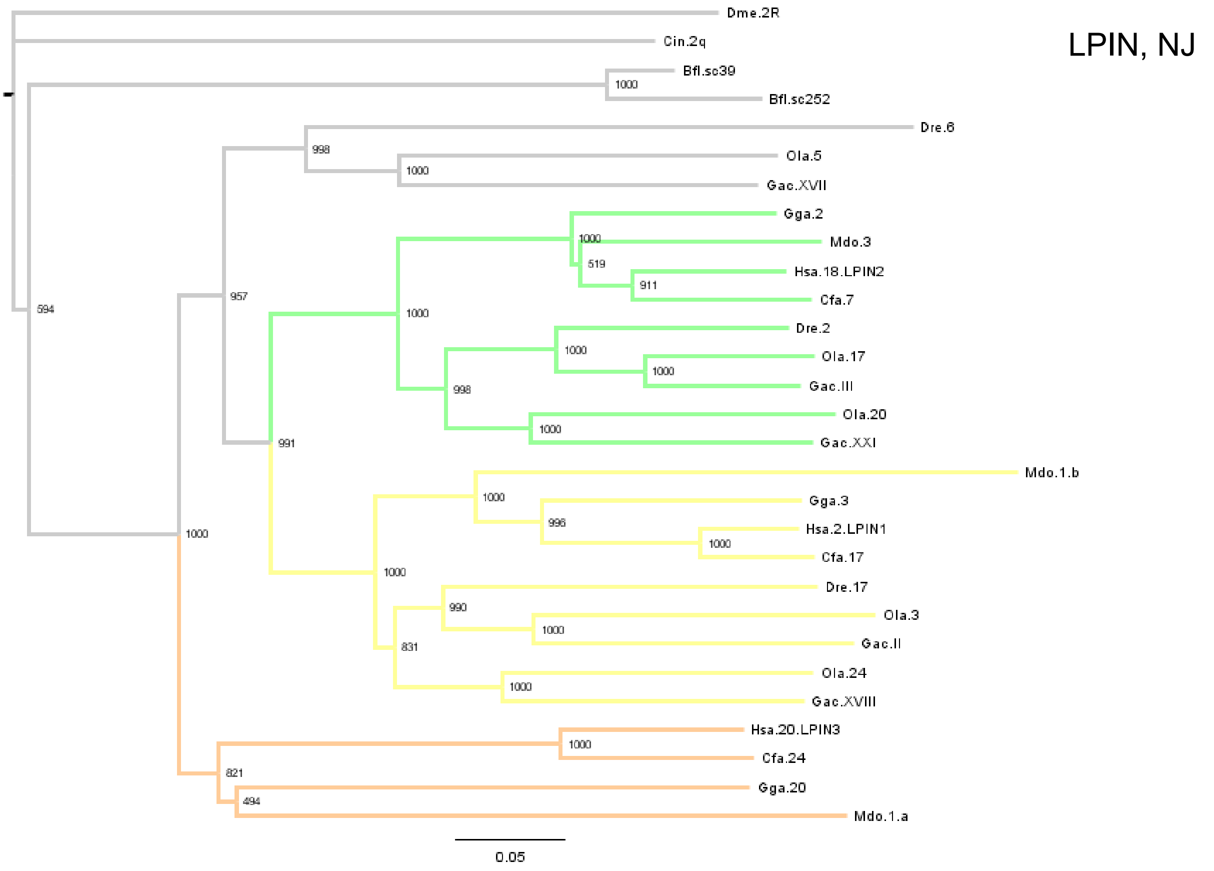
LPIN, NJ
Dme.2R
Cin.2q
Bfl.sc39
Bfl.sc252
Dre.6
Ola.5
Gac.XVII
Gga.2
Mdo.3
Hsa.18.LPIN2
Cfa.7
Dre.2
Ola.17
Gac.III
Ola.20
Gac.XXI
Mdo.1.b
Gga.3
Hsa.2.LPIN1
Cfa.17
Dre.17
Ola.3
Gac.II
Ola.24
Gac.XVIII
Hsa.20.LPIN3
Cfa.24
Gga.20
Mdo.1.a
0.05

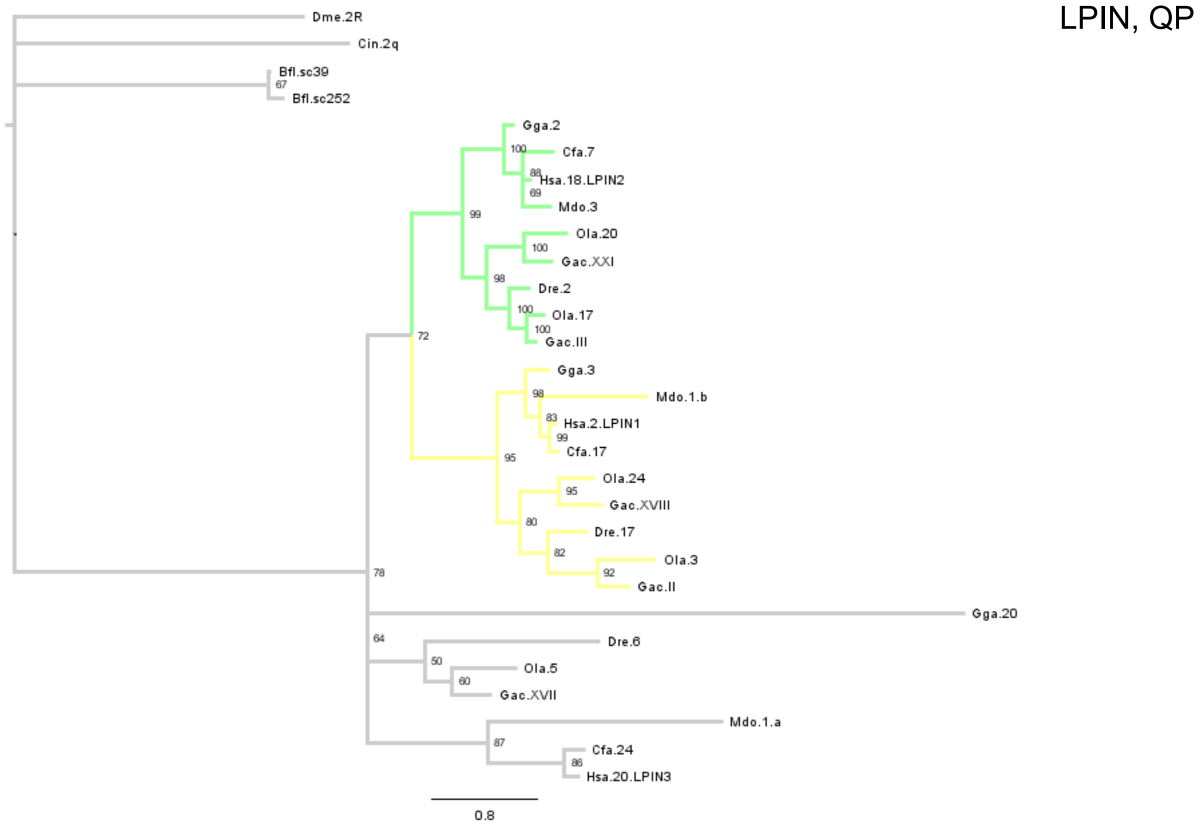
LPIN, QP
Dme.2R
Cin.2q
Bfl.sc39
Bfl.sc252
Gga.2
Cfa.7
Hsa.18.LPIN2
Mdo.3
Ola.20
Gac.XXI
Dre.2
Ola.17
Gac.III
Gga.3
Mdo.1.b
Hsa.2.LPIN1
Cfa.17
Ola.24
Gac.XVIII
Dre.17
Ola.3
Gac.II
Gga.20
Dre.6
Ola.5
Gac.XVII
Mdo.1.a
Cfa.24
Hsa.20.LPIN3
0.8

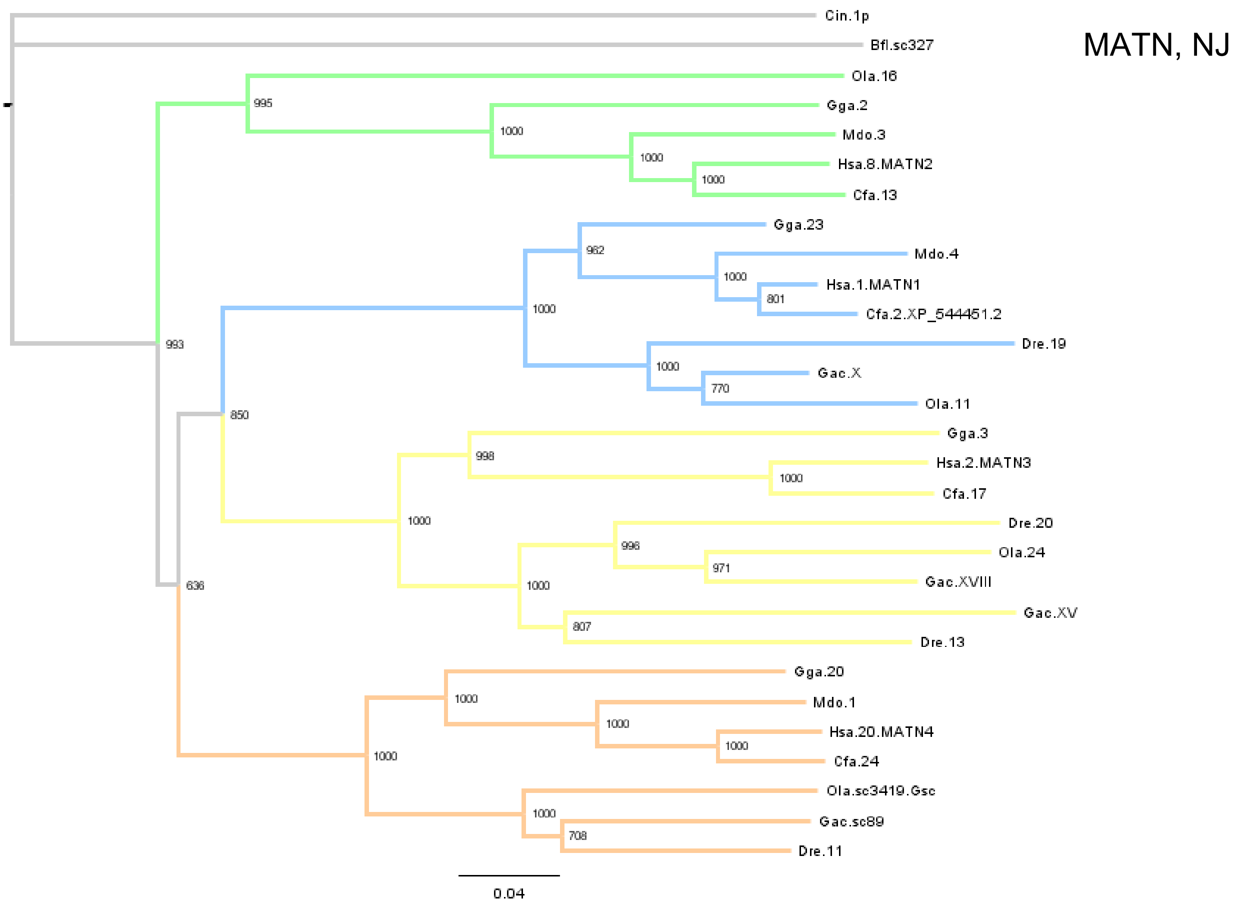
MATN, NJ
Cin.1p
Bfl.sc327
Ola.16
Gga.2
Mdo.3
Hsa.8.MATN2
Cfa.13
Gga.23
Mdo.4
Hsa.1.MATN1
Cfa.2.XP_544451.2
Dre.19
Gac.X
Ola.11
Gga.3
Hsa.2.MATN3
Cfa.17
Dre.20
Ola.24
Gac.XVIII
Gac.XV
Dre.13
Gga.20
Mdo.1
Hsa.20.MATN4
Cfa.24
Ola.sc3419.Gsc
Gac.sc89
Dre.11
995
1000
1000
1000
962
1000
801
1000
1000
770
993
850
998
1000
1000
996
971
1000
807
636
1000
1000
1000
1000
1000
1000
708
0.04

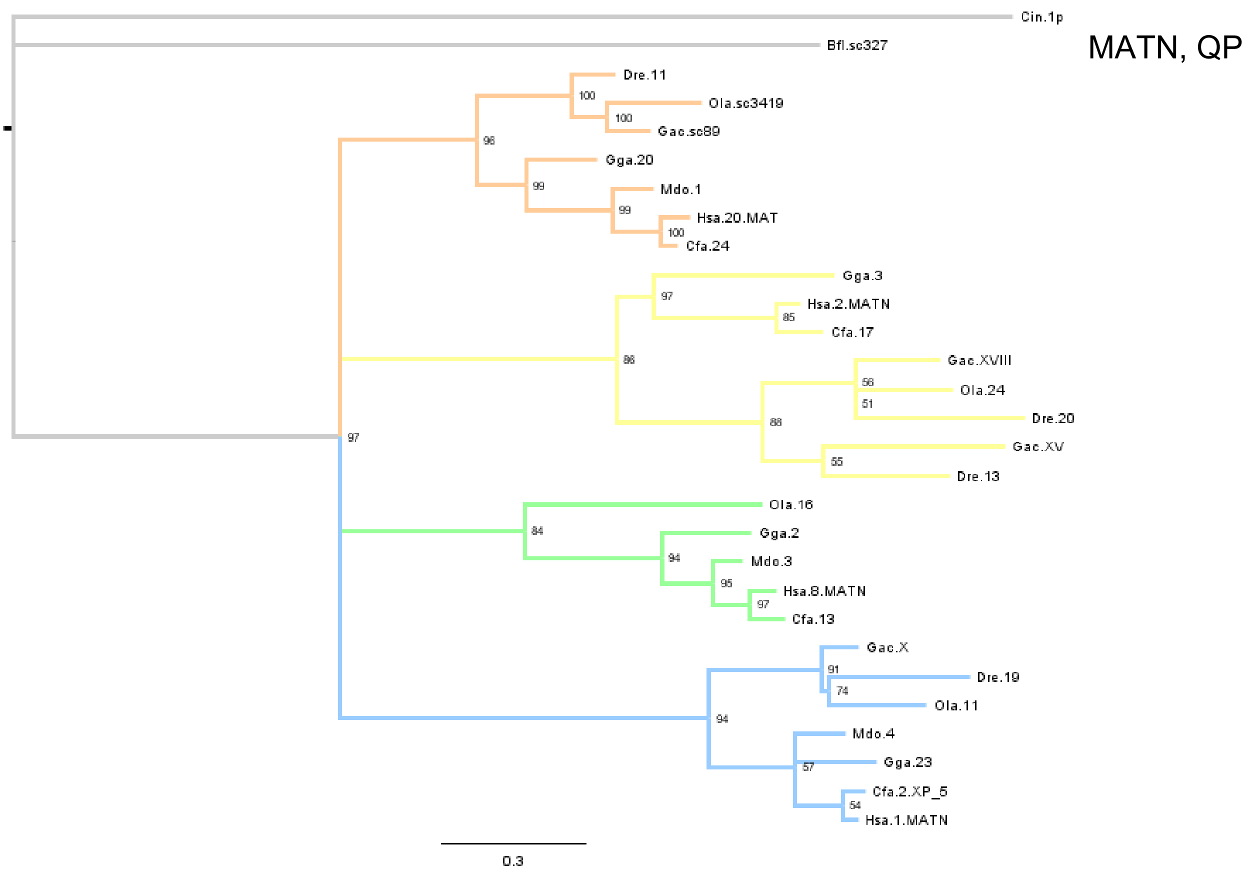
MATN, QP
Cin.1p
Bfl.sc327
Dre.11
Ola.sc3419
Gac.sc89
Gga.20
Mdo.1
Hsa.20.MAT
Cfa.24
Gga.3
Hsa.2.MATN
Cfa.17
Gac.XVIII
Ola.24
Dre.20
Gac.XV
Dre.13
Ola.16
Gga.2
Mdo.3
Hsa.8.MATN
Cfa.13
Gac.X
Dre.19
Ola.11
Mdo.4
Gga.23
Cfa.2.XP_5
Hsa.1.MATN
100
100
96
99
99
100
97
85
86
56
51
88
55
97
84
94
95
97
91
74
94
57
54
0.3

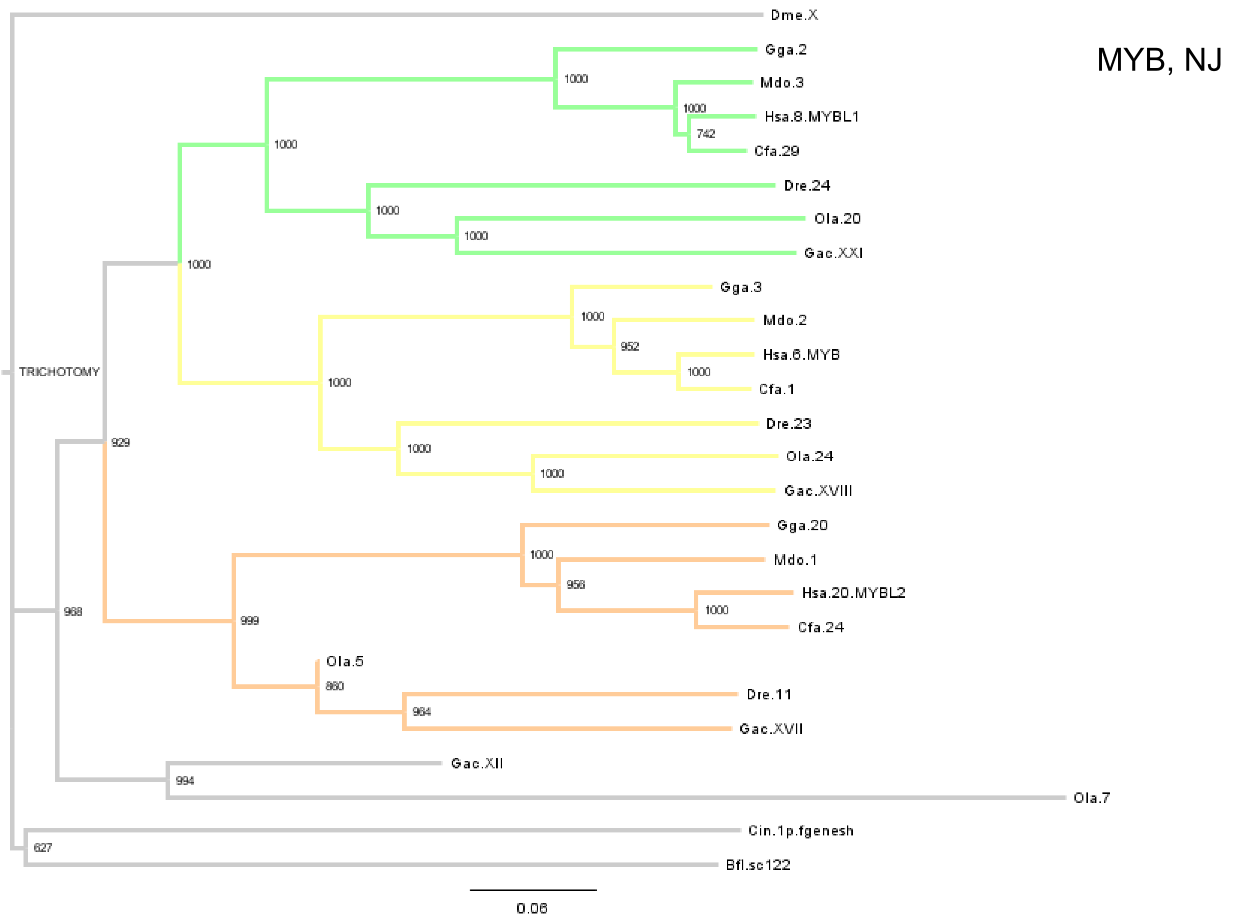

MYB, NJ
Dme.X
Gga.2
Mdo.3
Hsa.8.MYBL1
Cfa.29
Dre.24
Ola.20
Gac.XXI
Gga.3
Mdo.2
Hsa.6.MYB
Cfa.1
Dre.23
Ola.24
Gac.XVIII
Gga.20
Mdo.1
Hsa.20.MYBL2
Cfa.24
Ola.5
Dre.11
Gac.XVII
Gac.XII
Ola.7
Cin.1p.fgenesh
Bfl.sc122
TRICHOTOMY
0.06

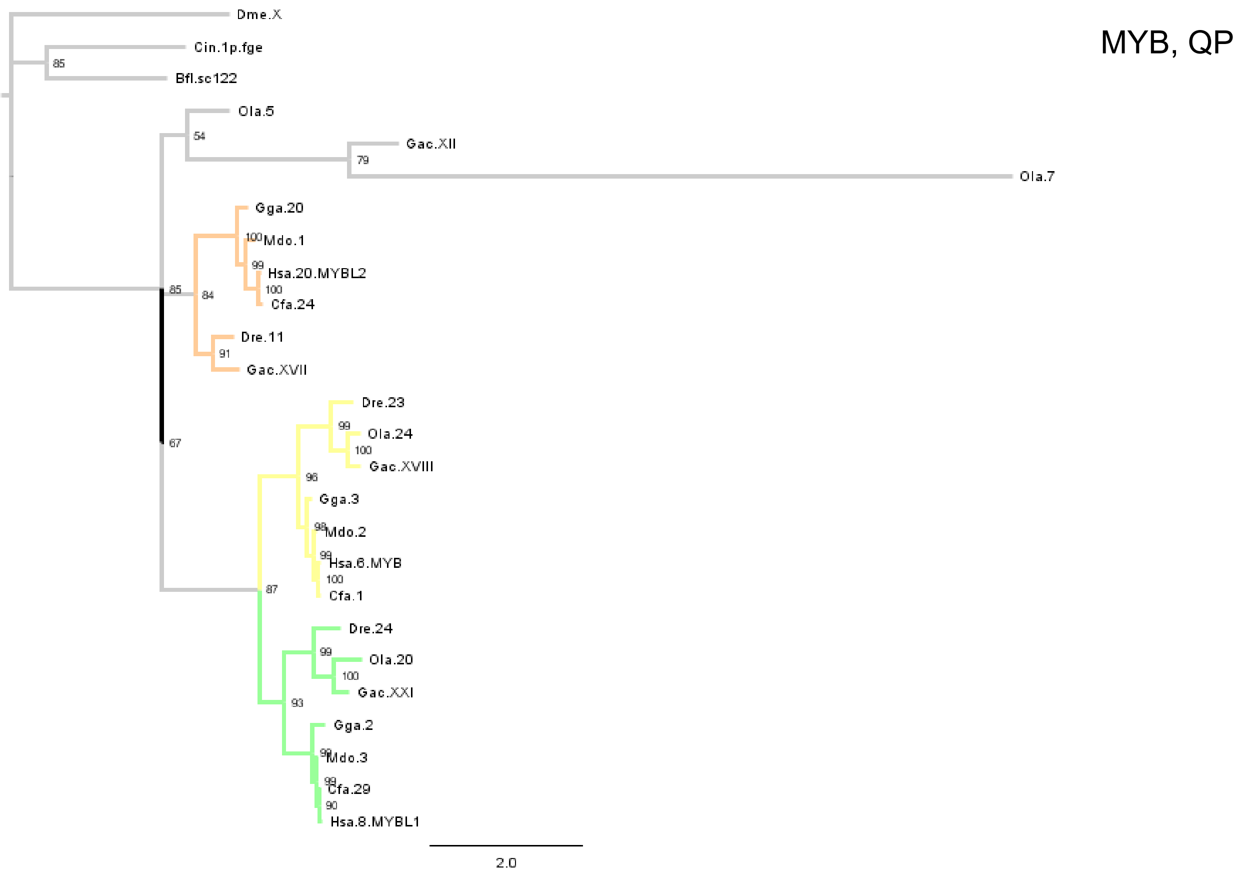

MYB, QP
Dme.X
Cin.1p.fge
Bfl.sc122
Ola.5
Gac.XII
Ola.7
Gga.20
Mdo.1
Hsa.20.MYBL2
Cfa.24
Dre.11
Gac.XVII
Dre.23
Ola.24
Gac.XVIII
Gga.3
Mdo.2
Hsa.6.MYB
Cfa.1
Dre.24
Ola.20
Gac.XXI
Gga.2
Mdo.3
Cfa.29
Hsa.8.MYBL1
2.0

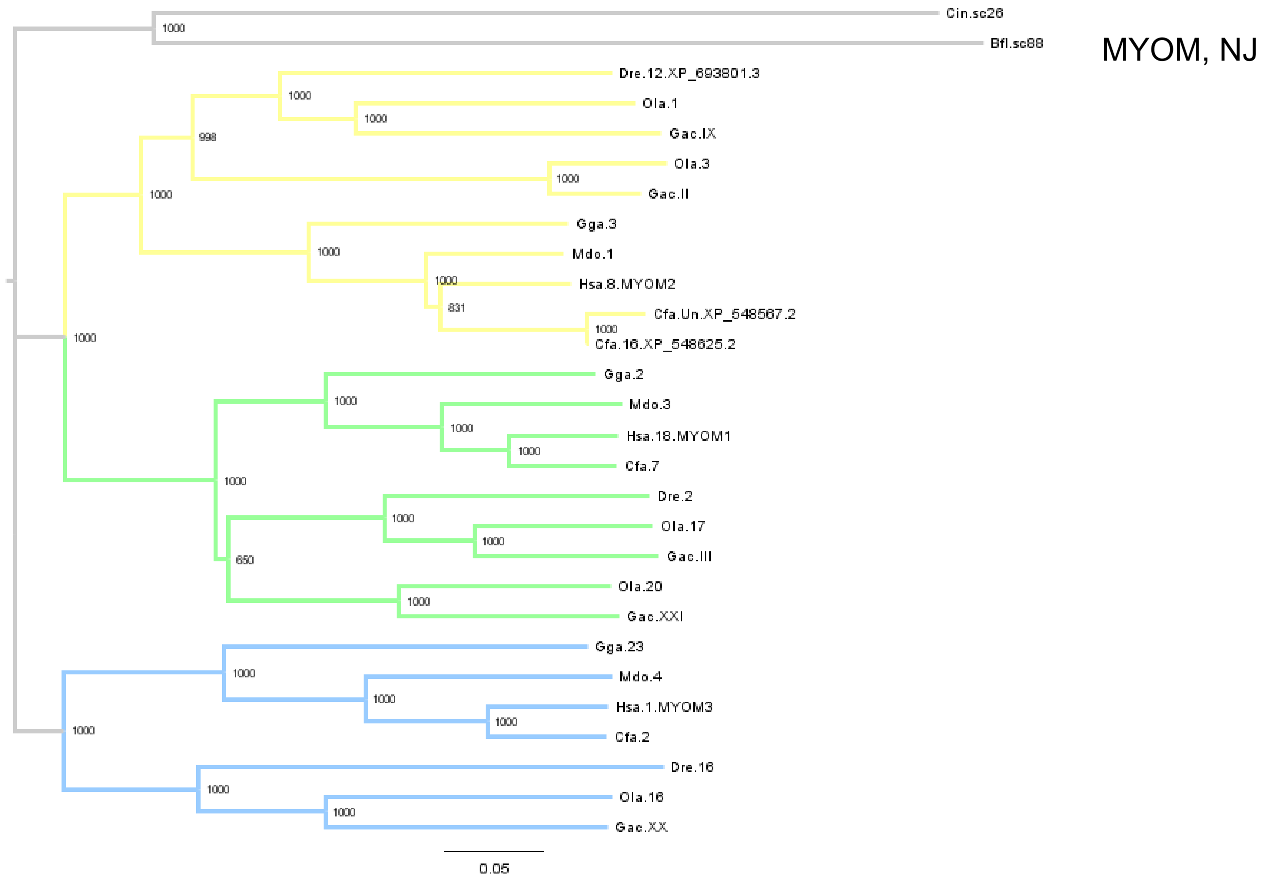
MYOM, NJ
Cin.sc26
Bfl.sc88
Dre.12.XP_693801.3
Ola.1
Gac.IX
Ola.3
Gac.II
Gga.3
Mdo.1
Hsa.8.MYOM2
Cfa.Un.XP_548567.2
Cfa.16.XP_548625.2
Gga.2
Mdo.3
Hsa.18.MYOM1
Cfa.7
Dre.2
Ola.17
Gac.III
Ola.20
Gac.XXI
Gga.23
Mdo.4
Hsa.1.MYOM3
Cfa.2
Dre.16
Ola.16
Gac.XX
0.05

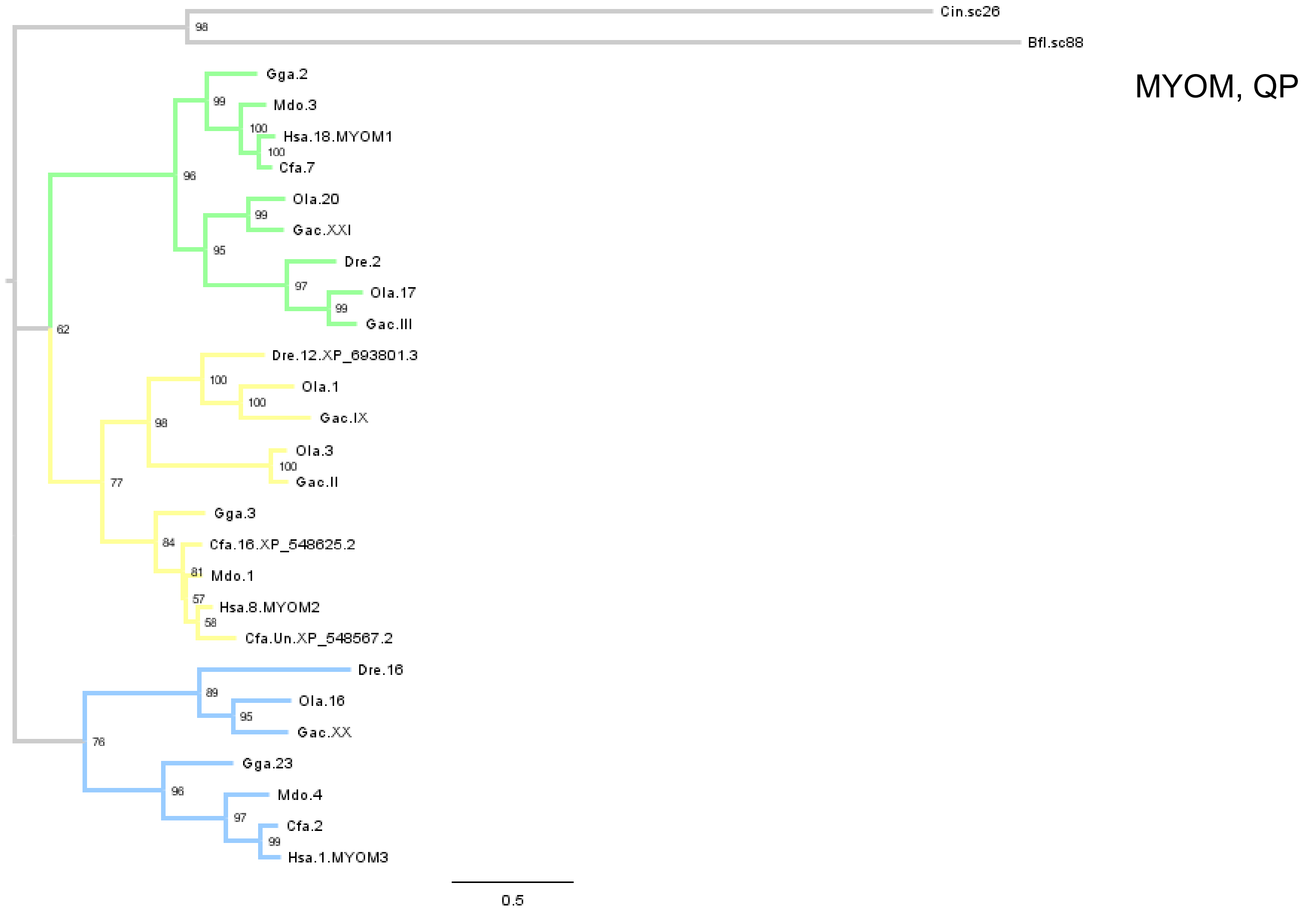
MYOM, QP
Cin.sc26
Bfl.sc88
Gga.2
Mdo.3
Hsa.18.MYOM1
Cfa.7
Ola.20
Gac.XXI
Dre.2
Ola.17
Gac.III
Dre.12.XP_693801.3
Ola.1
Gac.IX
Ola.3
Gac.II
Gga.3
Cfa.16.XP_548625.2
Mdo.1
Hsa.8.MYOM2
Cfa.Un.XP_548567.2
Dre.16
Ola.16
Gac.XX
Gga.23
Mdo.4
Cfa.2
Hsa.1.MYOM3
0.5

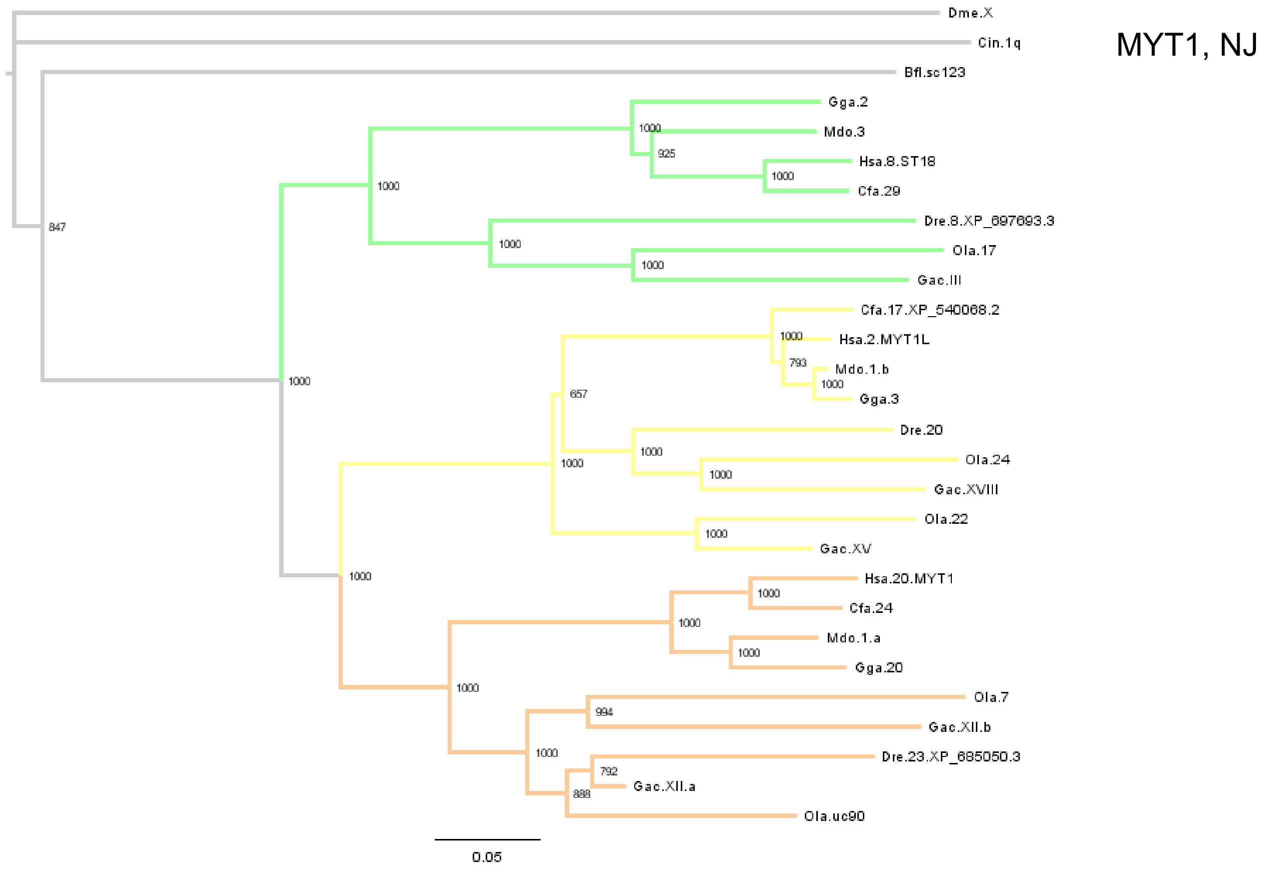

MYT1, NJ
Dme.X
Cin.1q
Bfl.sc123
Gga.2
Mdo.3
Hsa.8.ST18
Cfa.29
Dre.8.XP_697693.3
Ola.17
Gac.III
Cfa.17.XP_540068.2
Hsa.2.MYT1L
Mdo.1.b
Gga.3
Dre.20
Ola.24
Gac.XVIII
Ola.22
Gac.XV
Hsa.20.MYT1
Cfa.24
Mdo.1.a
Gga.20
Ola.7
Gac.XII.b
Dre.23.XP_685050.3
Gac.XII.a
Ola.uc90
0.05

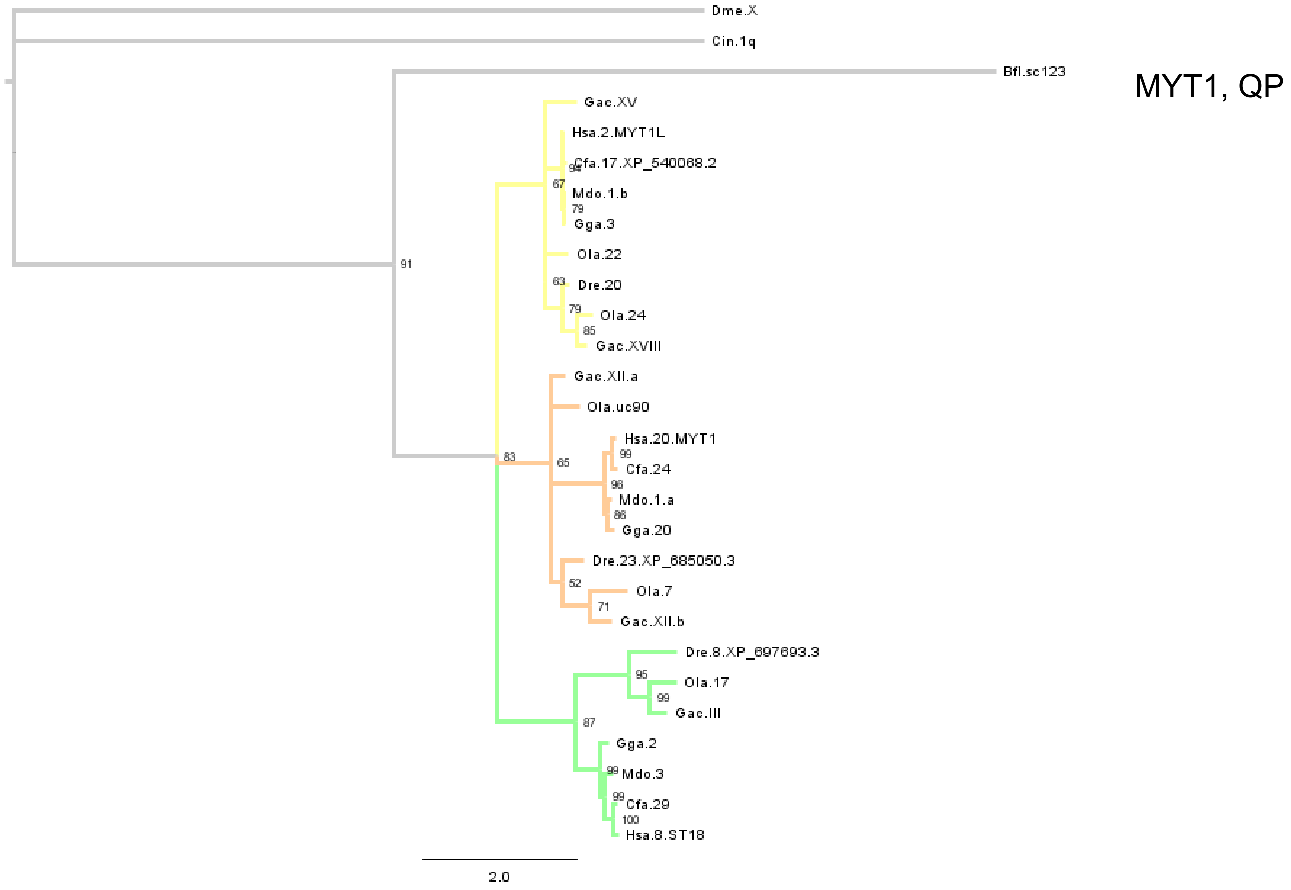

MYT1, QP
Dme.X
Cin.1q
Bfl.sc123
Gac.XV
Hsa.2.MYT1L
Cfa.17.XP_540068.2
Mdo.1.b
Gga.3
Ola.22
Dre.20
Ola.24
Gac.XVIII
Gac.XII.a
Ola.uc90
Hsa.20.MYT1
Cfa.24
Mdo.1.a
Gga.20
Dre.23.XP_685050.3
Ola.7
Gac.XII.b
Dre.8.XP_697693.3
Ola.17
Gac.III
Gga.2
Mdo.3
Cfa.29
Hsa.8.ST18
2.0

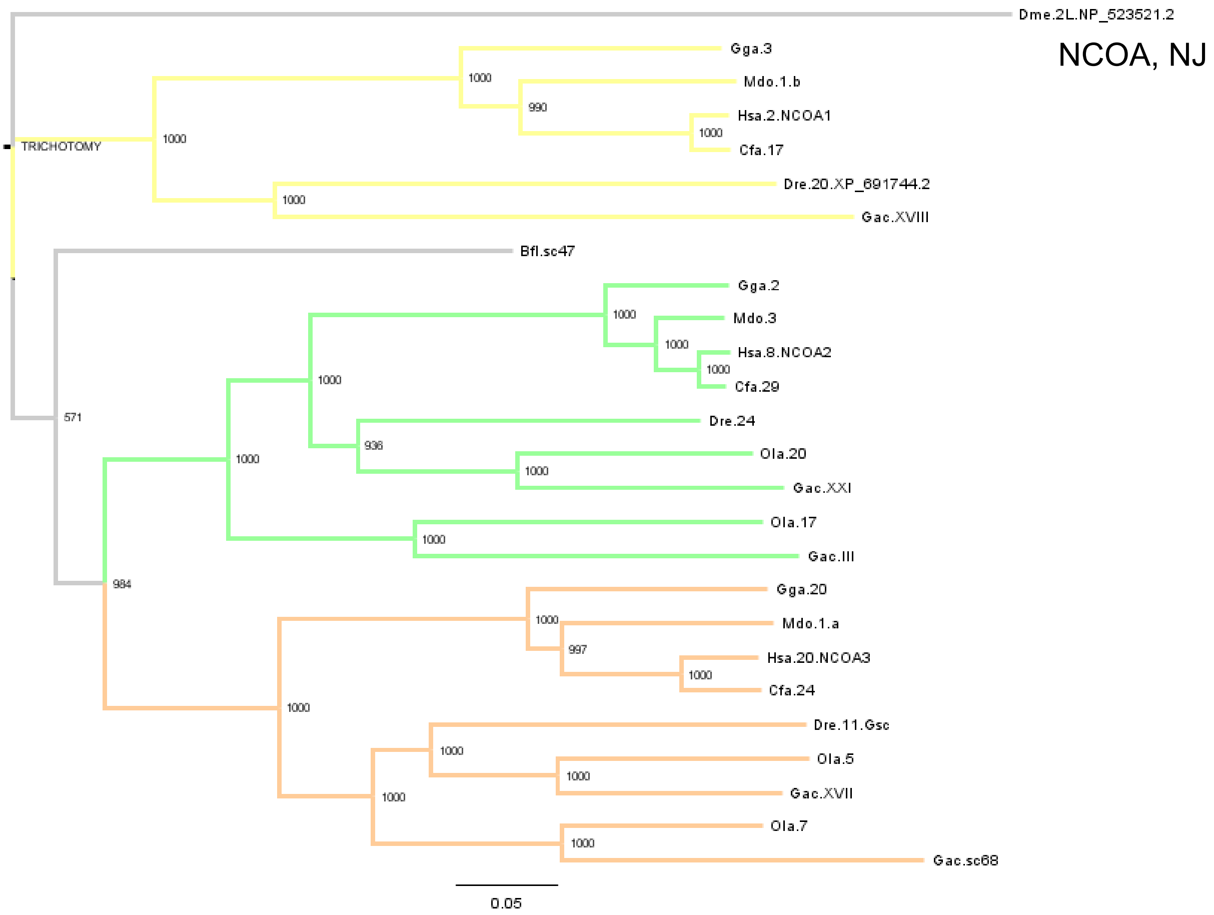

NCOA, NJ
Dme.2L.NP_523521.2
Gga.3
Mdo.1.b
Hsa.2.NCOA1
Cfa.17
Dre.20.XP_691744.2
Gac.XVIII
TRICHOTOMY
Bfl.sc47
Gga.2
Mdo.3
Hsa.8.NCOA2
Cfa.29
Dre.24
Ola.20
Gac.XXI
Ola.17
Gac.III
Gga.20
Mdo.1.a
Hsa.20.NCOA3
Cfa.24
Dre.11.Gsc
Ola.5
Gac.XVII
Ola.7
Gac.sc68
571
984
0.05

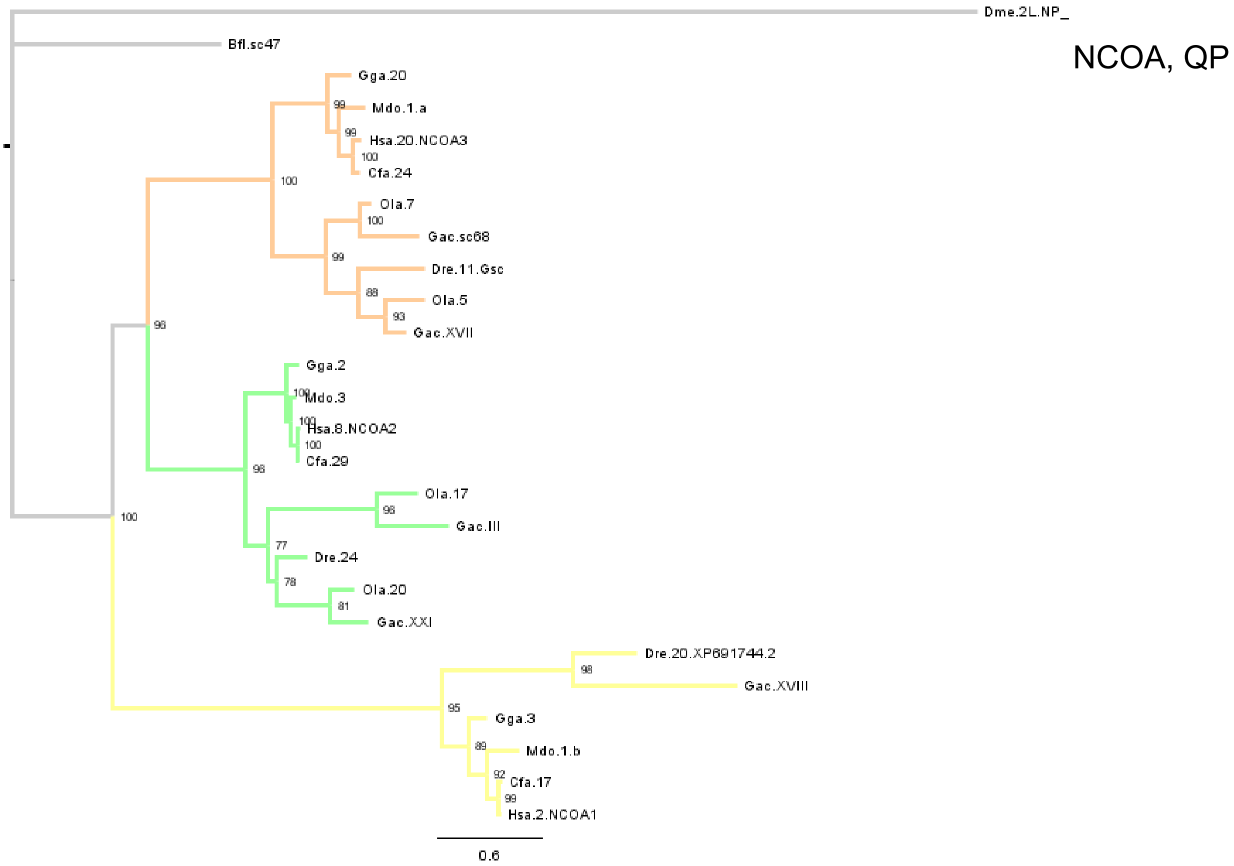

NCOA, QP
Dme.2L.NP_
Bfl.sc47
Gga.20
Mdo.1.a
Hsa.20.NCOA3
Cfa.24
Ola.7
Gac.sc68
Dre.11.Gsc
Ola.5
Gac.XVII
Gga.2
Mdo.3
Hsa.8.NCOA2
Cfa.29
Ola.17
Gac.III
Dre.24
Ola.20
Gac.XXI
Dre.20.XP691744.2
Gac.XVIII
Gga.3
Mdo.1.b
Cfa.17
Hsa.2.NCOA1
0.6

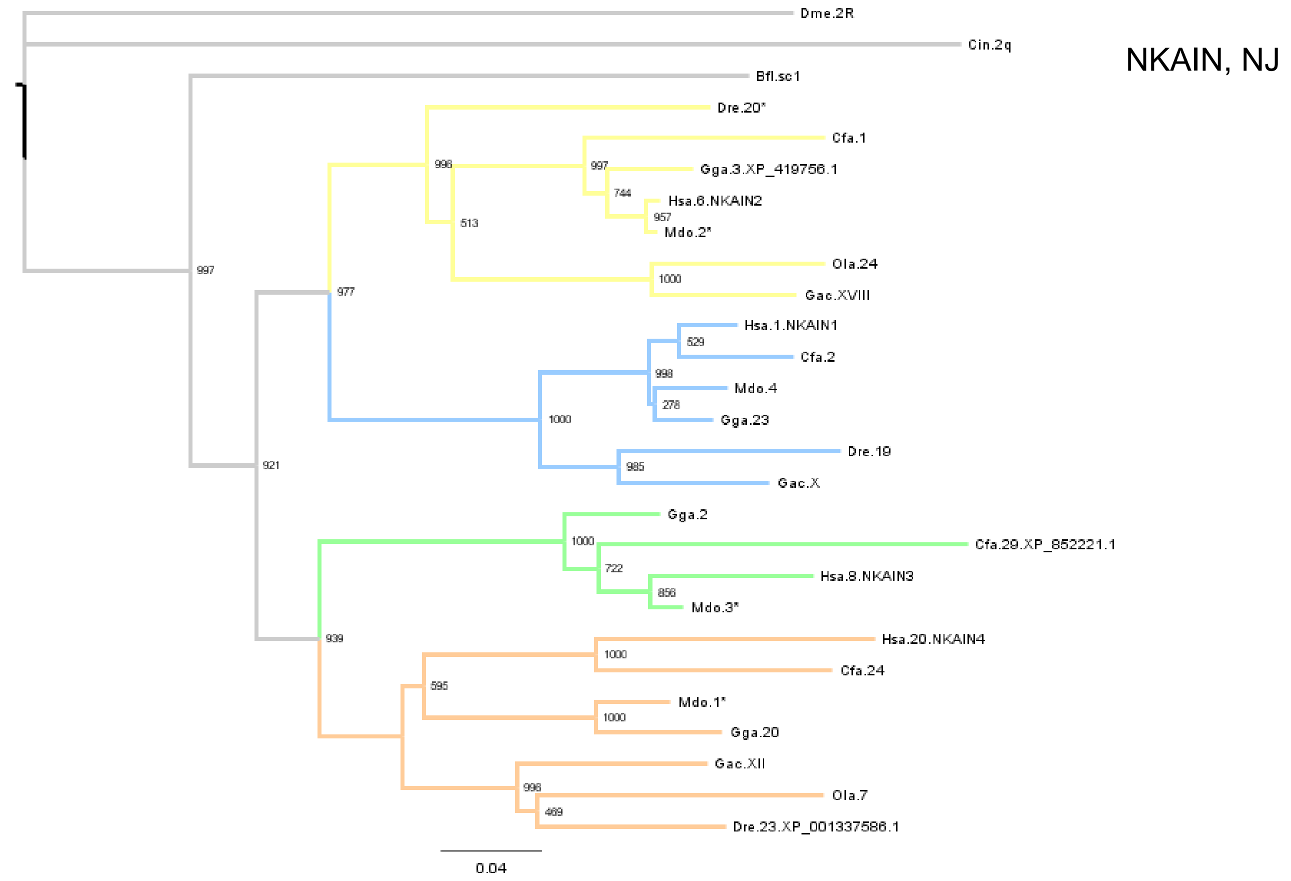
NKAIN, NJ
Dme.2R
Cin.2q
Bfl.sc1
Dre.20*
Cfa.1
Gga.3.XP_419756.1
Hsa.6.NKAIN2
Mdo.2*
Ola.24
Gac.XVIII
Hsa.1.NKAIN1
Cfa.2
Mdo.4
Gga.23
Dre.19
Gac.X
Gga.2
Cfa.29.XP_852221.1
Hsa.8.NKAIN3
Mdo.3*
Hsa.20.NKAIN4
Cfa.24
Mdo.1*
Gga.20
Gac.XII
Ola.7
Dre.23.XP_001337586.1
0.04

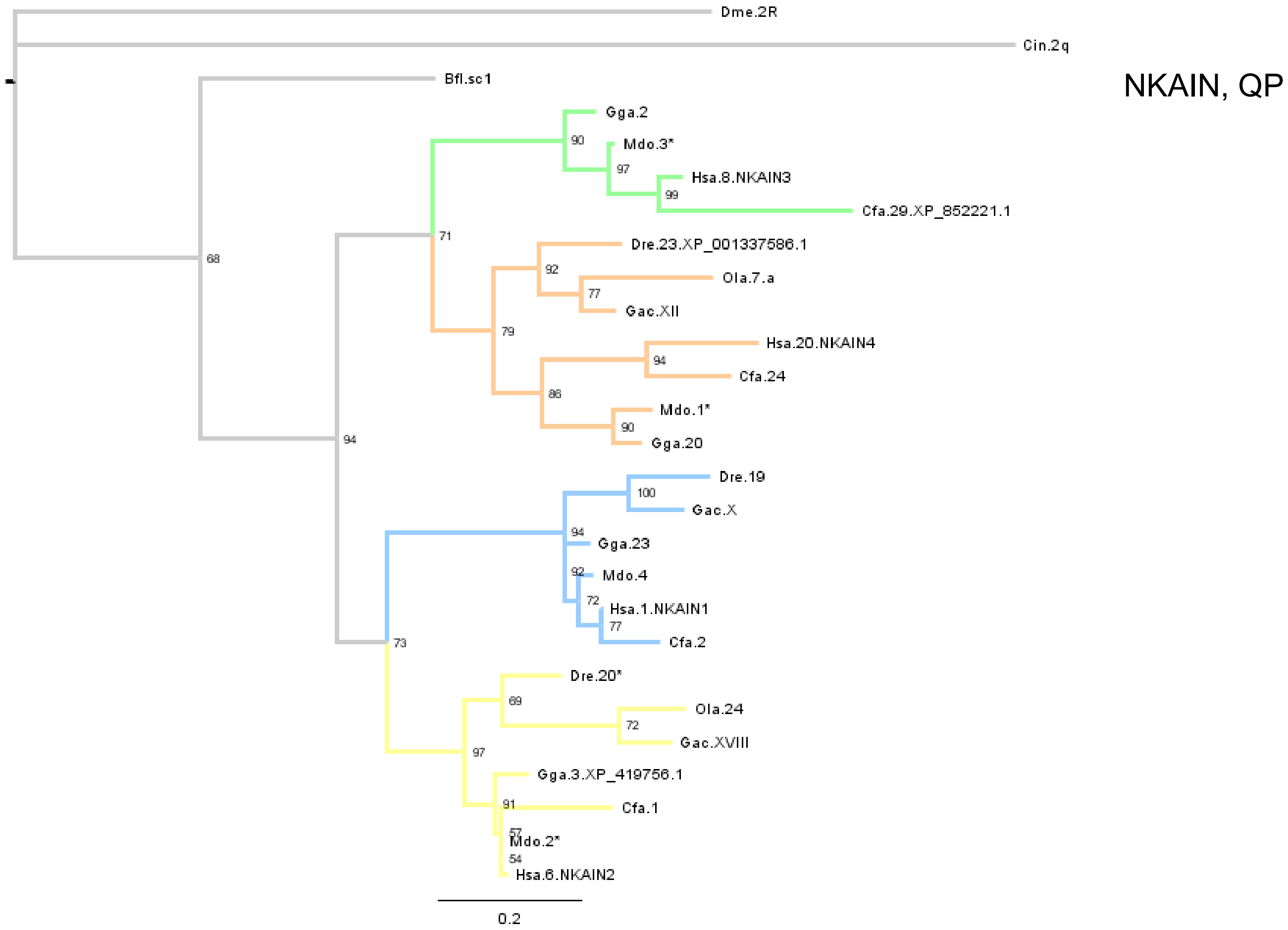
NKAIN, QP
Dme.2R
Cin.2q
Bfl.sc1
Gga.2
Mdo.3*
Hsa.8.NKAIN3
Cfa.29.XP_852221.1
Dre.23.XP_001337586.1
Ola.7.a
Gac.XII
Hsa.20.NKAIN4
Cfa.24
Mdo.1*
Gga.20
Dre.19
Gac.X
Gga.23
Mdo.4
Hsa.1.NKAIN1
Cfa.2
Dre.20*
Ola.24
Gac.XVIII
Gga.3.XP_419756.1
Cfa.1
Mdo.2*
Hsa.6.NKAIN2
0.2

OPIOID, NJ

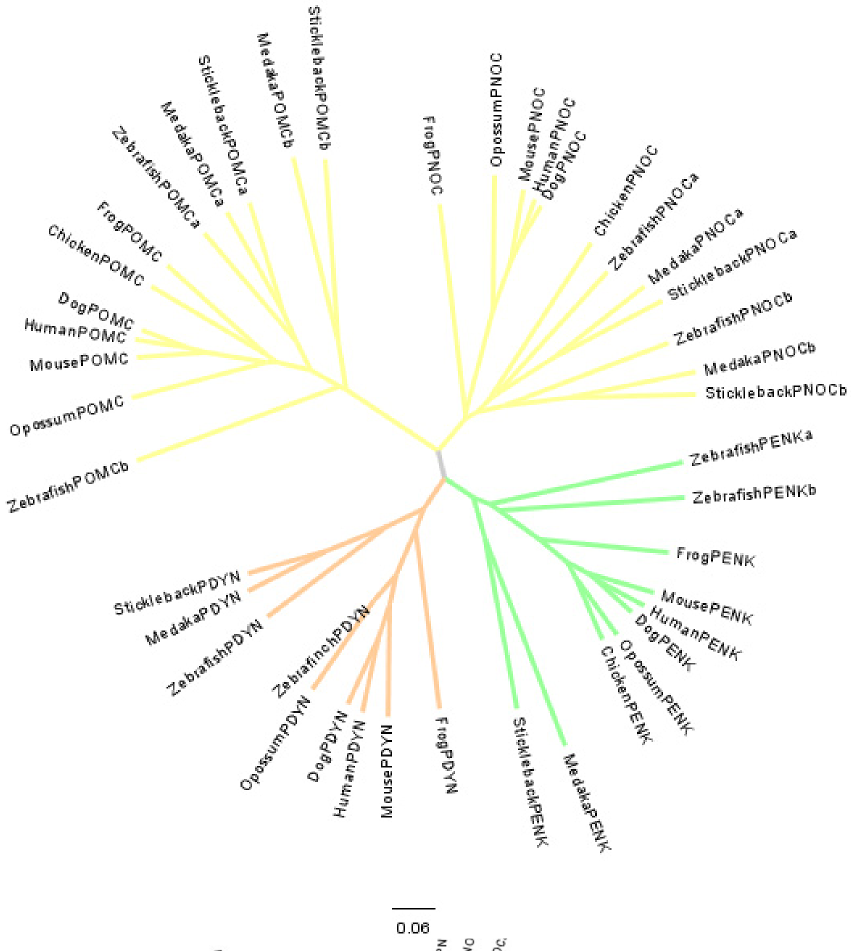

OPIOID, QP

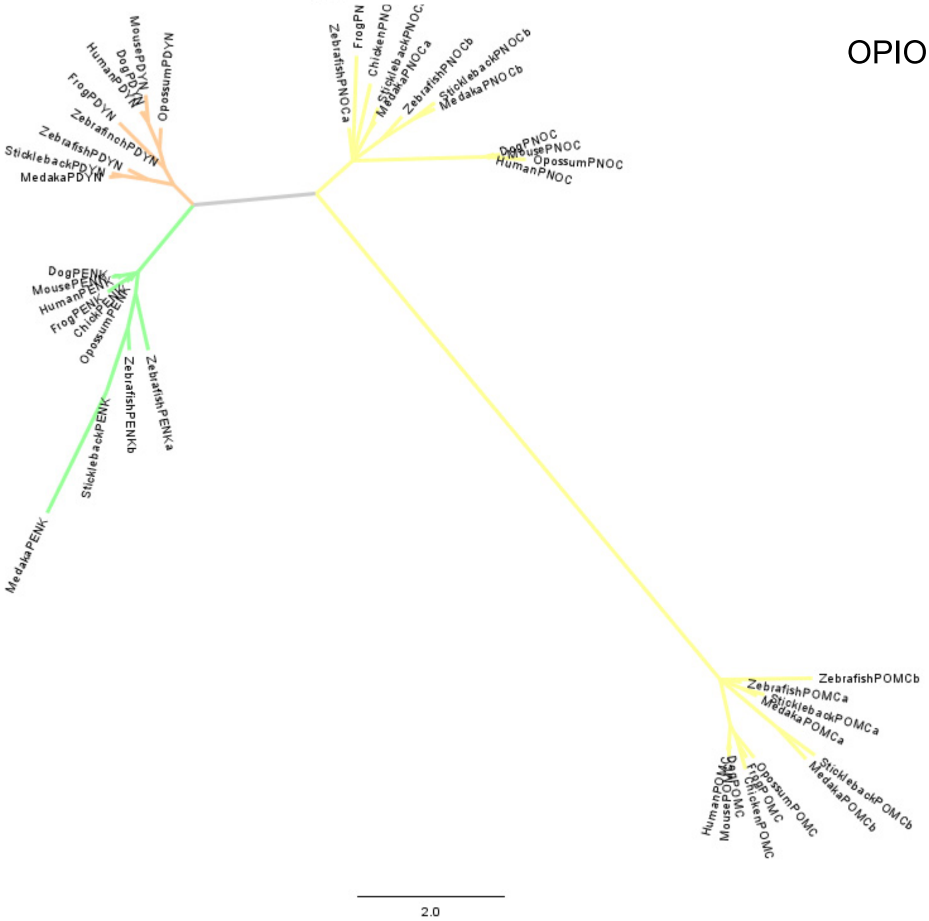

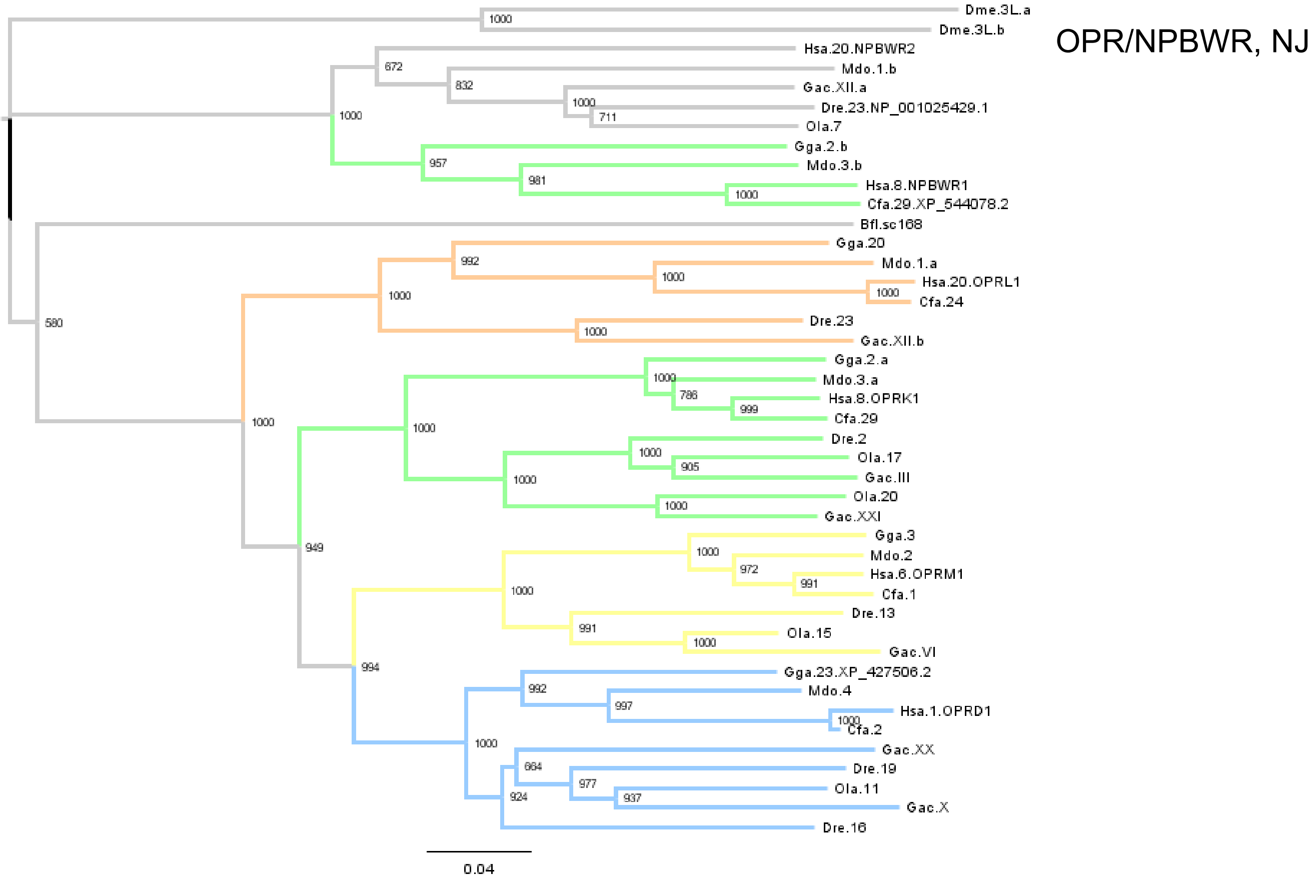
OPR/NPBWR, NJ
Dme.3L.a
Dme.3L.b
Hsa.20.NPBWR2
Mdo.1.b
Gac.XII.a
Dre.23.NP_001025429.1
Ola.7
Gga.2.b
Mdo.3.b
Hsa.8.NPBWR1
Cfa.29.XP_544078.2
Bfl.sc168
Gga.20
Mdo.1.a
Hsa.20.OPRL1
Cfa.24
Dre.23
Gac.XII.b
Gga.2.a
Mdo.3.a
Hsa.8.OPRK1
Cfa.29
Dre.2
Ola.17
Gac.III
Ola.20
Gac.XXI
Gga.3
Mdo.2
Hsa.6.OPRM1
Cfa.1
Dre.13
Ola.15
Gac.VI
Gga.23.XP_427506.2
Mdo.4
Hsa.1.OPRD1
Cfa.2
Gac.XX
Dre.19
Ola.11
Gac.X
Dre.16
1000
672
832
1000
711
1000
957
981
1000
580
992
1000
1000
1000
1000
1000
1000
786
999
1000
1000
1000
905
1000
949
1000
1000
972
991
991
1000
994
992
997
1000
1000
664
977
924
937
0.04

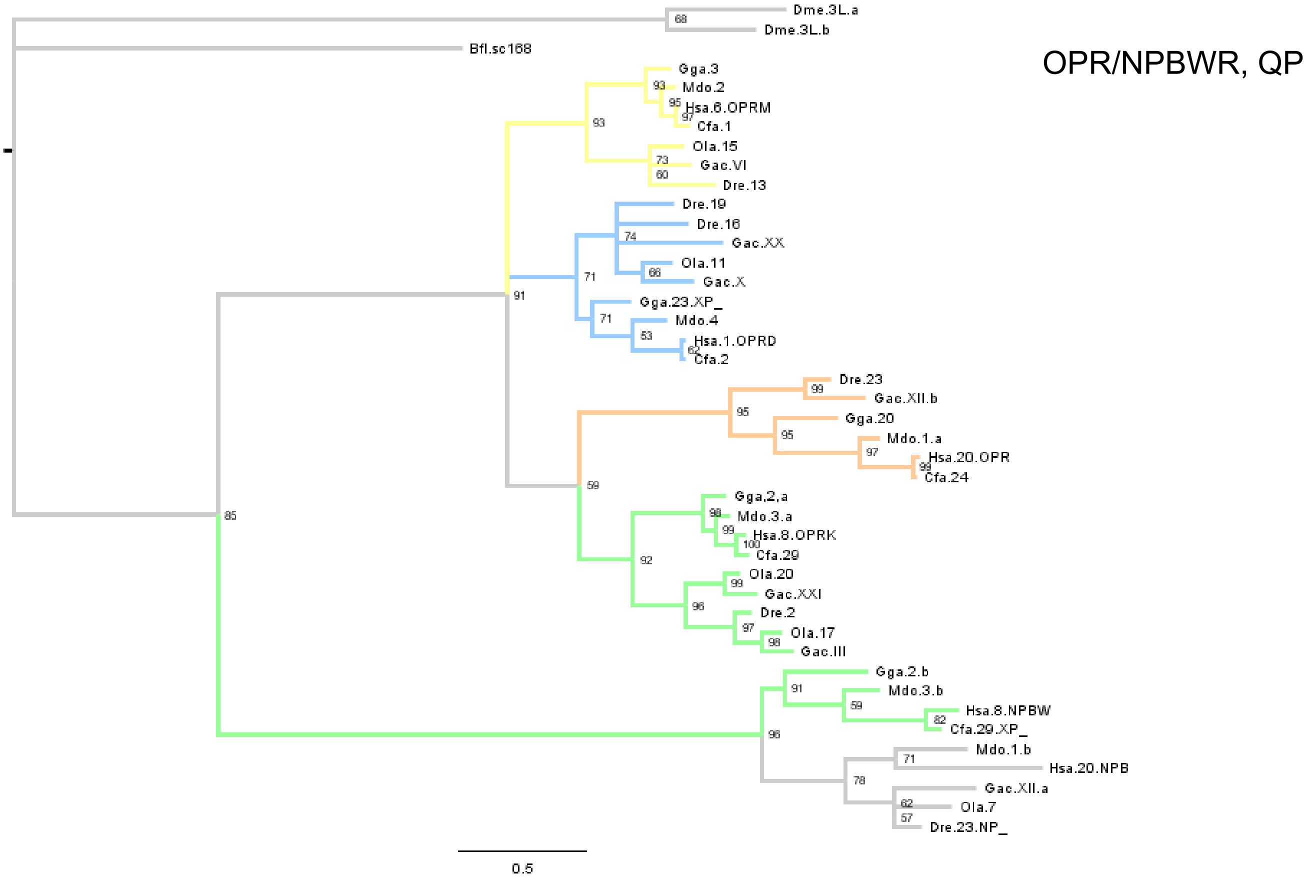
OPR/NPBWR, QP
Dme.3L.a
Dme.3L.b
Bfl.sc168
Gga.3
Mdo.2
Hsa.6.OPRM
Cfa.1
Ola.15
Gac.VI
Dre.13
Dre.19
Dre.16
Gac.XX
Ola.11
Gac.X
Gga.23.XP_
Mdo.4
Hsa.1.OPRD
Cfa.2
Dre.23
Gac.XII.b
Gga.20
Mdo.1.a
Hsa.20.OPR
Cfa.24
Gga,2,a
Mdo.3.a
Hsa.8.OPRK
Cfa.29
Ola.20
Gac.XXI
Dre.2
Ola.17
Gac.III
Gga.2.b
Mdo.3.b
Hsa.8.NPBW
Cfa.29.XP_
Mdo.1.b
Hsa.20.NPB
Gac.XII.a
Ola.7
Dre.23.NP_
68
93
95
97
93
73
60
74
66
71
71
53
62
91
99
95
95
97
99
59
98
99
100
92
99
96
97
98
85
91
59
82
96
71
78
62
57
0.5

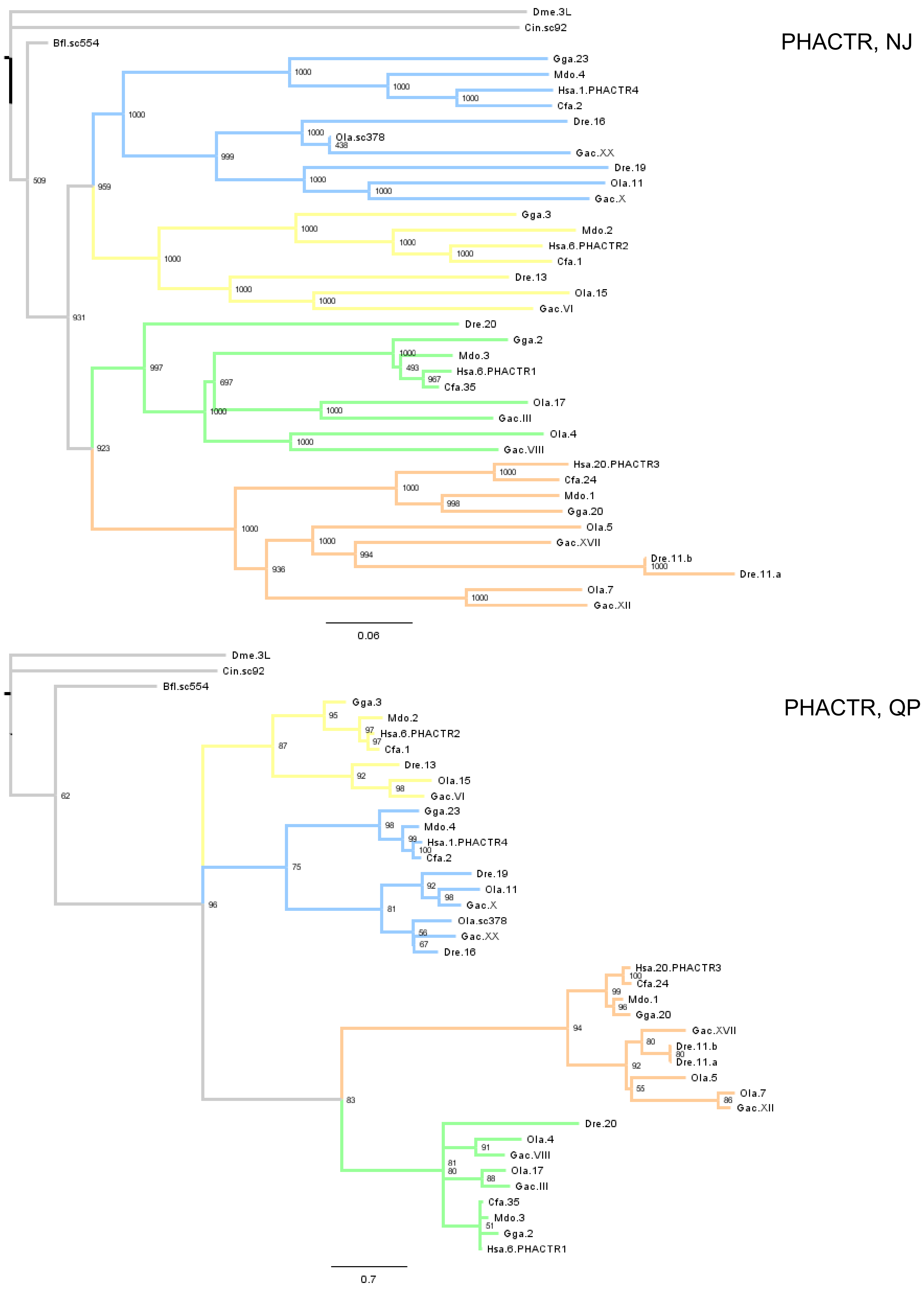
PHACTR, NJ
Dme.3L
Cin.sc92
Bfl.sc554
Gga.23
Mdo.4
Hsa.1.PHACTR4
Cfa.2
Dre.16
Ola.sc378
Gac.XX
Dre.19
Ola.11
Gac.X
Gga.3
Mdo.2
Hsa.6.PHACTR2
Cfa.1
Dre.13
Ola.15
Gac.VI
Dre.20
Gga.2
Mdo.3
Hsa.6.PHACTR1
Cfa.35
Ola.17
Gac.III
Ola.4
Gac.VIII
Hsa.20.PHACTR3
Cfa.24
Mdo.1
Gga.20
Ola.5
Gac.XVII
Dre.11.b
Dre.11.a
Ola.7
Gac.XII
0.06
PHACTR, QP
Dme.3L
Cin.sc92
Bfl.sc554
Gga.3
Mdo.2
Hsa.6.PHACTR2
Cfa.1
Dre.13
Ola.15
Gac.VI
Gga.23
Mdo.4
Hsa.1.PHACTR4
Cfa.2
Dre.19
Ola.11
Gac.X
Ola.sc378
Gac.XX
Dre.16
Hsa.20.PHACTR3
Cfa.24
Mdo.1
Gga.20
Gac.XVII
Dre.11.b
Dre.11.a
Ola.5
Ola.7
Gac.XII
Dre.20
Ola.4
Gac.VIII
Ola.17
Gac.III
Cfa.35
Mdo.3
Gga.2
Hsa.6.PHACTR1
0.7

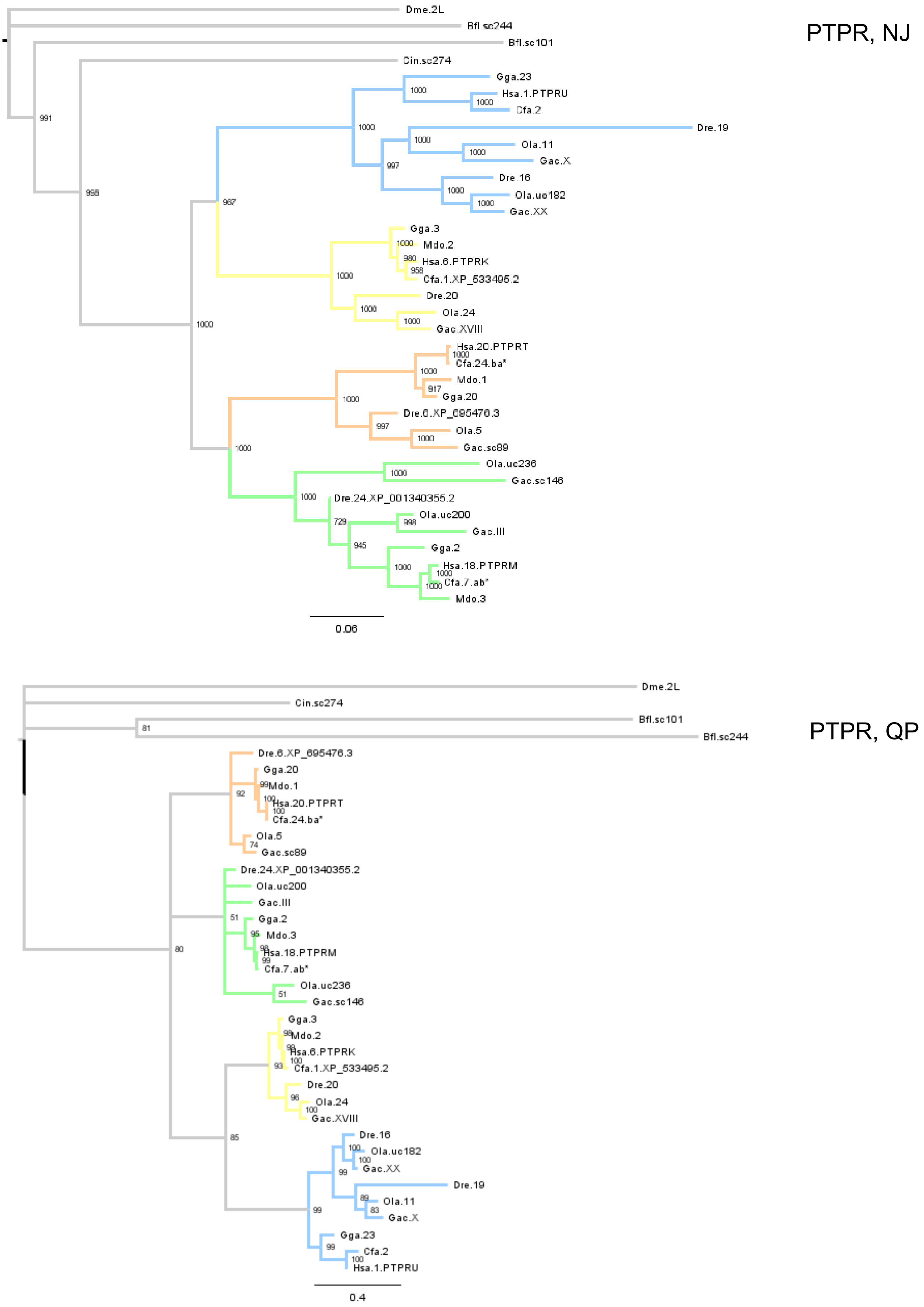
PTPR, NJ
Dme.2L
Bfl.sc244
Bfl.sc101
Cin.sc274
Gga.23
Hsa.1.PTPRU
Cfa.2
Dre.19
Ola.11
Gac.X
Dre.16
Ola.uc182
Gac.XX
Gga.3
Mdo.2
Hsa.6.PTPRK
Cfa.1.XP_533495.2
Dre.20
Ola.24
Gac.XVIII
Hsa.20.PTPRT
Cfa.24.ba*
Mdo.1
Gga.20
Dre.6.XP_695476.3
Ola.5
Gac.sc89
Ola.uc236
Gac.sc146
Dre.24.XP_001340355.2
Ola.uc200
Gac.III
Gga.2
Hsa.18.PTPRM
Cfa.7.ab*
Mdo.3
0.06
PTPR, QP
Dme.2L
Cin.sc274
Bfl.sc101
Bfl.sc244
Dre.6.XP_695476.3
Gga.20
Mdo.1
Hsa.20.PTPRT
Cfa.24.ba*
Ola.5
Gac.sc89
Dre.24.XP_001340355.2
Ola.uc200
Gac.III
Gga.2
Mdo.3
Hsa.18.PTPRM
Cfa.7.ab*
Ola.uc236
Gac.sc146
Gga.3
Mdo.2
Hsa.6.PTPRK
Cfa.1.XP_533495.2
Dre.20
Ola.24
Gac.XVIII
Dre.16
Ola.uc182
Gac.XX
Dre.19
Ola.11
Gac.X
Gga.23
Cfa.2
Hsa.1.PTPRU
0.4

RGS, NJ

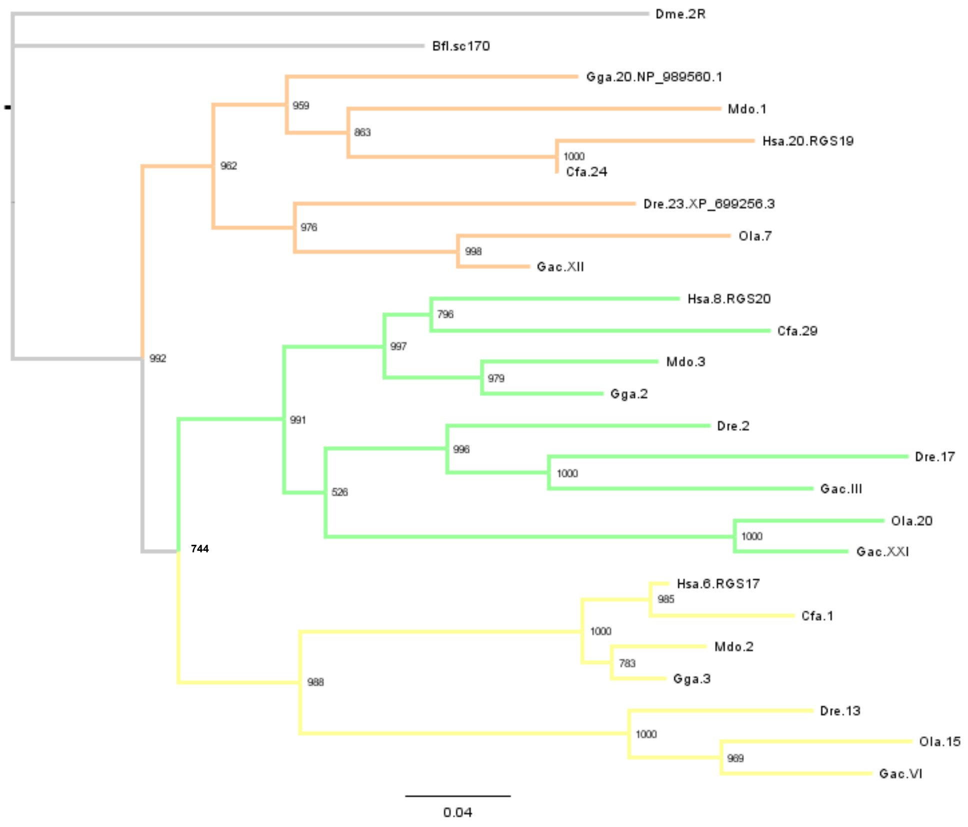
Dme.2R
Bfl.sc170
Gga.20.NP_989560.1
Mdo.1
Hsa.20.RGS19
Cfa.24
Dre.23.XP_699256.3
Ola.7
Gac.XII
Hsa.8.RGS20
Cfa.29
Mdo.3
Gga.2
Dre.2
Dre.17
Gac.III
Ola.20
Gac.XXI
Hsa.6.RGS17
Cfa.1
Mdo.2
Gga.3
Dre.13
Ola.15
Gac.VI
959
863
1000
962
976
998
992
796
997
979
991
996
1000
526
1000
744
985
1000
783
988
1000
969
0.04

RGS, QP

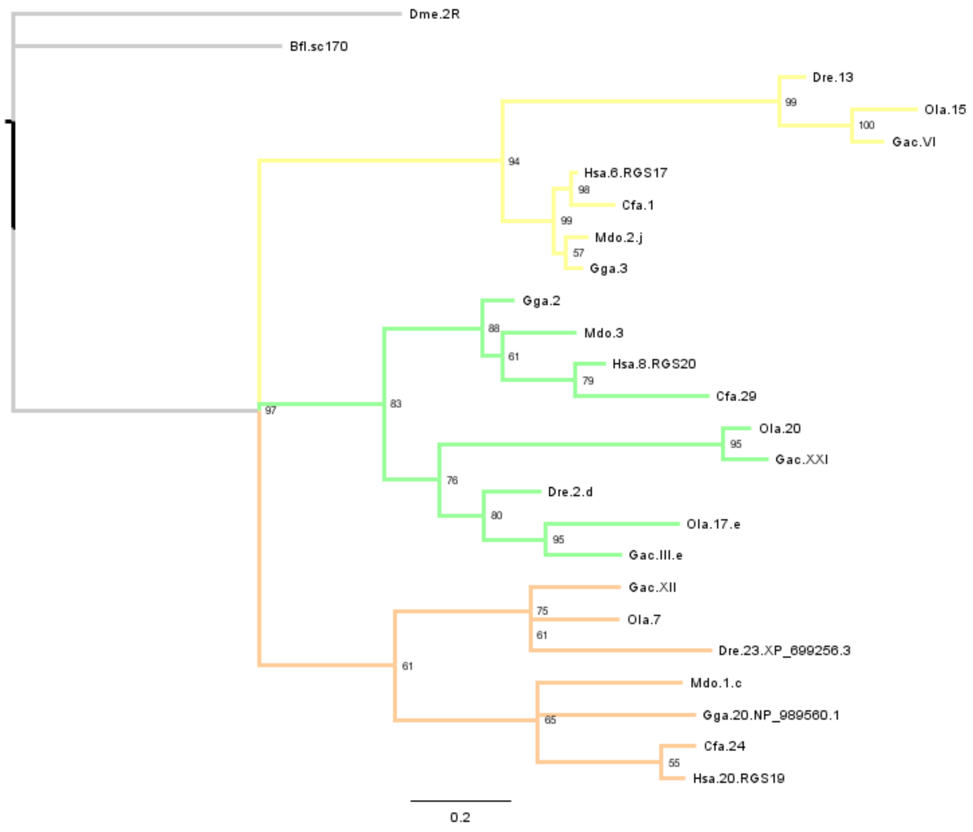
Dme.2R
Bfl.sc170
Dre.13
Ola.15
Gac.VI
Hsa.6.RGS17
Cfa.1
Mdo.2.j
Gga.3
Gga.2
Mdo.3
Hsa.8.RGS20
Cfa.29
Ola.20
Gac.XXI
Dre.2.d
Ola.17.e
Gac.III.e
Gac.XII
Ola.7
Dre.23.XP_699256.3
Mdo.1.c
Gga.20.NP_989560.1
Cfa.24
Hsa.20.RGS19
99
100
94
98
99
57
88
61
79
97
83
95
76
80
95
75
61
61
65
55
0.2

RIMS, NJ

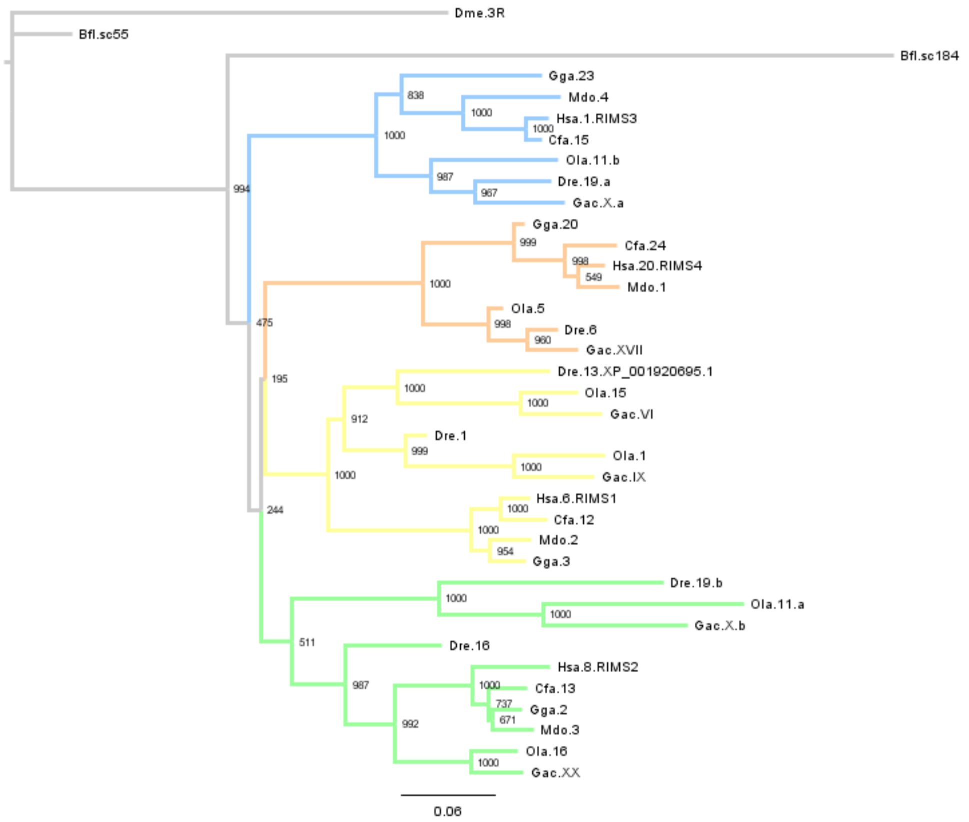

RIMS, QP

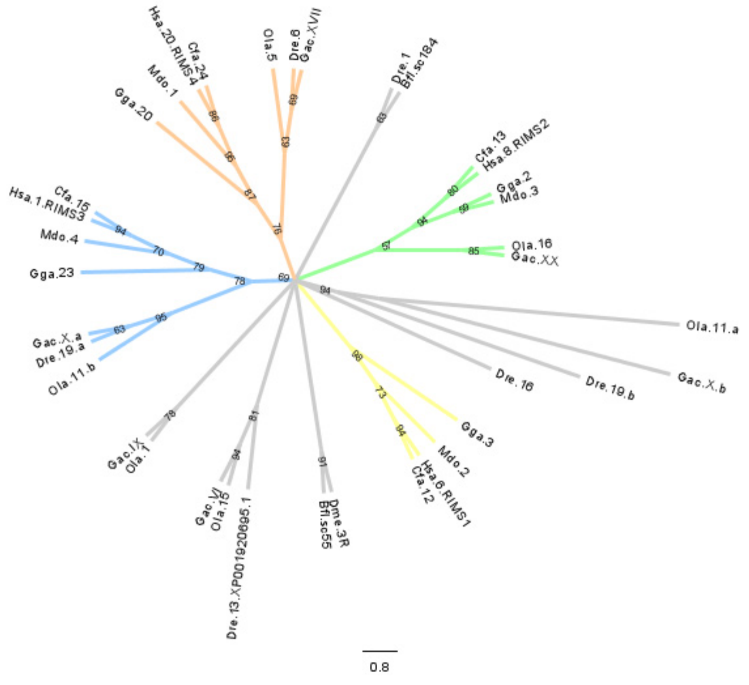

RSPO, NJ

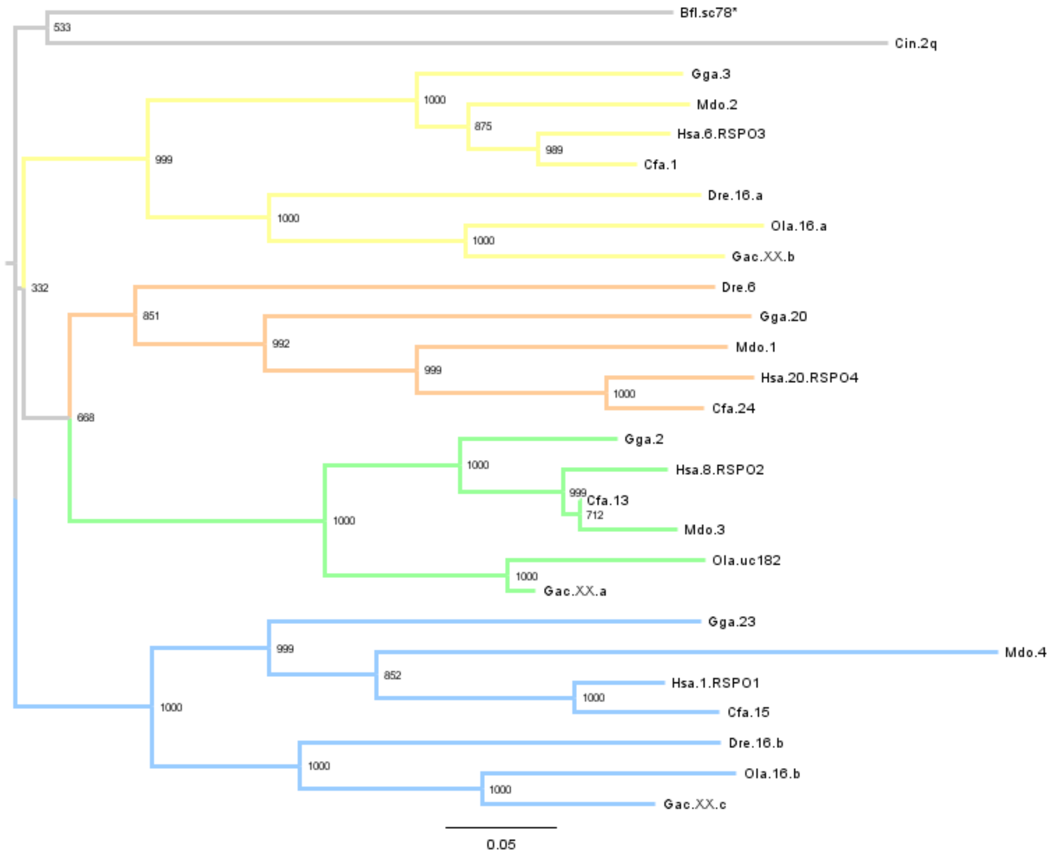

RSPO, QP

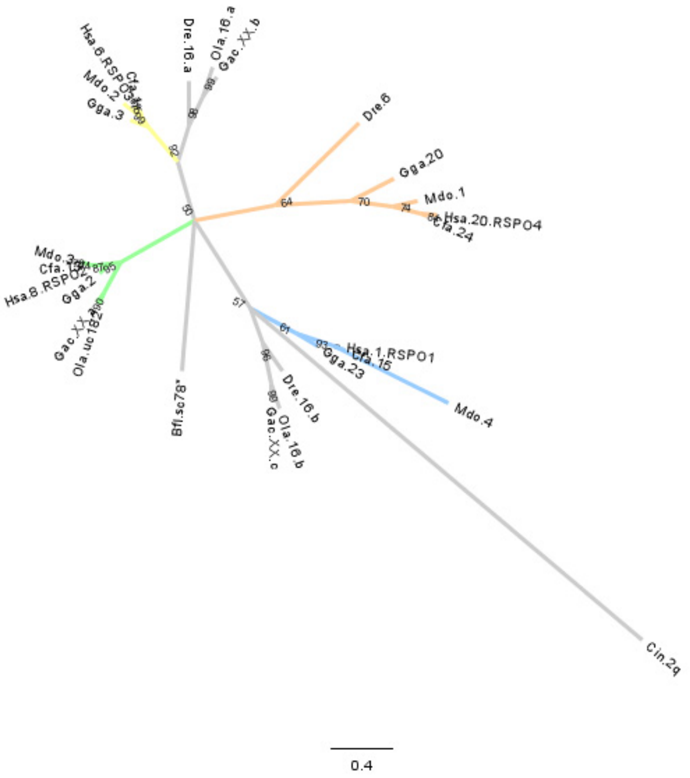

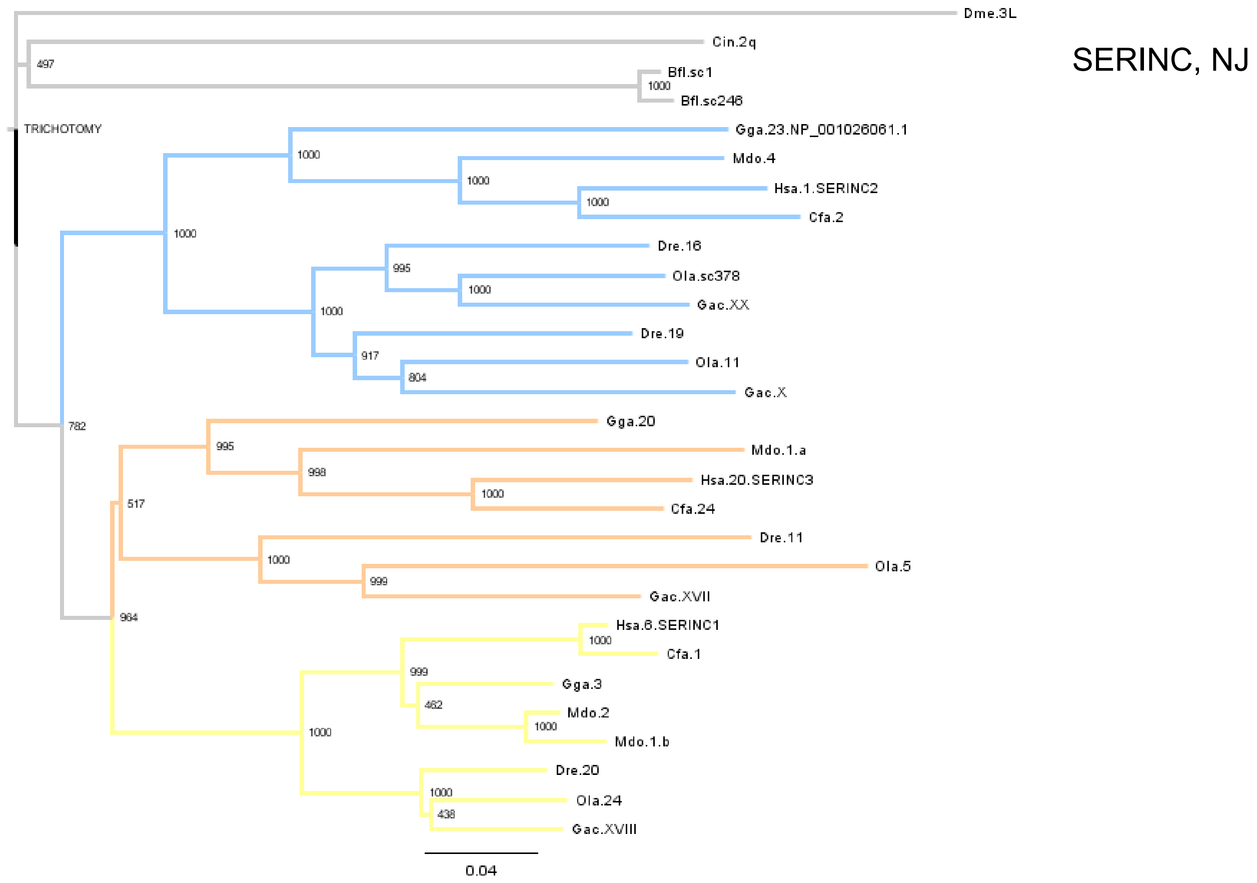

SERINC, NJ
Dme.3L
Cin.2q
497
Bfl.sc1
1000
Bfl.sc246
TRICHOTOMY
Gga.23.NP_001026061.1
1000
Mdo.4
1000
Hsa.1.SERINC2
1000
Cfa.2
1000
Dre.16
995
Ola.sc378
1000
Gac.XX
1000
Dre.19
917
Ola.11
804
Gac.X
782
Gga.20
995
Mdo.1.a
998
Hsa.20.SERINC3
1000
Cfa.24
517
Dre.11
1000
Ola.5
999
Gac.XVII
964
Hsa.6.SERINC1
1000
Cfa.1
999
Gga.3
462
Mdo.2
1000
Mdo.1.b
1000
Dre.20
1000
Ola.24
438
Gac.XVIII
0.04

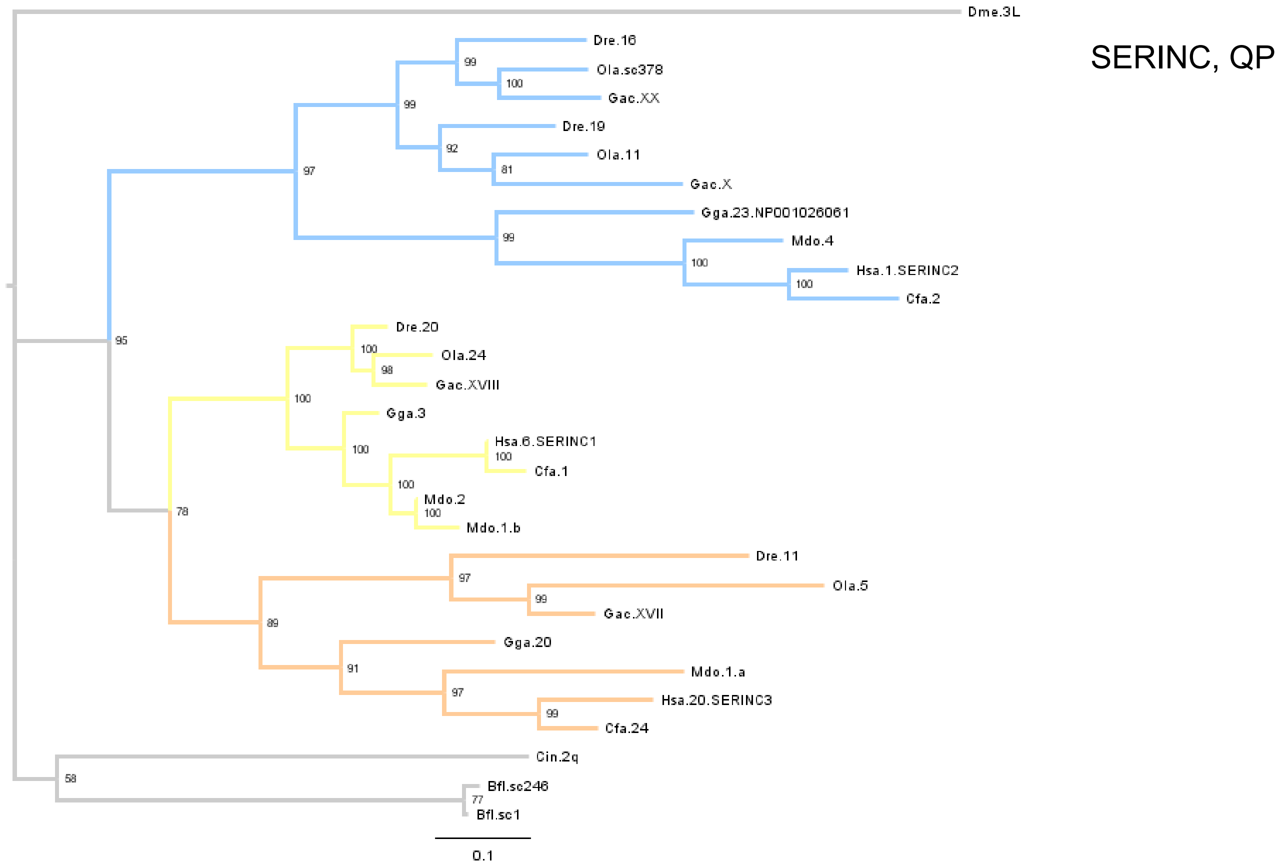

SERINC, QP
Dme.3L
Dre.16
99
Ola.sc378
100
Gac.XX
99
Dre.19
92
Ola.11
81
Gac.X
97
Gga.23.NP001026061
99
Mdo.4
100
Hsa.1.SERINC2
100
Cfa.2
95
Dre.20
100
Ola.24
98
Gac.XVIII
100
Gga.3
100
Hsa.6.SERINC1
100
Cfa.1
100
Mdo.2
100
Mdo.1.b
78
Dre.11
97
Ola.5
99
Gac.XVII
89
Gga.20
91
Mdo.1.a
97
Hsa.20.SERINC3
99
Cfa.24
Cin.2q
58
Bfl.sc246
77
Bfl.sc1
0.1

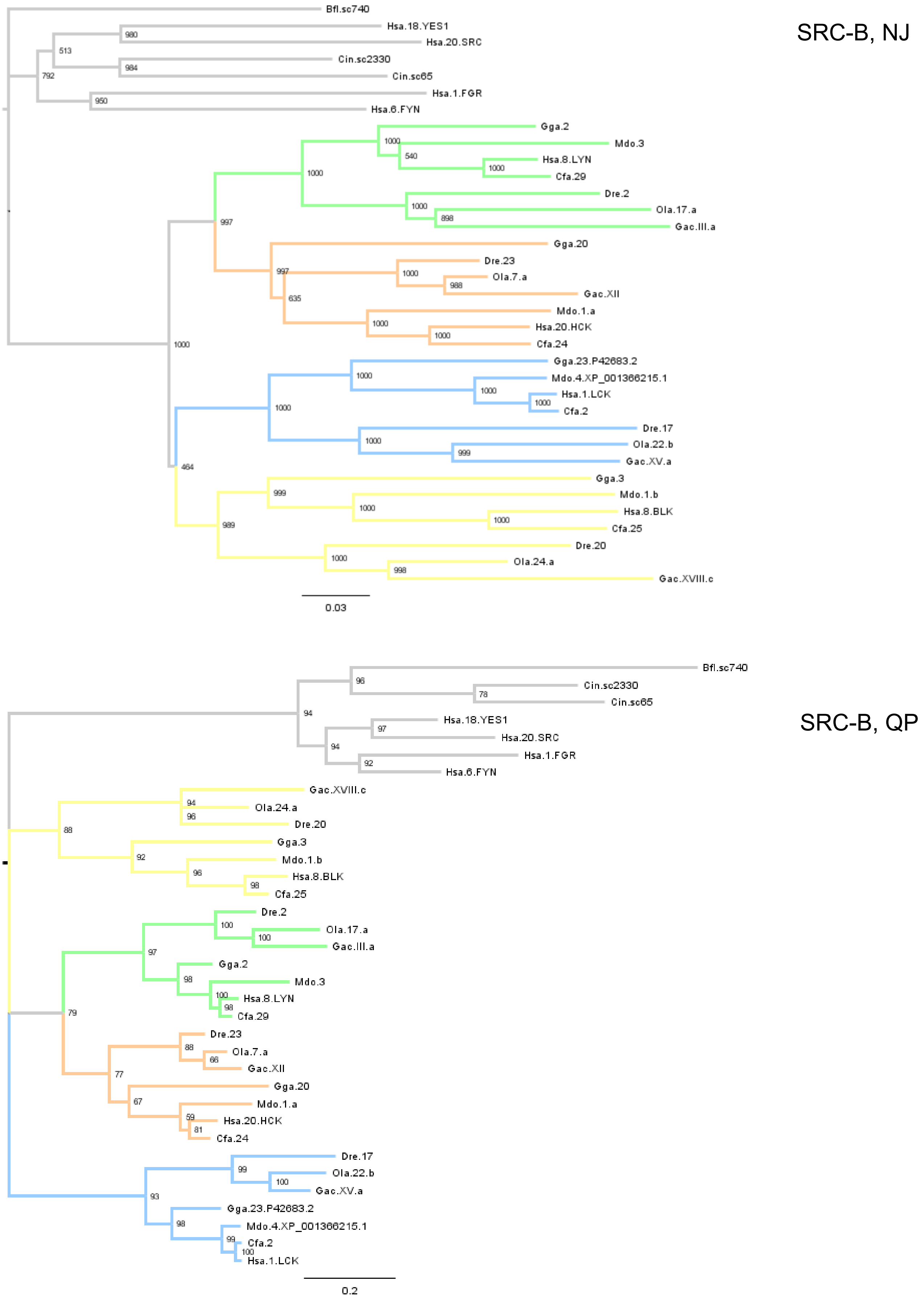
SRC-B, NJ
Bfl.sc740
Hsa.18.YES1
Hsa.20.SRC
Cin.sc2330
Cin.sc65
Hsa.1.FGR
Hsa.6.FYN
Gga.2
Mdo.3
Hsa.8.LYN
Cfa.29
Dre.2
Ola.17.a
Gac.III.a
Gga.20
Dre.23
Ola.7.a
Gac.XII
Mdo.1.a
Hsa.20.HCK
Cfa.24
Gga.23.P42683.2
Mdo.4.XP_001366215.1
Hsa.1.LCK
Cfa.2
Dre.17
Ola.22.b
Gac.XV.a
Gga.3
Mdo.1.b
Hsa.8.BLK
Cfa.25
Dre.20
Ola.24.a
Gac.XVIII.c
0.03
SRC-B, QP
Bfl.sc740
Cin.sc2330
Cin.sc65
Hsa.18.YES1
Hsa.20.SRC
Hsa.1.FGR
Hsa.6.FYN
Gac.XVIII.c
Ola.24.a
Dre.20
Gga.3
Mdo.1.b
Hsa.8.BLK
Cfa.25
Dre.2
Ola.17.a
Gac.III.a
Gga.2
Mdo.3
Hsa.8.LYN
Cfa.29
Dre.23
Ola.7.a
Gac.XII
Gga.20
Mdo.1.a
Hsa.20.HCK
Cfa.24
Dre.17
Ola.22.b
Gac.XV.a
Gga.23.P42683.2
Mdo.4.XP_001366215.1
Cfa.2
Hsa.1.LCK
0.2

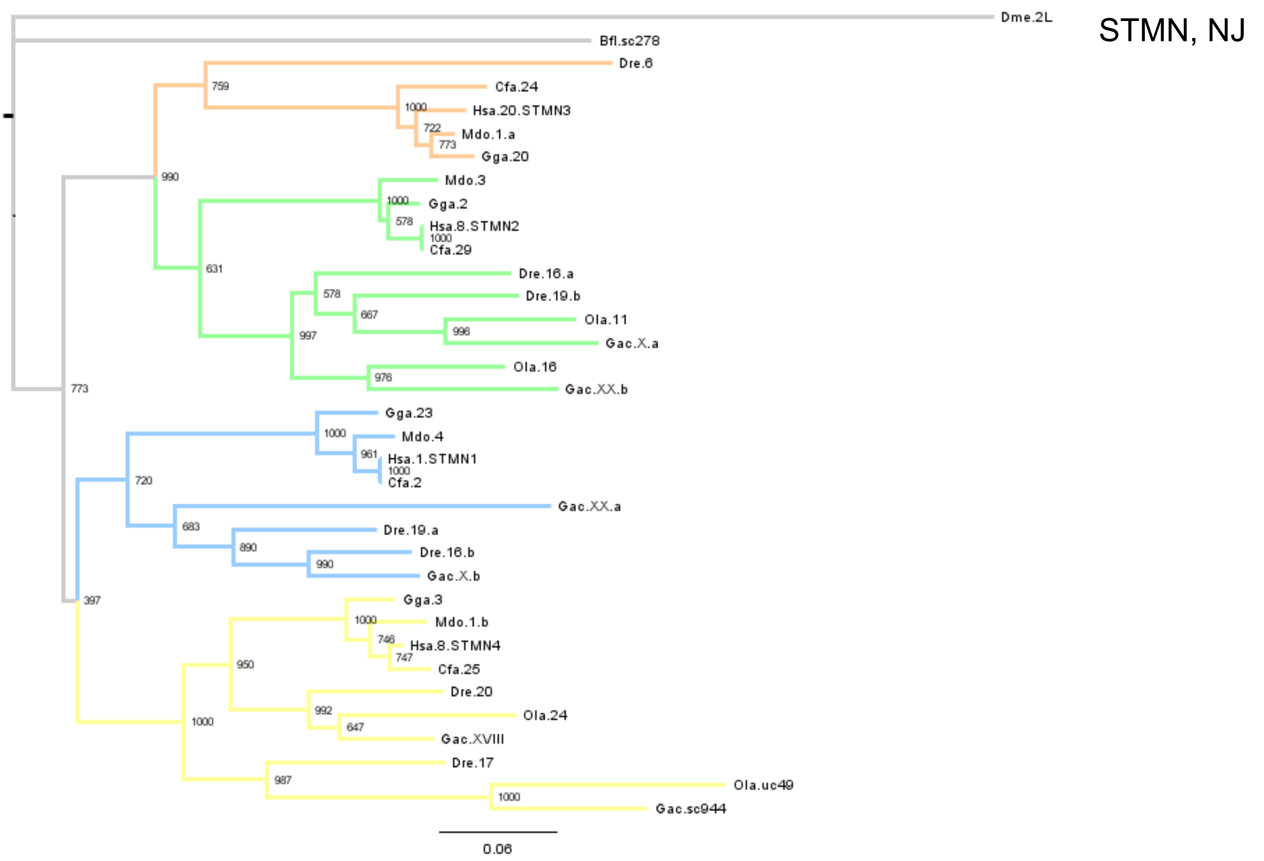
STMN, NJ
Dme.2L
Bfl.sc278
Dre.6
Cfa.24
Hsa.20.STMN3
Mdo.1.a
Gga.20
Mdo.3
Gga.2
Hsa.8.STMN2
Cfa.29
Dre.16.a
Dre.19.b
Ola.11
Gac.X.a
Ola.16
Gac.XX.b
Gga.23
Mdo.4
Hsa.1.STMN1
Cfa.2
Gac.XX.a
Dre.19.a
Dre.16.b
Gac.X.b
Gga.3
Mdo.1.b
Hsa.8.STMN4
Cfa.25
Dre.20
Ola.24
Gac.XVIII
Dre.17
Ola.uc49
Gac.sc944
0.06

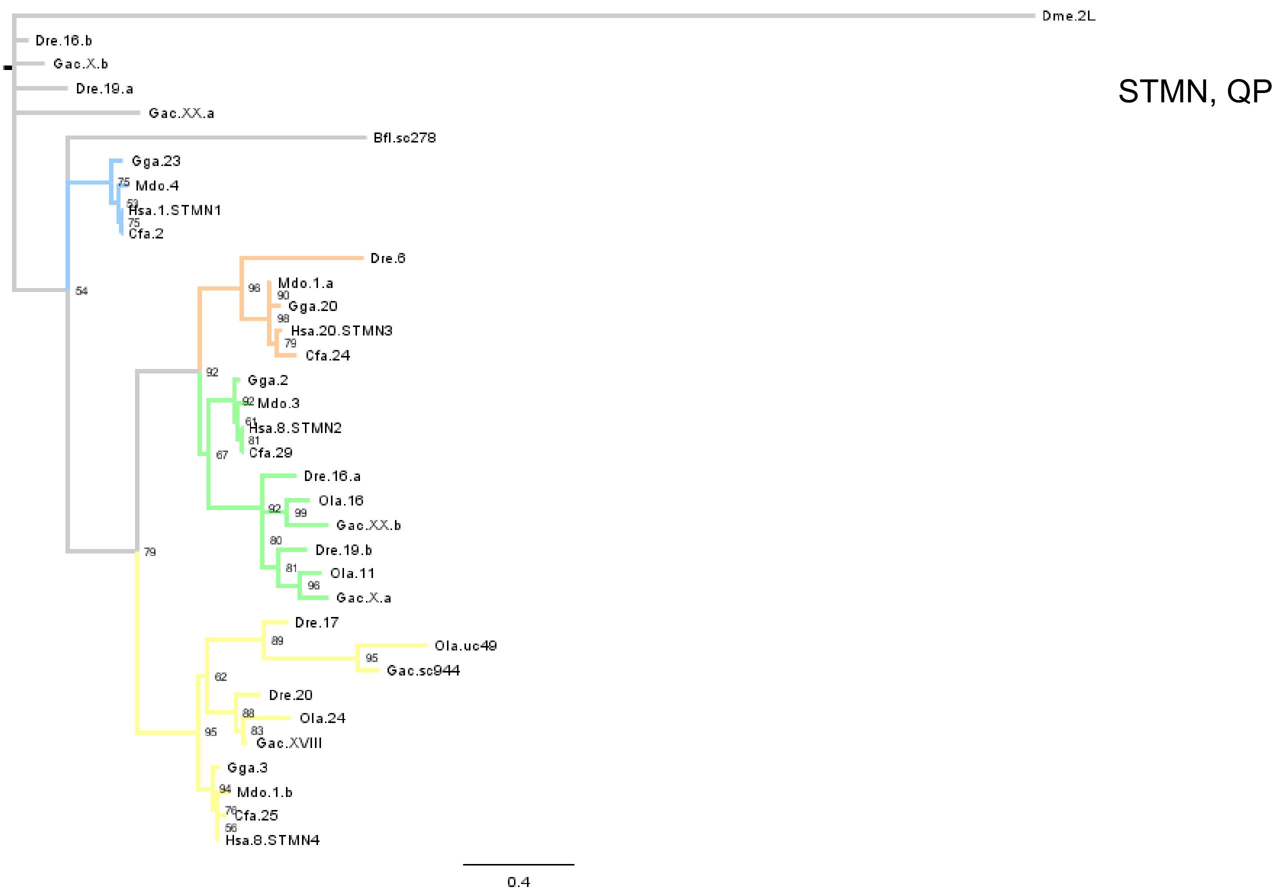
STMN, QP
Dme.2L
Dre.16.b
Gac.X.b
Dre.19.a
Gac.XX.a
Bfl.sc278
Gga.23
Mdo.4
Hsa.1.STMN1
Cfa.2
Dre.6
Mdo.1.a
Gga.20
Hsa.20.STMN3
Cfa.24
Gga.2
Mdo.3
Hsa.8.STMN2
Cfa.29
Dre.16.a
Ola.16
Gac.XX.b
Dre.19.b
Ola.11
Gac.X.a
Dre.17
Ola.uc49
Gac.sc944
Dre.20
Ola.24
Gac.XVIII
Gga.3
Mdo.1.b
Cfa.25
Hsa.8.STMN4
0.4

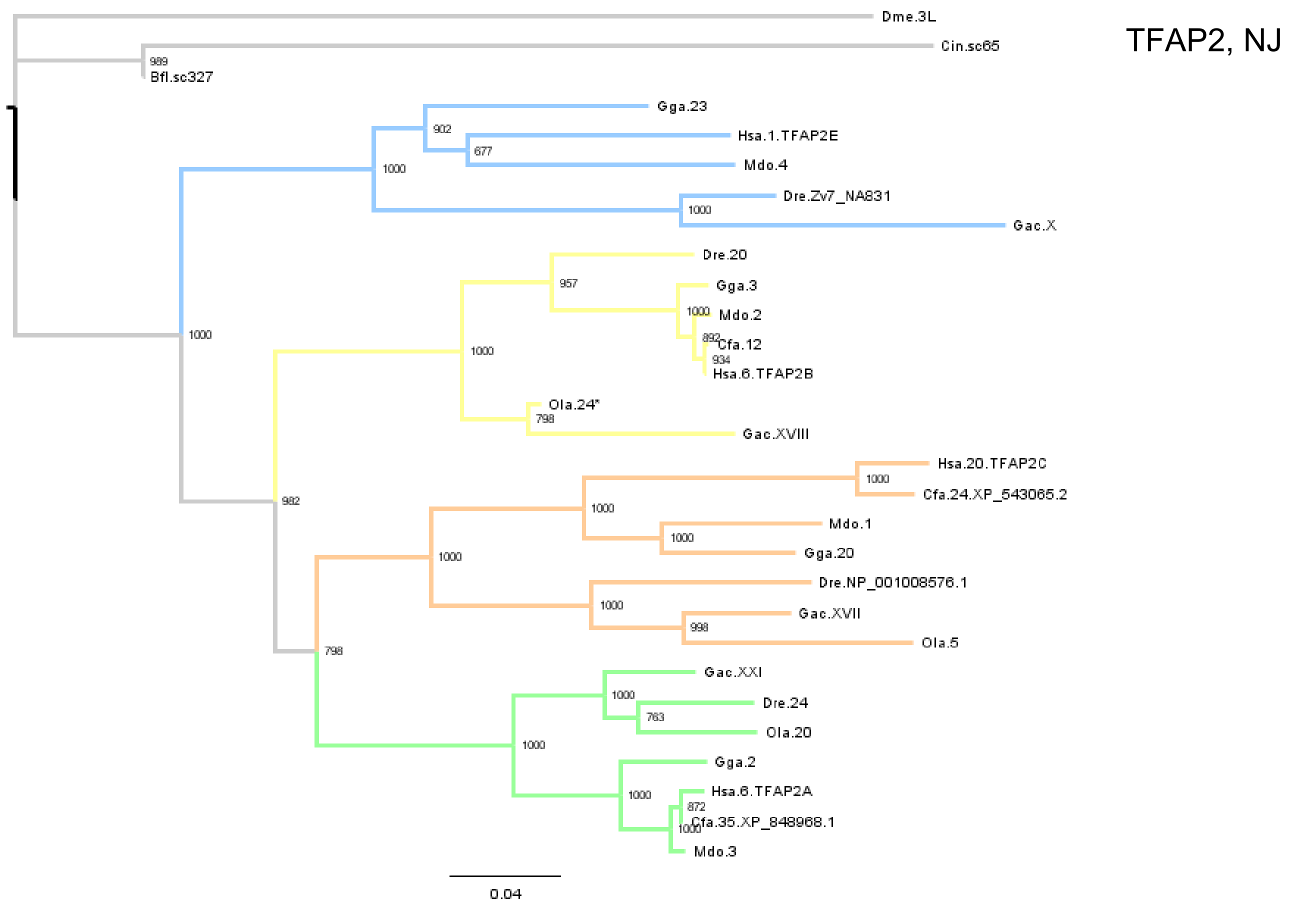
TFAP2, NJ
Dme.3L
Cin.sc65
989
Bfl.sc327
Gga.23
902
Hsa.1.TFAP2E
677
Mdo.4
1000
Dre.Zv7_NA831
1000
Gac.X
Dre.20
957
Gga.3
1000
Mdo.2
892
Cfa.12
934
Hsa.6.TFAP2B
1000
1000
Ola.24*
798
Gac.XVIII
Hsa.20.TFAP2C
1000
Cfa.24.XP_543065.2
1000
Mdo.1
1000
Gga.20
982
1000
Dre.NP_001008576.1
1000
Gac.XVII
998
Ola.5
798
Gac.XXI
1000
Dre.24
763
Ola.20
1000
Gga.2
Hsa.6.TFAP2A
1000
872
Cfa.35.XP_848968.1
1000
Mdo.3
0.04
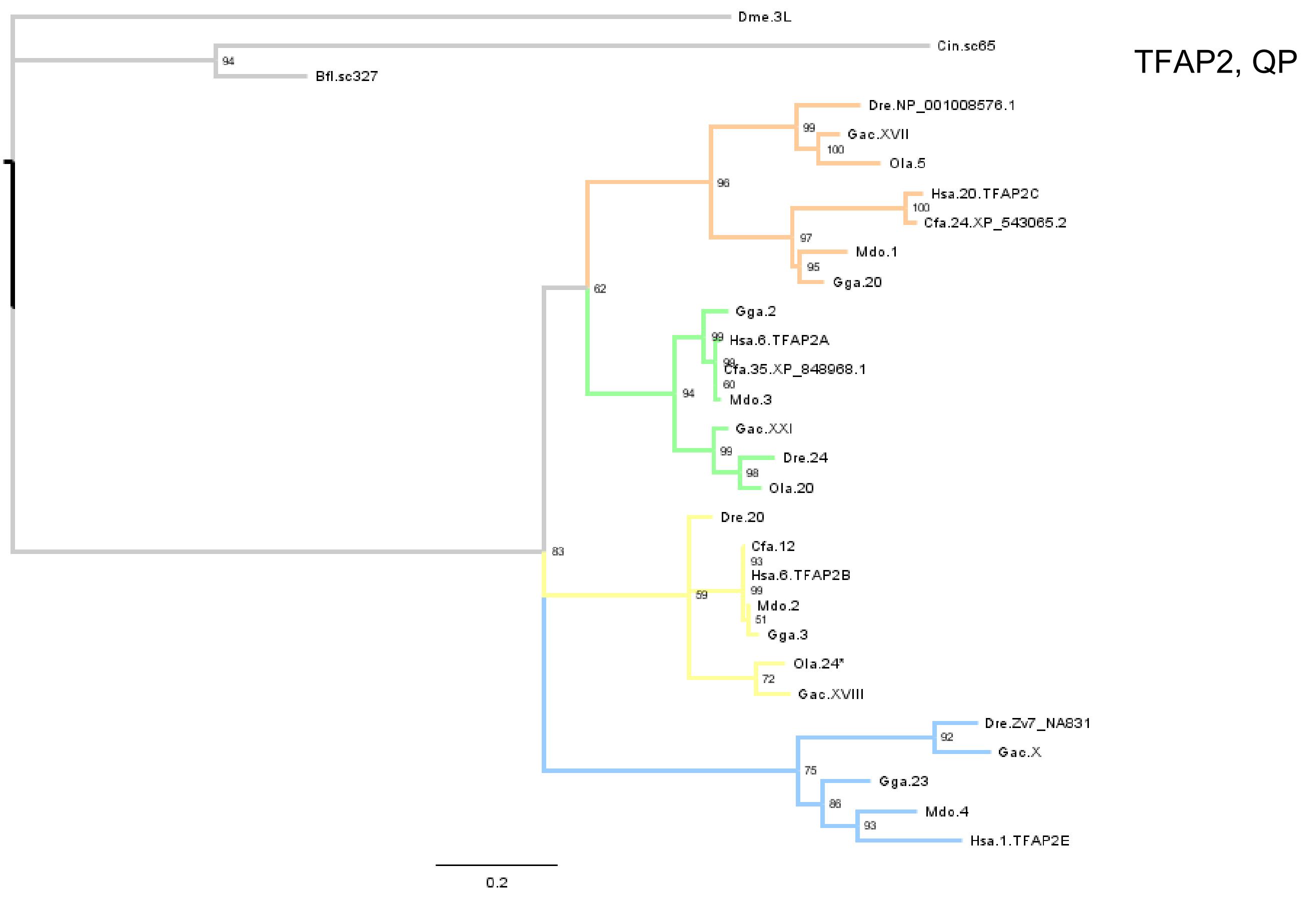
TFAP2, QP
Dme.3L
Cin.sc65
94
Bfl.sc327
Dre.NP_001008576.1
99
Gac.XVII
100
Ola.5
96
Hsa.20.TFAP2C
100
Cfa.24.XP_543065.2
97
Mdo.1
95
Gga.20
62
Gga.2
99
Hsa.6.TFAP2A
98
Cfa.35.XP_848968.1
60
Mdo.3
94
Gac.XXI
99
Dre.24
98
Ola.20
Dre.20
Cfa.12
93
Hsa.6.TFAP2B
99
Mdo.2
51
Gga.3
83
59
Ola.24*
72
Gac.XVIII
Dre.Zv7_NA831
92
Gac.X
75
Gga.23
86
Mdo.4
93
Hsa.1.TFAP2E
0.2

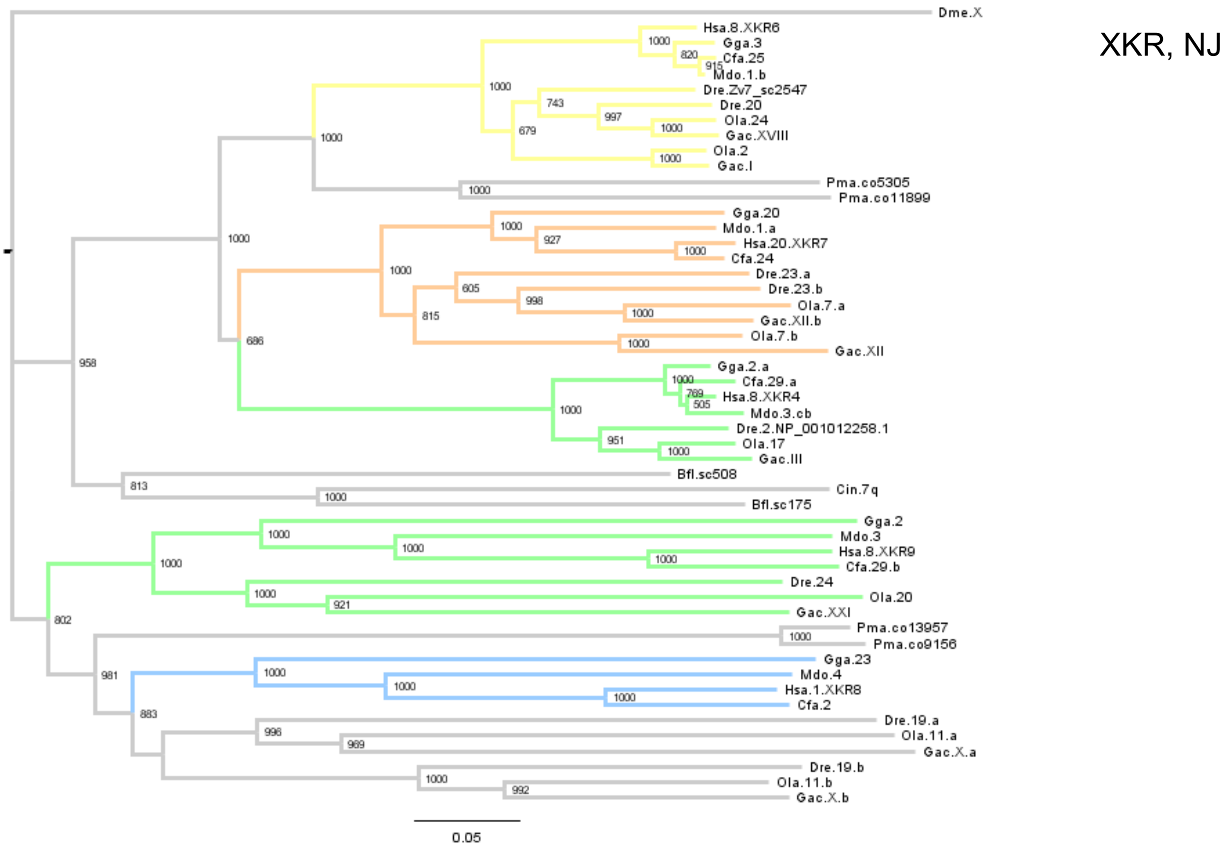
XKR, NJ
Dme.X
Hsa.8.XKR6
Gga.3
Cfa.25
Mdo.1.b
Dre.Zv7_sc2547
Dre.20
Ola.24
Gac.XVIII
Ola.2
Gac.I
Pma.co5305
Pma.co11899
Gga.20
Mdo.1.a
Hsa.20.XKR7
Cfa.24
Dre.23.a
Dre.23.b
Ola.7.a
Gac.XII.b
Ola.7.b
Gac.XII
Gga.2.a
Cfa.29.a
Hsa.8.XKR4
Mdo.3.cb
Dre.2.NP_001012258.1
Ola.17
Gac.III
Bfl.sc508
Cin.7q
Bfl.sc175
Gga.2
Mdo.3
Hsa.8.XKR9
Cfa.29.b
Dre.24
Ola.20
Gac.XXI
Pma.co13957
Pma.co9156
Gga.23
Mdo.4
Hsa.1.XKR8
Cfa.2
Dre.19.a
Ola.11.a
Gac.X.a
Dre.19.b
Ola.11.b
Gac.X.b
0.05

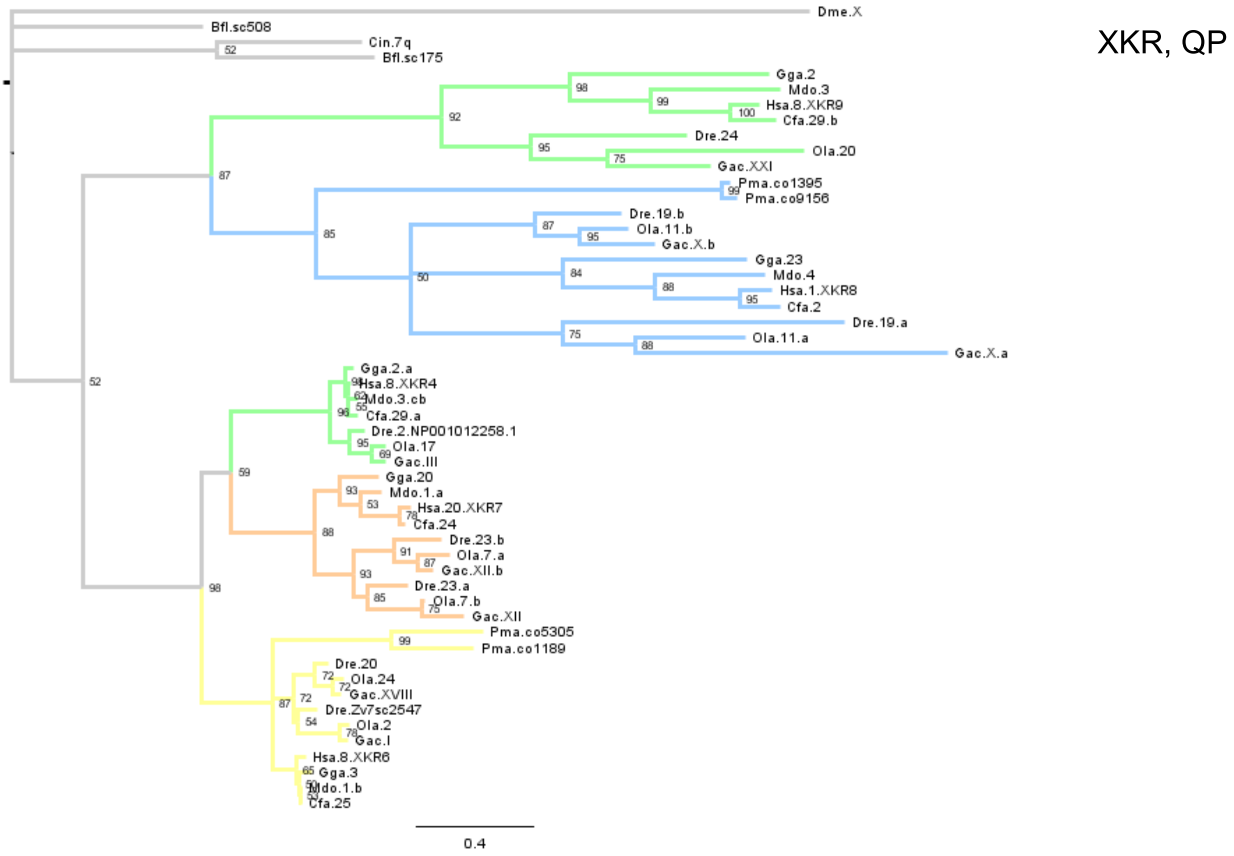
XKR, QP
Dme.X
Bfl.sc508
Cin.7q
Bfl.sc175
Gga.2
Mdo.3
Hsa.8.XKR9
Cfa.29.b
Dre.24
Ola.20
Gac.XXI
Pma.co1395
Pma.co9156
Dre.19.b
Ola.11.b
Gac.X.b
Gga.23
Mdo.4
Hsa.1.XKR8
Cfa.2
Dre.19.a
Ola.11.a
Gac.X.a
Gga.2.a
Hsa.8.XKR4
Mdo.3.cb
Cfa.29.a
Dre.2.NP001012258.1
Ola.17
Gac.III
Gga.20
Mdo.1.a
Hsa.20.XKR7
Cfa.24
Dre.23.b
Ola.7.a
Gac.XII.b
Dre.23.a
Ola.7.b
Gac.XII
Pma.co5305
Pma.co1189
Dre.20
Ola.24
Gac.XVIII
Dre.Zv7sc2547
Ola.2
Gac.I
Hsa.8.XKR6
Gga.3
Mdo.1.b
Cfa.25
0.4

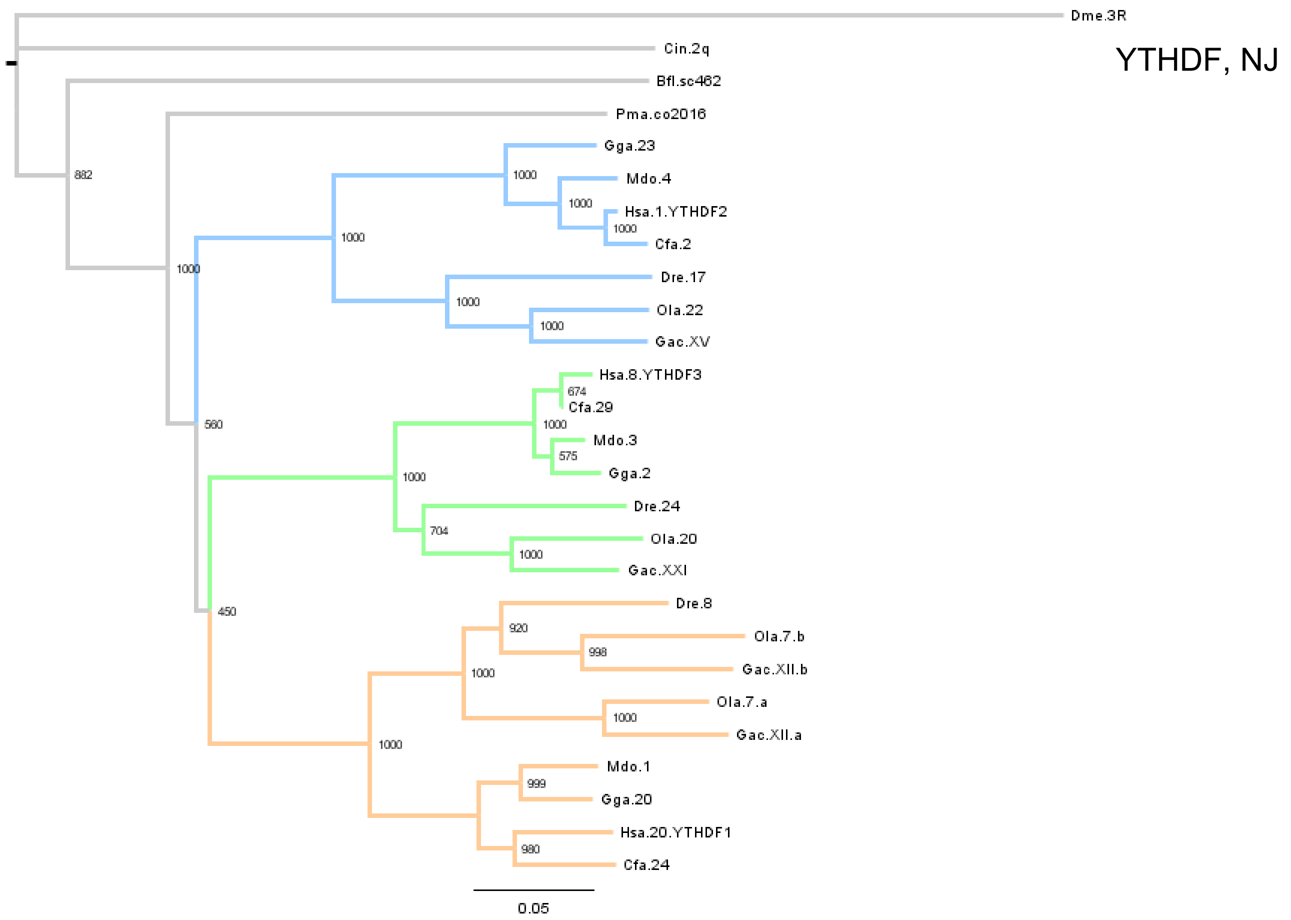

YTHDF, NJ
Dme.3R
Cin.2q
Bfl.sc462
Pma.co2016
Gga.23
Mdo.4
Hsa.1.YTHDF2
Cfa.2
Dre.17
Ola.22
Gac.XV
Hsa.8.YTHDF3
Cfa.29
Mdo.3
Gga.2
Dre.24
Ola.20
Gac.XXI
Dre.8
Ola.7.b
Gac.XII.b
Ola.7.a
Gac.XII.a
Mdo.1
Gga.20
Hsa.20.YTHDF1
Cfa.24
882
1000
560
450
674
575
704
920
998
999
980
0.05

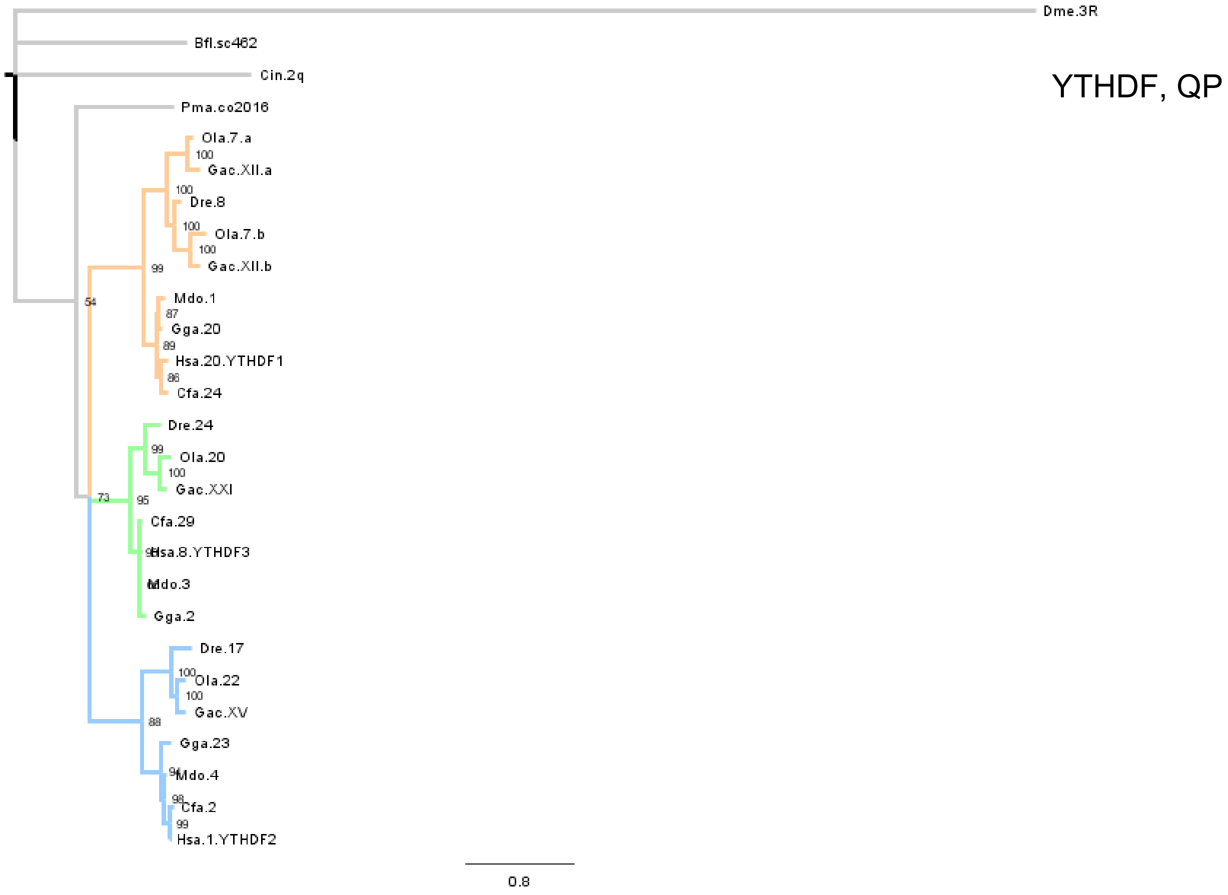

YTHDF, QP
Dme.3R
Bfl.sc462
Cin.2q
Pma.co2016
Ola.7.a
Gac.XII.a
Dre.8
Ola.7.b
Gac.XII.b
Mdo.1
Gga.20
Hsa.20.YTHDF1
Cfa.24
Dre.24
Ola.20
Gac.XXI
Cfa.29
Hsa.8.YTHDF3
Mdo.3
Gga.2
Dre.17
Ola.22
Gac.XV
Gga.23
Mdo.4
Cfa.2
Hsa.1.YTHDF2
100
99
54
87
89
86
73
95
88
98
0.8
